# LysipheN: a gravimetric IoT device for near real-time high-frequency crop phenotyping: a case study on common beans

**DOI:** 10.1186/s13007-024-01170-x

**Published:** 2024-03-14

**Authors:** Duvan Pineda-Castro, Harold Diaz, Jonatan Soto, Milan Oldřich Urban

**Affiliations:** The Alliance of Bioversity International and CIAT, Km 17 Recta Cali-Palmira, Apartado Aereo 7613, Cali, 763537 Colombia

**Keywords:** Phenotyping, Precision agriculture, Mechatronic design, Transpiration, Low-cost, Target populations of environment, Target-specific breeding, AgriTech

## Abstract

Climate instability directly affects agro-environments. Water scarcity, high air temperature, and changes in soil biota are some factors caused by environmental changes. Verified and precise phenotypic traits are required for assessing the impact of various stress factors on crop performance while keeping phenotyping costs at a reasonable level. Experiments which use a lysimeter method to measure transpiration efficiency are often expensive and require complex infrastructures. This study presents the development and testing process of an automated, reliable, small, and low-cost prototype system using IoT with high-frequency potential in near-real time. Because of its waterproofness, our device—LysipheN—assesses each plant individually and can be deployed for experiments in different environmental conditions (farm, field, greenhouse, etc.). LysipheN integrates multiple sensors, automatic irrigation according to desired drought scenarios, and a remote, wireless connection to monitor each plant and device performance via a data platform. During testing, LysipheN proved to be sensitive enough to detect and measure plant transpiration, from early to ultimate plant developmental stages. Even though the results were generated on common beans, the LysipheN can be scaled up/adapted to other crops. This tool serves to screen transpiration, transpiration efficiency, and transpiration-related physiological traits. Because of its price, endurance, and waterproof design, LysipheN will be useful in screening populations in a realistic ecological and breeding context. It operates by phenotyping the most suitable parental lines, characterizing genebank accessions, and allowing breeders to make a target-specific selection using functional traits (related to the place where LysipheN units are located) in line with a realistic agronomic background.

**Supplementary Information:**

The online version contains supplementary material available at 10.1186/s13007-024-01170-x.

## Background

Excessive costs of water and/or limited access to it in some agricultural areas do not allow all producers to access available solutions [1, 2]. Breeders are constantly searching to identify new phenotypic traits in early-generation materials that can indicate drought resistance, and/or water-use efficiency, and allow higher crop yields and better seed quality while keeping production costs low [3, 4]. This is a challenging task, especially when dealing with a wide range of possible adaptations or extremely different final product quality (seeds). On the other hand, the room for crop adaptation is limited by climate and climate-change volatility. Furthermore, there is not enough precise information about how crops will respond to these changes [5]. In the case of common beans, drought stress (and its consequences) has a higher level of severity than other types of abiotic stresses to which the bean may be exposed [6]. However, these problems are present in many other crops.

Experiments in diverse but well-described and well-managed conditions help researchers to determine crop behavior related to a given environment [7]. Experiments focused on important crops need to be accompanied by internal and external information on crop physiological reactions. The whole set of important responses is related to restricted water availability, which should be understood by compiling both physical and climatic components; different soil types and soil profile depths; plant density, and crop-atmosphere relationships [8]. The physiologically adequate and agronomically relevant stress timing and duration connected with high-frequency data will play a crucial role in understanding crop responses.

Thus, crops’ water use has become a main focus and carries different concepts to be considered. Therefore, to define water productivity at the plant level, transpiration efficiency (TE) likely plays a crucial role as it is an important component of the concept called effective use of water (EUW). TE is defined by accumulated economic biomass (i.e., seeds) per water unit transpired via the plant body [9]. To measure TE, a long period of plant observation/weighing is an indispensable need during the entire crop cycle. Without automation, this work has a drudgery component. Additionally, [10] emphasize that EUW studies should not be the only factor in optimizing yield under limited water conditions; Understanding the role of both limited and non-restricted transpiration during the diurnal phase and also across the whole phenology is one of the most important questions connected to target-specific breeding. Crop growth models recognize that water scarcity is associated with a high vapor pressure deficit (VPD), which in turn implies a condition of atmospheric water stress [11]. The dehydration resistance of leaves to very high VPDs has become a trait that researchers suggest being included when considering breeding crop varieties adapted to water-limited environments [12]. On the other hand, under drought conditions, a limited transpiration rate under high VPD could be used to conserve soil water for later use when seeds are filled [13]. Logically the soil water availability and dynamic changes in water consumption during different phenological steps are crucial factors in determining the impact of VPD on crop development and should be included in analyses [14].

The challenge of useful phenotyping is aimed at techniques, tools, and databases together with the entire phenotypic platforms which rely on “hypothesis-driven” rather than “available tool-driven” research outputs [15]. The lysimeters measure changes in water movements by weighing the entire system. However, because of their robustness and complexity, most designs are considered impractical and expensive thus they are not widely used in research centers [16]. Additionally, the weighing lysimeter is a very accurate method for studying crop water-related requirements and gives valuable data for improving crop water management. Understanding this, some agronomists or crop scientists use pots or trays in evapotranspiration calculations [17, 18]. Undoubtedly, there is a constantly increasing need for plant-phenotyping systems, which can (semi-)automatically measure water movement and calculate water use to identify genotypes with improved EUW [19].

In support of the above-mentioned considerations, the integration of technology to study plant features has reached the point at which novelty robotics solutions and mechatronic systems have often been offered [20]. Mechatronic systems that analyze plant-pot systems with (semi-)controlled irrigation are the next logical step to go further to find conservative crops with a limited transpiration rate at high VPDs [13]. Since VPD appears to have the greatest influence on transpiration response compared to other environmental variables [12], different studies have presented their prototypes reflecting VPD values. One of these is the lysimeter developed with a water-use-optimized irrigation system developed by Vera-Repullo. The study includes irrigation determined from the lysimeter’s dataset processing and an embedded system controller which is used as a remote data logger and allows data information access through the internet [21]. However, it is crucial that researchers focus efforts on developing tools that may be used directly in field conditions, not only in regulated and protected greenhouses. Some authors [16] have presented a weighing lysimeter prototype at the field level which was used on the farm and placed in the soil.

Solutions implemented so far also include remote-sensing systems (i.e., wireless sensors with dataloggers), which have become an important tool for crop scientists. These technologies allow for capturing standardized data, reducing time-demanding operations, and eliminating differences in individual data perception [15]. Therefore, it is common that remote systems to be integrated with the internet-of-things (IoT). One example of this is the solution shown by [17], which implemented a low-cost weighing lysimeter that was able to measure the weight of potted crops and use an open-source platform to determine water balance in irrigation periods. Data were sent remotely to the cloud and accessible via the internet. In that way, [22] the system used 100 balances (scales) inside a glasshouse where each one automatically logged weight data every minute. In this study [22], two common bean cultivars demonstrate greater drought sensitivity compared to soybean cultivars. Thus, it is demonstrated that gravimetric platforms allow recognizing of the effects of biomass accumulation and transpiration per individual plant, as well as calculations related to the TE at the cycle end when seed yield is available. Through the measurements of plant weight changes, the extracted transpiration rate profiles can identify water-saving and water-spending mechanisms based on daily plant water losses (standardized to actual leaf area or other traits) representing transpiration response to VPD [23].

Our study shows the development and testing process for a solution-oriented tool that combines sensors with IoT technology. We called the device “LysipheN” (also Gravimetric Unit—GU). LysipheN is an automated, low-cost, and novel prototype for measuring the soil–plant-atmosphere continuum responses. By measuring the weight changes of the system (plant sown in a pot with soil) and reducing evaporation by surface coverage, LysipheN can automatically obtain the transpiration amount every minute diurnally or across the entire crop lifecycle. The system recognizes exactly the amount of water that has been irrigated within the system since the beginning of the experiment. The internal tissue (stem and pod temperature), soil moisture, air humidity, and air temperature sensors have been included in the device to capture the climatic data. Electronically, the LysipheN is an autonomous self-standing IoT-embedded system that sends data through the internet and stores a backup of millions of data locally within each unit. Besides that, the Arduino-based integrated system allows the integration of more sensors together. Using a Linux-based operating system (OS) allows updating and identifying the responses of different genotypes to different environments in an almost real-time manner. Each GU is an independent system and can be placed in different sites—growth chambers, greenhouses, and even within (remote) field trials.

The goal of this study is to present a LysipheN system that can identify outstanding parental accessions for a particular region, using lines within a realistic breeding context, connected to product profile information, to dissect water-use-related traits of different genotypes/species and help to characterize genebank accessions. The LysipheN concept is oriented to be universal and versatile across a range of special-interest in crops such as beans, rice, bananas, tropical forage crops, and others in different stress scenarios related to TPEs of interest.

## Results

The research implemented several experiments to achieve secure, highly precise measurements via the developed LysipheN prototype. Several different prototypes were tested (Gravimetric Units). The prototype device is described in “Materials and methods” section. For detailed technological adjustment data, please see Additional file 1. Once the defined version was ready, 10 prototype replicas of LysipheN units were built to be tested. The experiments were conducted using the Bioversity International and CIAT Alliance facilities, inside a greenhouse in controlled conditions and under a monitored environment.

**Fig. 1:**
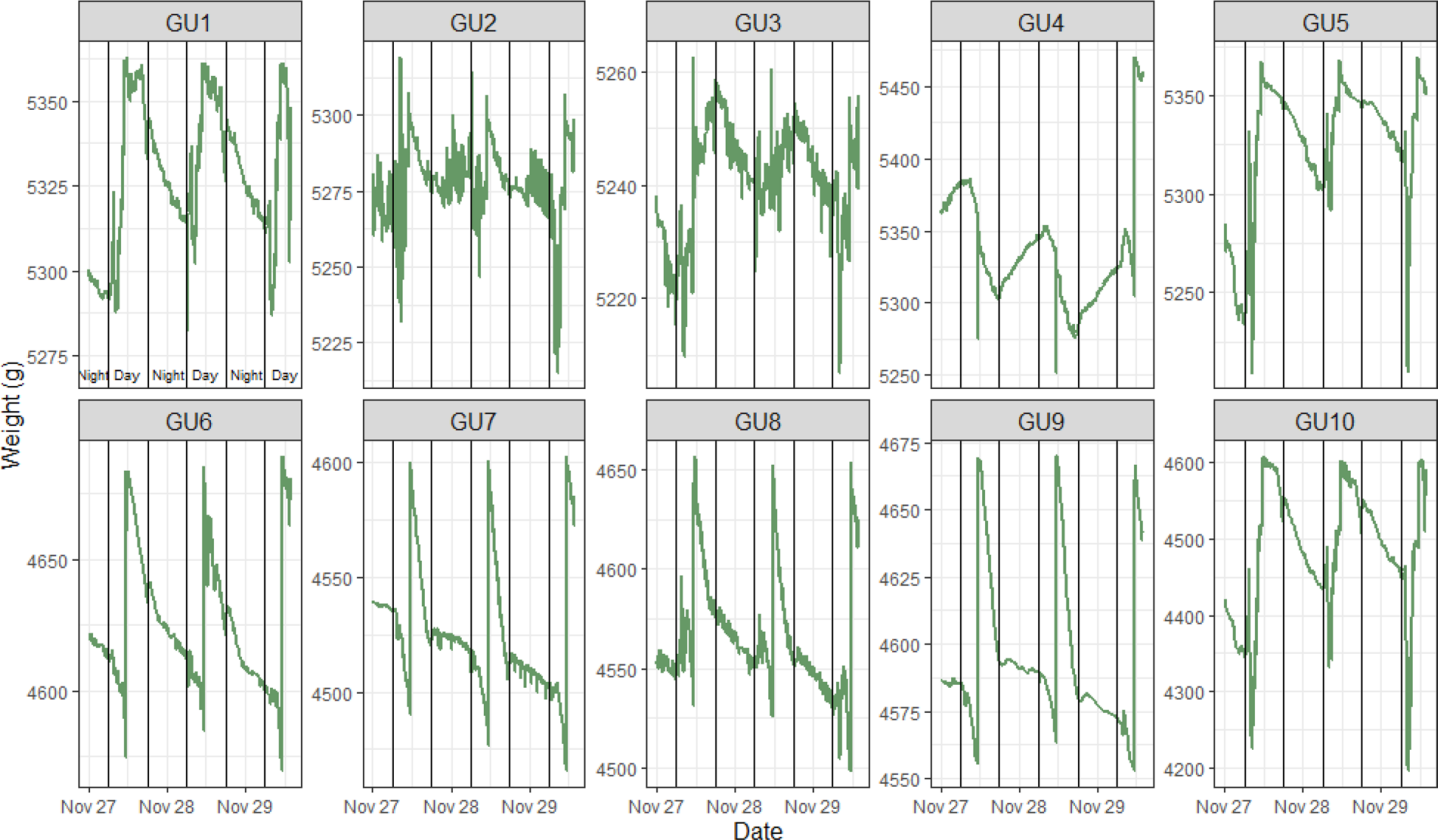
Raw weight data from ten prototypes (evaluation of 3 consecutive days). GU1 to GU5 younger (PCS) plants (1 month after sowing), GU6 to GU10 (PCH) plants (> 80 days after sowing). A remaining noise can be noted on GU2 and GU10 data

**Fig. 2:**
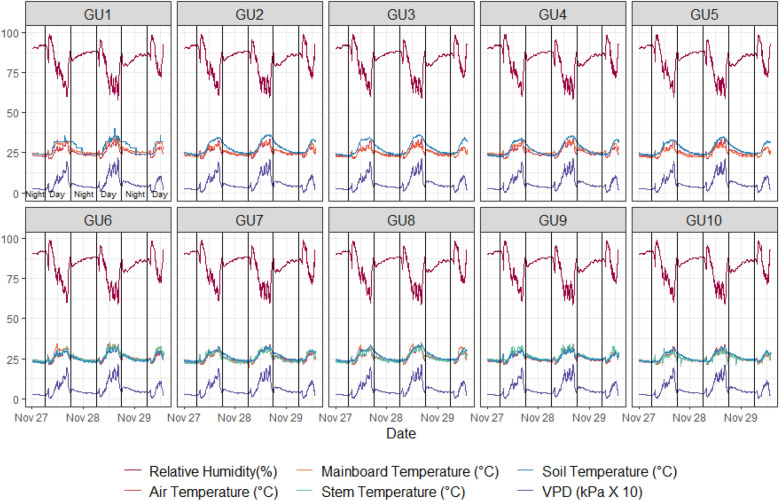
Air temperature, electronic board temperature, the inside stem temperature (inserted thermocouple), and soil temperature together with air RH and calculated VPD. GU1 to GU5 studied younger (PCS) plants (1 month after sowing), and GU6 to GU10 studied plants close to the final harvest (PCH)

The prototypes were set to record data every 5 min. The system weight (Fig. 1), air RH, air temperature, internal stem temperature, and soil temperature (Fig. 2) were monitored. Together with the data from the sensors, every prototype calculates and recognizes data such as bean variety, number of irrigation events, total irrigated volume, etc. These data are stored automatically and sent to the phenotyping platform database. The raw results show that GU’s are stable (1, 2, 3, 5, 6–10), only GU4 shows some noise data (Fig. 1). The irrigation events are clearly visible close to 10 am of each day. Mark higher raw weight changes in younger plants suggesting very high transpiration and water consumption connected to active prolongation growth on the plants.

The results show the environmental and stem temperature data from GUs (Fig. 2). The environmental data are logically pretty similar as the experiment was conducted in the same greenhouse. The only variable here can be visualized in the stem temperature as this is affected by the stem water conductivity and other genotype-related factors.

**Fig. 3:**
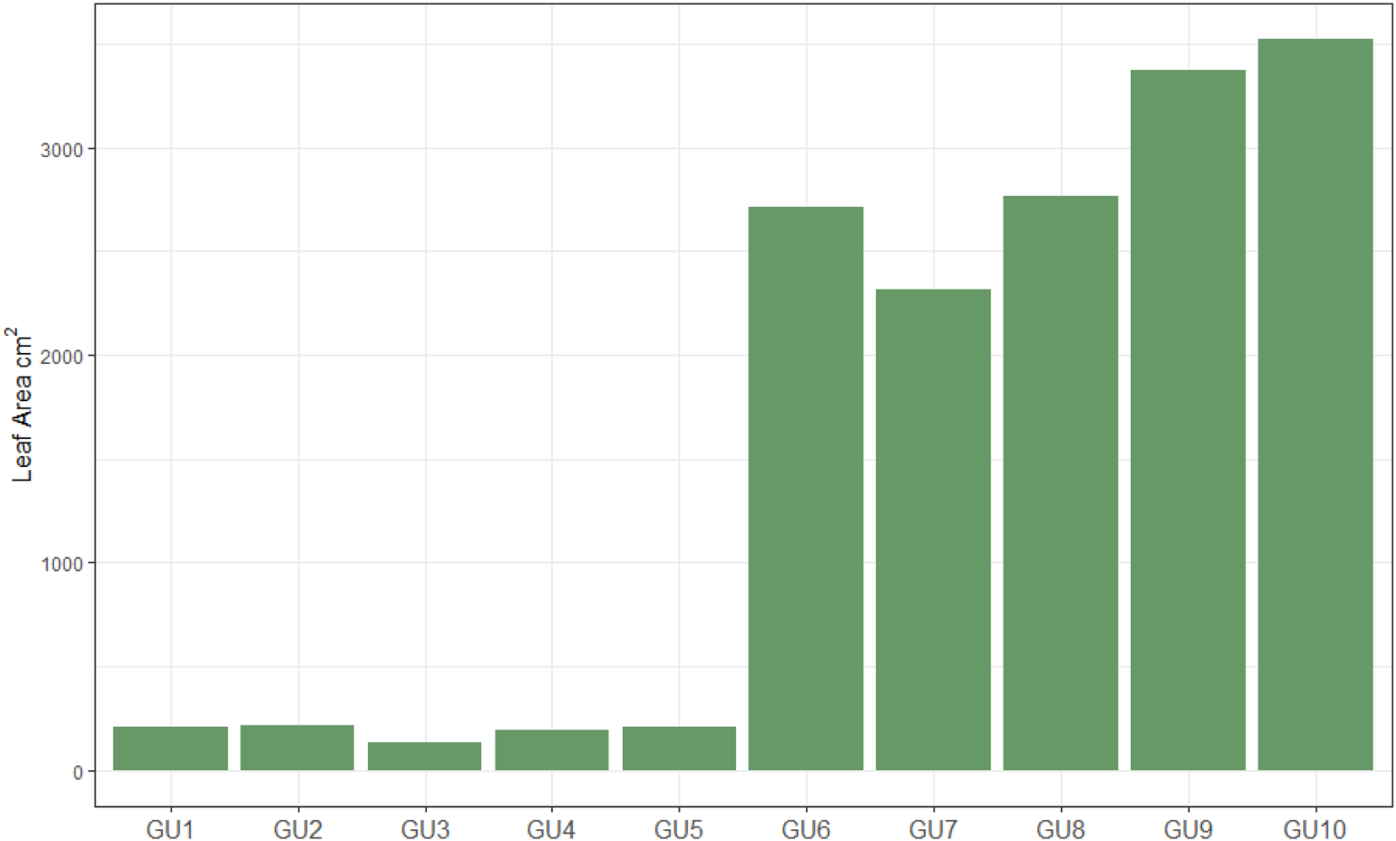
Total leaf surface area calculated based on measurements of every single leaf surface area per plant (evaluation of 3 consecutive days). GU1 to GU5 contain younger (PCS) plants (1 month after sowing), and GU6 to GU10 contain old plants close to physiological maturity (> 80 days after sowing; PCH)

Total leaf area (TLA) values were calculated for both experimental and control plants. The bar chart (Fig. 3) shows the calculated TLA per plant and nicely visualizes the growth differences between groups of smaller and big plants. The leaf area of smaller plants were around 200 cm^2^, meanwhile, the big plants’ leaf area was around 3000 cm^2^. Note, to standardize the row transpiration values among plants it is crucial to know the leaf area of each plant.

### Data analysis

**Fig. 4:**
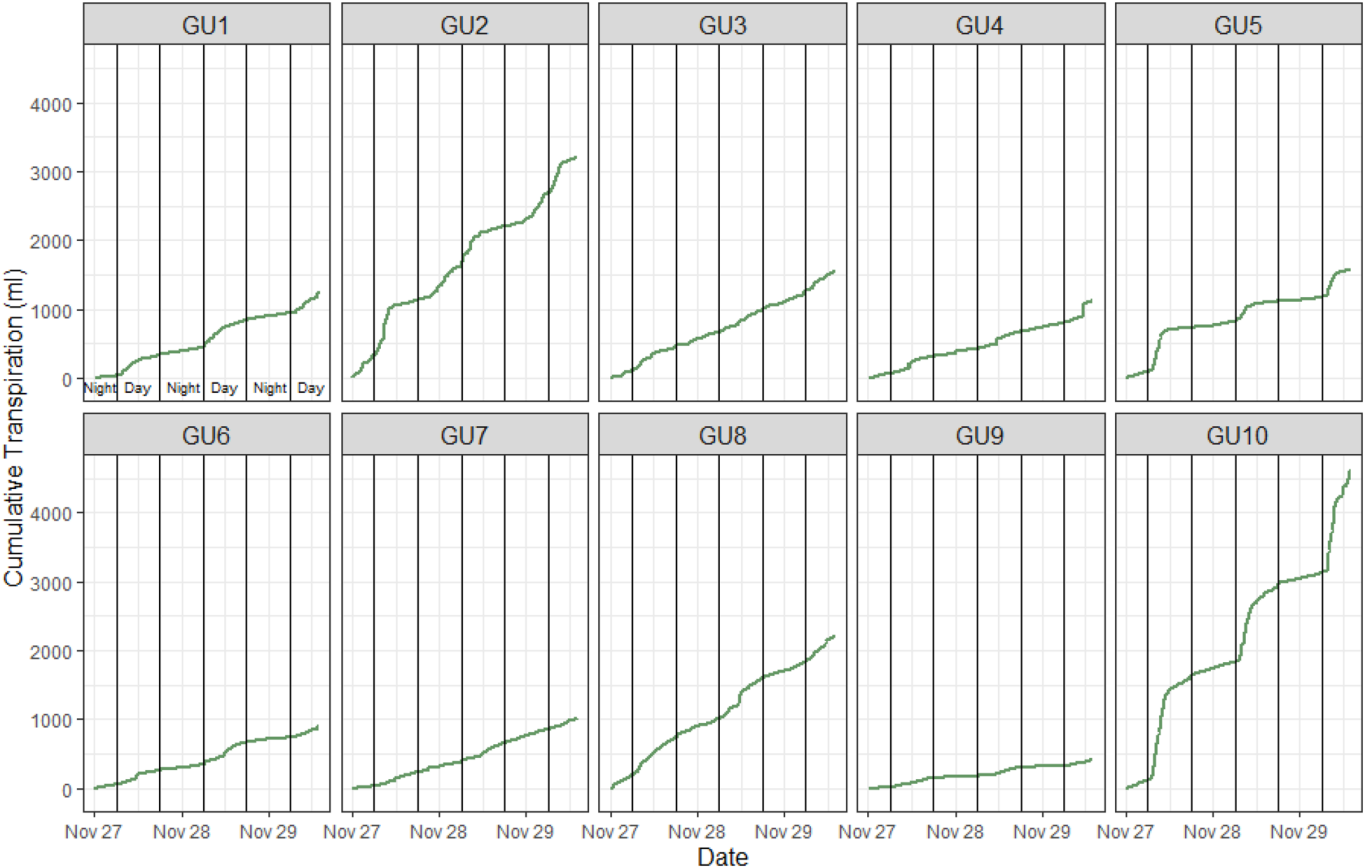
Cumulative transpiration was calculated for every single GU. GU1 to GU5 contain younger (PCS) plants (1 month after sowing), and GU6 to GU10 contain (PCH) old plants (> 80 days after sowing). The influence of the remaining noise from the load cell can be noted in GU2 and GU10 data

The cumulative transpiration per plant was calculated from GU data as the difference between the amount of water irrigated (ml) and the weight loss for 24 h (in our case for 3 consecutive days; Fig. 4).

**Fig. 5:**
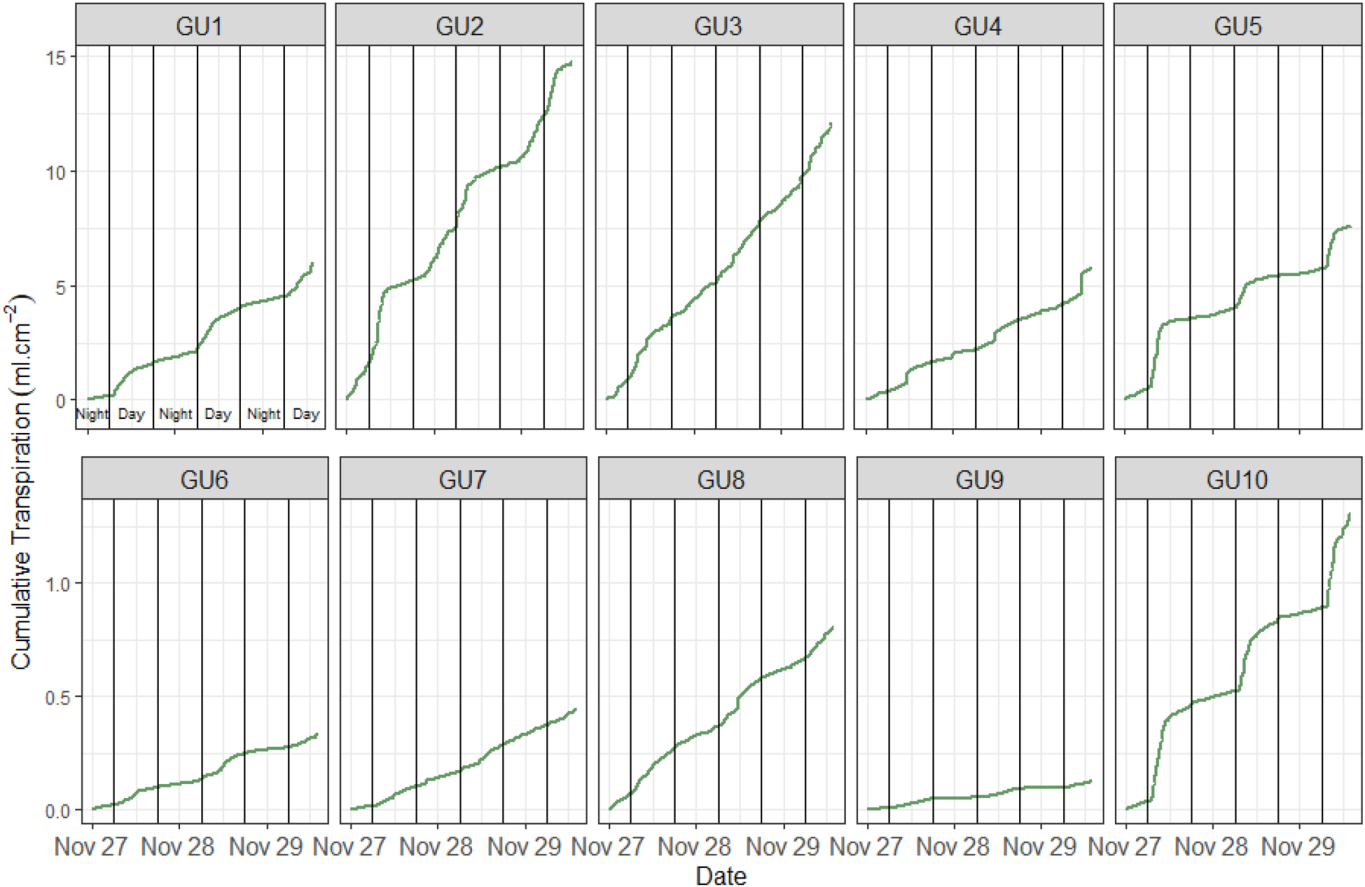
Net cumulative transpiration was calculated per leaf area unit for every single prototype. GU1 to GU5 contains data from young (PCS) plants (1 month after sowing), and GU6 to GU10 (PCH) contains data from physiologically mature plants (> 80 days after sowing). The influence of remaining noise can be noted in GU2 and GU10 data. Mark the difference in the scale between GU1-5 and GU6-10 due to TLA being lower on these plants

Additionally, from manually measured TLA values and cumulative transpiration previously obtained, the net transpiration standardized for LA was obtained for PCS (small; GU 1–5) and PCH (big; GU 6–10) plants (Fig. 5). Thus, the net cumulative transpiration per leaf unit (ml cm^−2^) by the plants in different phenological stages can be observed. Note the markable differences between day (high) and night (low) net transpiration (Fig. 4).

The results show the significant difference between cumulative values of plant transpiration standardized to leaf area (ml cm^−2^) regarding their developmental stage. Figure 5 shows the raw, however stable data of cumulative transpiration standardized per leaf area.

## Discussion

The LysipheN prototypes demonstrate their ability to measure 24/7 plant water consumption via transpiration (soil–plant–atmosphere continuum) changes according to the concept proposed by Poorter et al. [7]. The other variables such as stem and pods’ internal temperature, air temperature, air relative humidity, and soil water content were measured individually for a single plant. At the same time, the LysipheN units successfully supported plant growth without any supervision. This was achieved by controlling the daily irrigation of the individual plants based on plants’ previous water consumption data. The irrigation events were implemented by water injection reaching the defined target weight point every day (Fig. 1).

The 10 LysipheN units were assembled based on the final prototype constructed from different previous versions of the GU. In this study, the previous versions are not shown. However, the development prototype process can be found in Additional file 1: “Prototype development process” section. This process was oriented to solving issues related to irrigation systems, hardware selection, sensor stability, and reducing the noise affecting the load cells. Some of these issues were related to the electric noise or were caused by vibrations [17].

Monitoring water-use efficiency (WUE) in diurnal schemes or for the entire growth cycle under different environmental stresses (such as VPD or irradiance [24]) is extremely helpful for the detection of preferred phenotypes. The specific target population of environments (TPE) needs to be properly considered before data mining starts, as only TPE-related outputs are useful for breeders who depend on realistic contexts. Evaluation of the final prototype on plants focused on showing that the prototype can effectively measure transpiration water losses in real-time, precisely capturing the diurnal dynamics (Fig. 1). However, some prototypes (GU2, GU3, and GU10) showed noise susceptibilities in the weighing measurements (abrupt and non-logical changes in the chart) which affected data quality and the analysis. We kept these data for easier detection of transpiration variability by former users. This particular erroneous behavior can easily be repaired by acquiring a high-load cell-quality sensor. The prototypes revealed that PCH transpired 100–200 more grams of water than PCS plants per day (during the 3 consecutive days), under the same environment and soil conditions. This is an expected result since the TLA of PCH plants was over 2000 cm^2^ and PCS under 500 cm^2^ (Fig. 3). At this point, it is important to mention that plants’ transpiration under the same environment depends partly on genetics and partly on the presence of other abiotic or biotic stresses, and leaf temperature, thickness, and surface area [25, 26].

Knowing the weight losses per day, cumulative transpiration per leaf area/plant can be easily calculated using a particular formula (Fig. 4). As mentioned above, the transpiration of each plant should definitely be standardized per LA (Fig. 5) to unify responses between different plants and eliminate the genotype or treatment effect within each group. The calculations standardized to LA, allow us to determine that the amount of water transpired per leaf area was higher in smaller (PCS) than in bigger (PCH) plants due to the leaf phenology and in accordance with other physiological studies. These results demonstrated that the LysipheN prototypes are sensitive enough to detect and measure even tiny weight changes and then produce good transpiration data from very early to oldest plants across developmental stages.

With additional measurements (air RH, air, soil, and internal stem temperatures) the co-factor effects in varying VPD (Fig. 2) can be determined and support deeper analysis focused on factors that restrict transpiration. Genotypes with restricted transpiration could be more resistant to terminal drought but more susceptible to heat waves. However, the particular environment/soil scenario needs to be considered before any final decisions are made.

The developed GU prototype is an innovative low-cost tool for modern phenotyping. Our system showed that combining different technologies focused on plant measurements creates a synergy that meets specific needs of crop physiologists, pre-breeders, genebank curators, and breeders. Due to the previous calibration procedures, the LysipheN unit is completely portable, allowing it to work in different environmental conditions and be connected to different electrical sources (solar panels, any 110/220 AC source, batteries, 12 V power plants, etc.). Additionally, as an IoT phenotyping tool, each unit allows access to data remotely in real time through the web-user application. This application will send an email alert message if values are out of the programmed limits. Besides this, every prototype integrates an SQL database for data secure storing and algorithms able to calculate the water losses. The automatic adjustment (based on the predetermined stress scenario) of the next irrigation event based on the programmed watering scenario is another advantage of the system. High data precision will facilitate different crops’ growth such as rice, forages, bananas, and other species according to the researchers’ interest. Importantly, it will reduce the “preliminary experiments” to a minimum, especially when new stress, and more importantly new species are introduced. At the time of the study publication, the cost of materials per LysipheN unit (without manufacturing costs) was around 500 USD.

## Conclusions

The developed LysipheN prototypes could become an important low-cost tool for crop pre-breeding and phenotyping teams, especially in places where scientists have limited resources for a big phenotyping platform or where the number of studied materials varies or is low. Development efforts were focused on the assurance of data stability and quality and also on a user-friendly approach for researchers even in remote areas of the world. The full availability for remote use of these units is an intended and crucial advantage when region-specific responses are needed. In this case, soil, water, and other inputs are used from this particular place to ensure outcomes suitable for modelers, pre-breeders, and breeders. This process is called “target-specific breeding” and is in accordance with the recommendations presented by physiological breeding. So far, we built 10 LysipheN prototypes capable of indirectly recognizing the growth specifications of individual plants by direct evaluation of their transpiration with a high level of precision. Weight measurements can vary between prototypes; therefore, easy calibration and mainboard thermal corrections are sufficient to solve this problem. However, the load cell noise, water condensation, and vibrations can affect high-precision measurements when plants are very small (one or two true leaves) because daily weight changes are close to the load cell threshold (± 5 g). With bigger plants, prototypes demonstrated successful noise reduction and are very reliable. The next development efforts will be focused on implementing cleaning algorithms and using robust industrial sensors.

Using low-cost devices that allow remote connection to collect data in near real-time as also automatic irrigation based on feedback with previously transpired water is an important facilitation step for even small research groups to verify valuable hypotheses. The tool multiplies its benefits when selected functional traits are used in acceptable breeding contexts on materials with acceptable agronomic background (outstanding parental identification or early-materials evaluation). LysipheN is a tool to implement precise crop phenotyping, useful for breeders and physiologists but also in pre-breeding teams (characterization of genebank accessions by curators).

## Materials and methods

In order to detect errors and challenges and measure plant transpiration, from early to ultimate plant developmental stages the following methodology is outlined. All additional technical details are included in Additional file 1.

### Experimental design

**Fig. 6:**
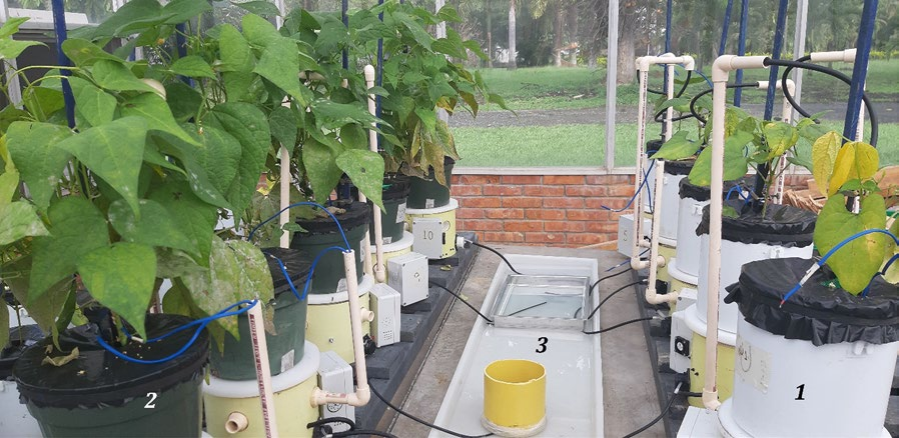
Prototypes with plants—two groups. 1. Right—5 prototypes with younger plants (PCS) (1 month after sowing). 2. Left—5 prototypes with older (PCH) plants (> 80 days after sowing). 3. Center—water source container for prototypes’ automatic daily irrigation supply

An experiment with one common bean variety was carried out with the 10 previously-calibrated GUs inside the greenhouse with one plant in each GU. The experiment proved system stability, and automatic irrigation scheme viability, and verified the minimum threshold in weight recognition (real cell-load accuracy). To assess the contrast in transpiration, plants evaluated using GUs were divided into two groups with 5 replicates each according to their phenological stage (Fig. 6). The first group was formed with plants 30 days after sowing (PCS) and the second group with plants closer to the final harvest (PCH) (> 80 days after sowing).

**Fig. 7:**
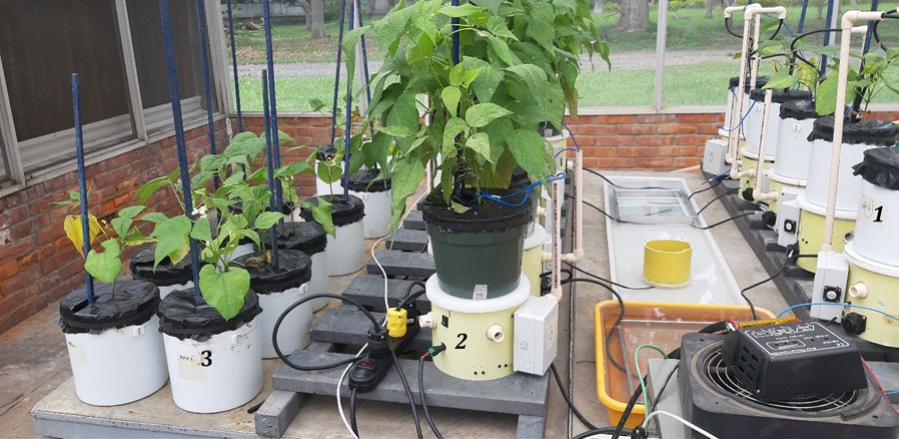
Prototypes with experimental (right) and control (left) plants. 1. Five prototypes with younger (PCS) plants (1 month after sowing). 2. Five prototypes with older (PCH) plants (> 80 days after sowing). 3. Control plants. Note that all pots are surface covered with black polyethylene. In the middle (between 1 and 2) there is an irrigation basin to feed peristaltic pumps for automatic irrigation

In addition, 15 extra pots with one plant per pot, were placed next to the prototypes for manual evapotranspiration monitoring (control plants, Fig. 7). The soil surface of both the control and experimental GU was covered with black polyethylene to eliminate evaporation by irradiation and so measure only transpiration changes of individual plants.

### Automatic data collection

Every GU prototype was scheduled to automatically supply water to the plant. The amount of irrigated water depends on the amount of weight lost via plant transpiration during the last 24 h, after the previous irrigation event. In this case, 100% of the transpired water was added to each GU for each irrigation event, and plants were irrigated with the same amount of consumed water. For the purposes of this study, 3 consecutive days were selected.

### Manual data collection

Some samples were taken manually during the experiment. Initially, 1 day before the experiment started, leaf length (LL) (from tip to petiole) and leaf width (LW) in millimeters at the leaves’ widest point were measured manually. However, these two values, LL and LW multiplied, give a rectangle area. Therefore, every single Leaf surface Area (LA) in cm^2^, was calculated by Eq. 1.

1 $$LA = \left( {LL \times LW} \right) \times GF.$$

Note that GF is a genotype factor, which is a constant value of the area inside the rectangle that is not part of the leaf. This GF factor was obtained previously, and can be specific per used genotype.

Thus, the Total Leaf surface Area in cm^2^ (TLA) was calculated per plant (Eq. 2) as the addition of every single Leaf surface Area (LA).

2 $$TLA = \mathop \sum \limits_{i = 1}^{i = n} LA.$$

TLA values were calculated for both experimental and control plants. Note that in Eq. 2 “n” is the number of trifoliate leaves in the plant, and LA is the single leaf area. Additionally, the number of leaves was also obtained. TLA measurements are depicted in Fig. 3 (Results section).

### The LysipheN prototype

The LysipheN prototype is a mechatronic device, including hardware and software implemented in a mechanical structure. The mechanical structure is constructed as a robust, compact, and light assembly allowing the LysipheN to be easily portable. For the hardware implementation, the electromechanical devices, a microcontroller, IoT devices, and other sensors were equipped and plugged into an embedded electronics system. The software was developed using different programming languages and communication protocols.

The LysipheN prototypes can measure different types of variables in the soil–plant–atmosphere continuum. The directly measured variable is transpiration, calculated via system weight changes over time. Other sensors (the tissue temperature, soil moisture, relative air humidity, and air temperature) serve as a source of additional data and support the high reliability/repeatability of the experiments. Using a peristaltic pump, the prototype can perform automatic and precise irrigation during the whole plant life cycle. Each GU is able to accurately quantify the transpiration losses and automatically adjust the later irrigation amount together with feedback from individual pre-settled unit treatment (irrigation amount in ml can vary from 0 to > 100% of the water loss during the evaluated period of time, based on the realistic or hypothetical stress scenario). The LysipheN-cultivated plants grow without any supervision and their performance is much better than in hand-irrigated plants (no damage by errors in over-watering by technicians). The initial parameter setting thus serves to supply plants with adequate water availability during the entire cycle or support constant under or overwatering.

Accessing data in real-time is possible since every prototype has integrated a remote-access web application. LysipheN prototype portability allows users to easily move the tool to the evaluation site. The right combination of genotypes (deep understanding of parental lines for further crossing) together with the selection upon different stressors (climate, soil, watering regime, response to fertilizers, rhizobia etc.) will help to obtain proofs of concept for physiological trait/allele combinations that could boost yield, but still within a realistic breeding context.

#### Mechanical structure

**Fig. 8:**
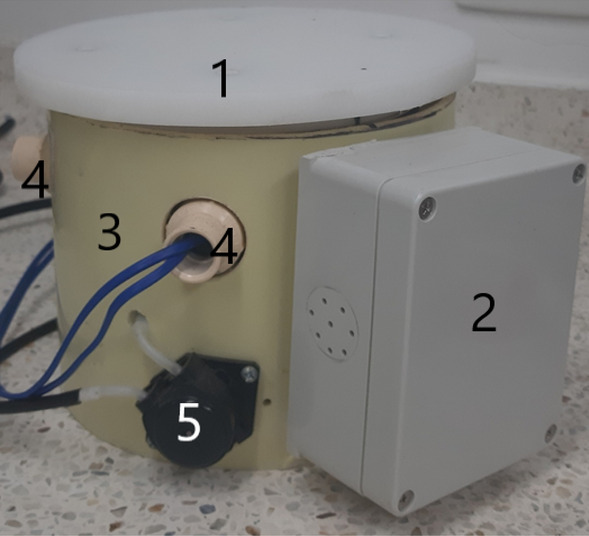
Gravimetric unit. 1. Teflon base for placing the pot. 2. Electrical Ip65 box. 3. Circular soft yellow 6-inch diameter PVC base. 4. Output holes for connecting sensors and dripping irrigation tubes. 5. Peristaltic pump for irrigation

The structure was built (Fig. 8) using elements purchased in a home store (our case in Homecenter in Colombia). Construction elements of the prototypes consist of commonly used materials such as polyvinyl chloride tube (PVC) 6-inch length and tube ½-inch diameter, polytetrafluoroethylene (PTFE; Teflon) 6-inch diameter, and electrical square box 12 × 12 cm. The mechanical structure contains an integrated weighing sensor (Fig. 10), but also, an irrigation system (actuator), cable and water hose protection, and an electronic box. The GU prototype structure itself (depending on the load cell capacity) is strong enough to support the weight of the complete system (pot, soil, and plant) up to 60 kg.

### Hardware

**Fig. 9:**
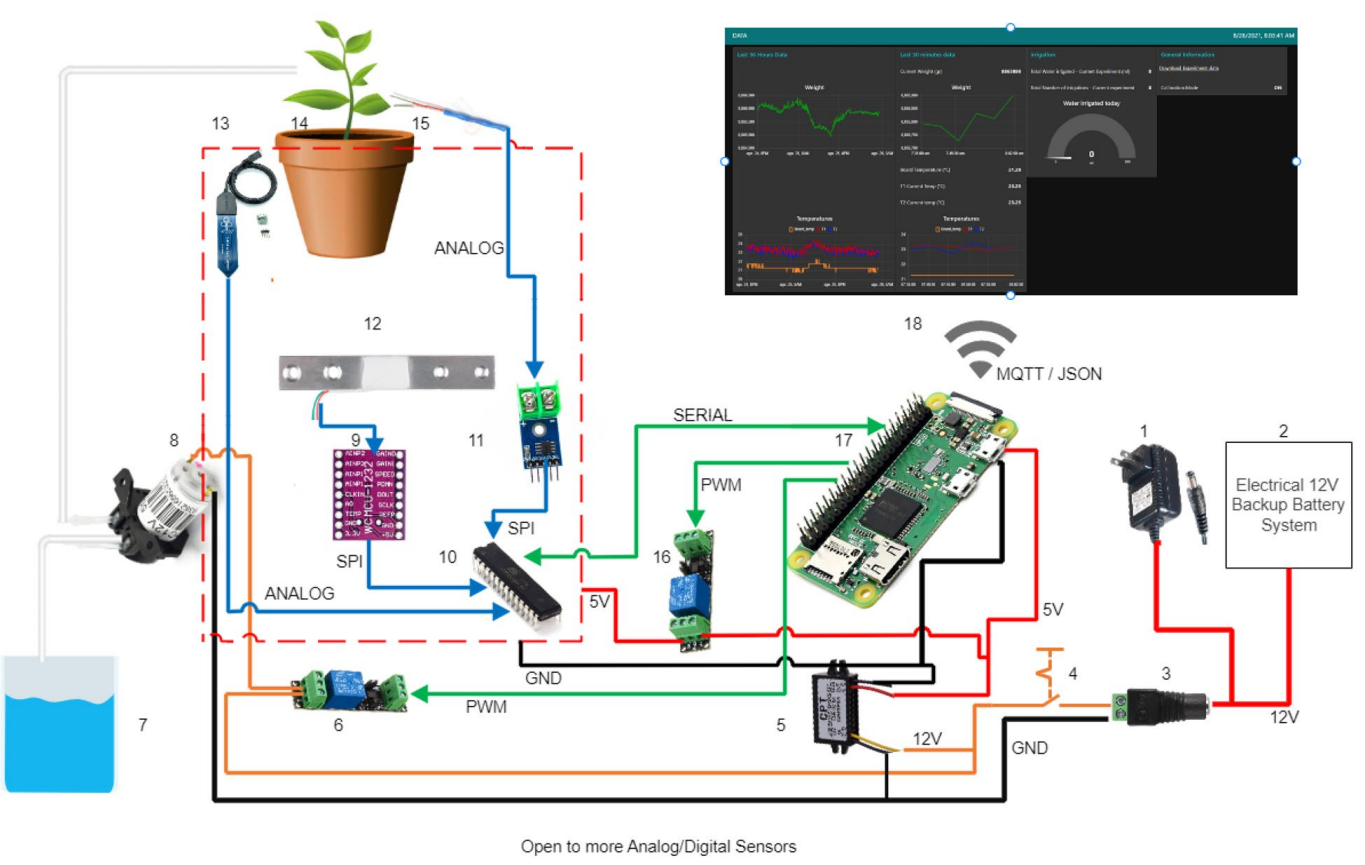
Hardware scheme connection. 1. 120 V–12 V DC adapter (electrical source). 2. Electrical 12 V backup system. 3. Female connector. 4. Switch. 5. 12–5 VDC converter. 6. Relay for controlling the irrigating pump. 7. Irrigation water source (tank). 8. Peristaltic pump for irrigation. 9. ADS1232 ADC. 10. Atmega328p microcontroller. 11. MAX6675 ADC. 12. Load cell (weight sensor). 13. Analog soil moisture sensor. 14. Plant in a pot. 15. Tiny k-type thermocouple (temperature sensor). 16. Relay for electrical on/off switching of the sensor electronical system. 17. Raspberry Pi zero w. 18. Web application (user interface)

Since the GU prototype was developed as a complete embedded IoT development system, the hardware required a design adaptation process to build a compact electronic board with ports for the sensors, and other devices that facilitate the electronic working of the prototypes. This adaptation and electronic design process were carried out in different stages oriented to the GU hardware to be fully competent for the various parts: sensors’ data acquisition; irrigation control; electrical source connection and backup; data collection; control microcontroller, and IoT internal communication and application’s device (Fig. 9.).

Once the mechanical structure was ready, the physical scope was defined to retain the ergonomics and protection of the electronic system without neglecting its basic operation.

#### Sensor’s data acquisition

##### Weight system measurements

**Fig. 10:**
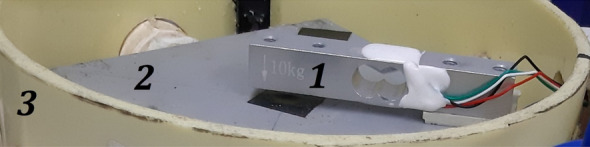
Weight sensor installation. 1. Weight sensor (load cell). 2. Metal box as a support. 3. Circular soft yellow 6-inch diameter PVC base of the whole unit

The weighing system (sensor, amplifier, and converter) returns the primary and most important variable in the measurements. A load cell [27] was used as the sensor to measure raw data in terms of voltage changes depending on the compression applied to the sensor (Fig. 10). Thus, after signal treatment, the system’s weight changes are measured for every required sample time (from milliseconds to hours), and—logically—individually for each GU. Therefore, considering a known initial dry weight of the substrate (soil), the weight system calculates the actual soil water content in % and according to how much water was supplied/lost evaluates the pot humidity in each period [28]. The sensor junction with the body of LysipheN was easily assembled in the structure using screws (Fig. 10).

As the load cell works at a very low voltage level with 3–5 V input (1 mV in this case as an output signal) it is necessary to use the signal amplifier system (instrumentation amplifier) [28]. The ADS1232 analog-to-digital converter (ADC) was used for this purpose (Texas Instruments, USA).

##### Internal pod and stem temperature measurements

The prototype includes two thermocouple sensors to measure the temperature inside the stem and the fruit/pod. developed a methodology to measure the sap flow (however, this concept needs to be verified in our system as the most important part is the sensor insulation). In our case, the two sensors measure the internal pod and internal stem temperature by using the K-type thermocouple (K-type Teflon thermocouple with Nickel chromium and Nickel aluminum alloy) (Uxcell K-Type Thermocouple Wire). Each thermocouple (sensor) has an adapted welded end and the temperature changes applied to this union generate a small voltage as an output (mV) (Seebeck effect) transformed into the temperature.

This data provides essential insights for plant modelers. Future crop models must accurately use varied tissue temperatures to estimate air temperature’s impact on growth in a changing climate. During this phase, internal stem and pod temperatures were measured to find links to other variables, plant behavior, and the influence of direct sun radiation.

##### Electronical board’s temperature measurement

There is a recognized temperature-based influence on electronic devices during their operation that could directly affect the reliability of working [29]. Therefore, methods to reduce the effect of the temperature should be applied in the design of electronic embedded boards [17] For this purpose, the temperature sensor TMP36 [30] was used to measure the air temperature related to the microclimate to which the electronical board is exposed. The data from this sensor is used to run an auto-compensation temperature calibration algorithm for more precise weight measurements.

##### Environmental variables

The monitoring of the immediate/cumulative plant responses according to treatment concerning actual climatic conditions is a necessary step for climate change-oriented plant research [18]. Besides, these variables are extremely important in any comparison of plants’ responses [31]. However, sometimes climate data from far-set weather stations are used. Even if the climate is measured in plant proximity, some basic errors can easily occur because of convection, conduction, or radiation. In the developed LysipheN prototype, air temperature (T_a_) and relative air humidity (air RH) close to the GU location are measured to know the dynamics of these variables during the experiment. These variables (T_a_ and RH) play a crucial role in terms of the VPD calculation in relation to these and transpiration demand [32]. Thus, based on that data, the evapotranspiration models can be calculated [33].

To measure T_a_ and air RH, an SI7021 IoT sensor together with a Sonoff module was used. The sensor measurement range is 0–100% RH, and − 10 to 85 °C. The precision of the sensors is approximately ± 3% in humidity and ± 0.4 °C for the temperature measurements. This sensor does not require a calibration process. Only the Tasmota code [34] must be uploaded to the Sonoff module with the Wi-Fi credentials. This module is very small. Therefore, plant development is not affected.

#### Water irrigation control

Poorter et al. [7] proposed different options to evaluate water consumption in the sense of irrigating plants in each pot individually. The consumed water can be replaced by plant irrigation while maintaining a nearly constant soil water status during the day [21]. Irrigation was supplied with high precision actuators (water volume error 5–10 ml) which were also used to support two different irrigation systems (dripping point from above the soil surface and classic bottom irrigation reaching first the roots). Having both systems available will allow a wider range of plant types to grow, including lowland rice under simulated flooding [21].

The developed LysipheN prototype includes a programming algorithm for, among others, consulting local network time every 10 s in cases (experiments) where irrigation control by a specific time is required (below). The data of actual transpiration or tissue (leaf, bud, flower, pod) temperature are immediately available after any water supply.

For the precise control of the irrigation system, a 12 V peristaltic pump [35] was used and connected to a digital relay which is an electronic device used to implement switching on/off logic [36]. During each experiment, irrigation process parameters may easily be changed depending on the user or plant requirements according to the type of water availability scenario in the developed user interface (UI) for the prototype (below). In a factory setting, irrigation was scheduled every day at a specific programmed time (usually early morning).

#### Electrical source connection and backup

Any experiment with LysipheN units needs an autonomous electrical and commonly used universal 12 V source. This allows its use at any location or country. Therefore, all experiments involved a battery backup system, which in parallel, secured 100% of the time working for the ten replicas built.

#### Data collection and control microcontroller

For an electronic system that requires sensor use, it is essential to have a microcontroller capable of reading and converting analog signals or digital messages into useful data. To solve this problem, the Atmega328p was selected [37].

This microcontroller programming is open source (using C++ language) and can be done using the Arduino IDE.

However, to get remote access and an internet connection for the prototype, this microcontroller needs to be accompanied by another device capable of saving data and sending it via Wi-Fi (as the Atmega328p does not have these functions).

#### IoT internal communication and application device

As mentioned above, Atmega328p (microcontroller) is used only for sensors data readings. For the prototype, it was necessary to use another device serving as a complement to this microcontroller able to both save and send data via Wi-Fi and also show them in an application Node-Red interface (see Raspberry application’s user interface programming). To solve this, we selected Raspberry Pi Zero W [38]. It is a small development board, a one-core processor with 512 MB RAM, and integrated with a 32 GB microSD card for storing data and OS working.

The Raspberry Pi Zero W function is highly important in the prototype’s working. It allows, among others:

- Host the main LysipheN unit web application (Fig. 15) where all collected data are shown and plotted in real-time.
- Store data in an internal integrated MySQL database.
- Control the peristaltic pump.
- Communicate and request data from Atmega 328p Microcontroller.
- Make easy wireless microcontrollers and main web application reprogramming.

The use of this development board is like having a minicomputer within each LysipheN. Because it will allow easy connection and configuration of the main web application as well as allow integration of possible behavioral algorithm estimation and correlations that are within the limits of what is permitted by Raspberry’s characteristics.

#### Main electronic board design

**Fig. 11:**
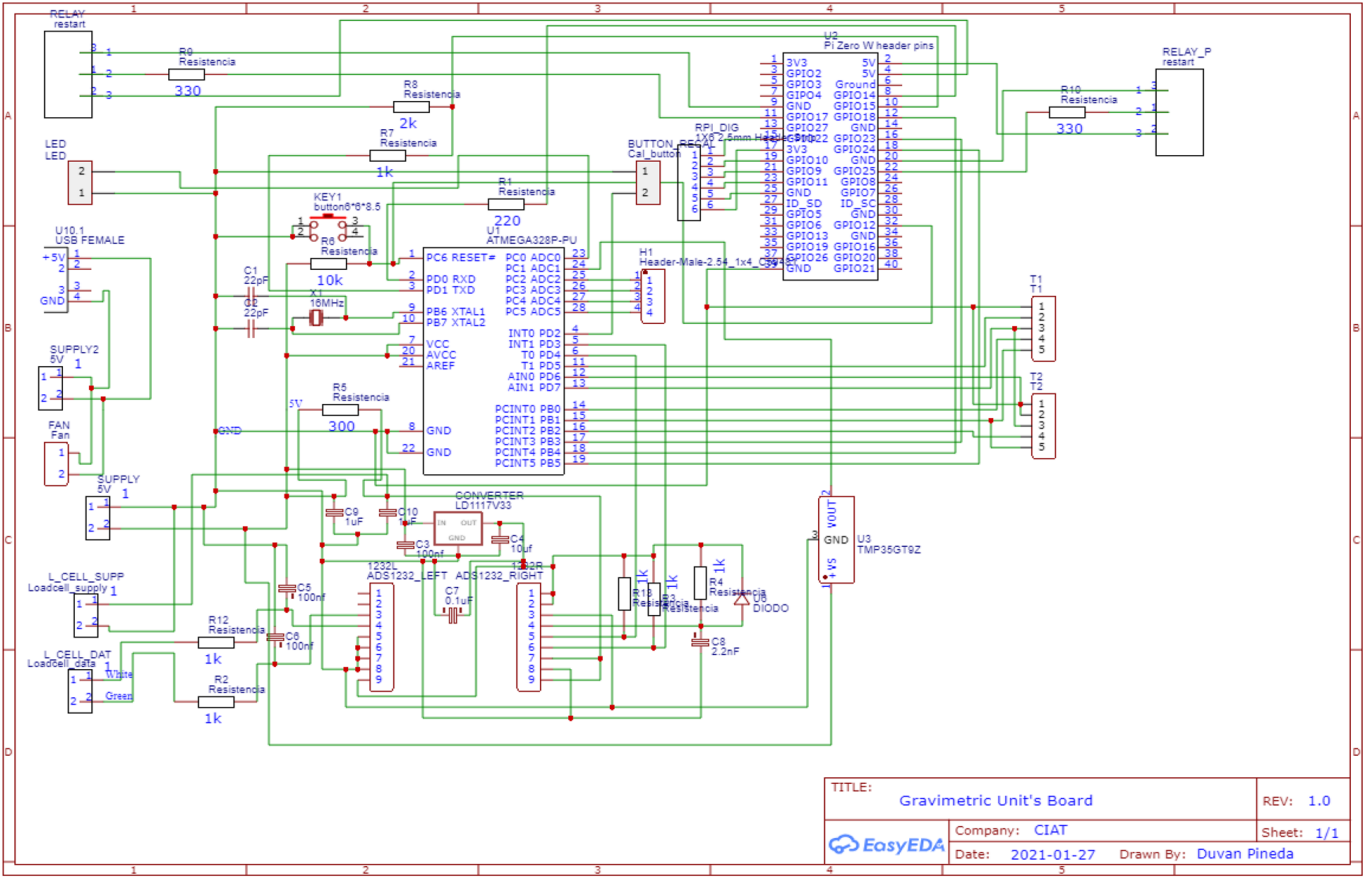
Schematic design in EASYEDA

**Fig. 12:**
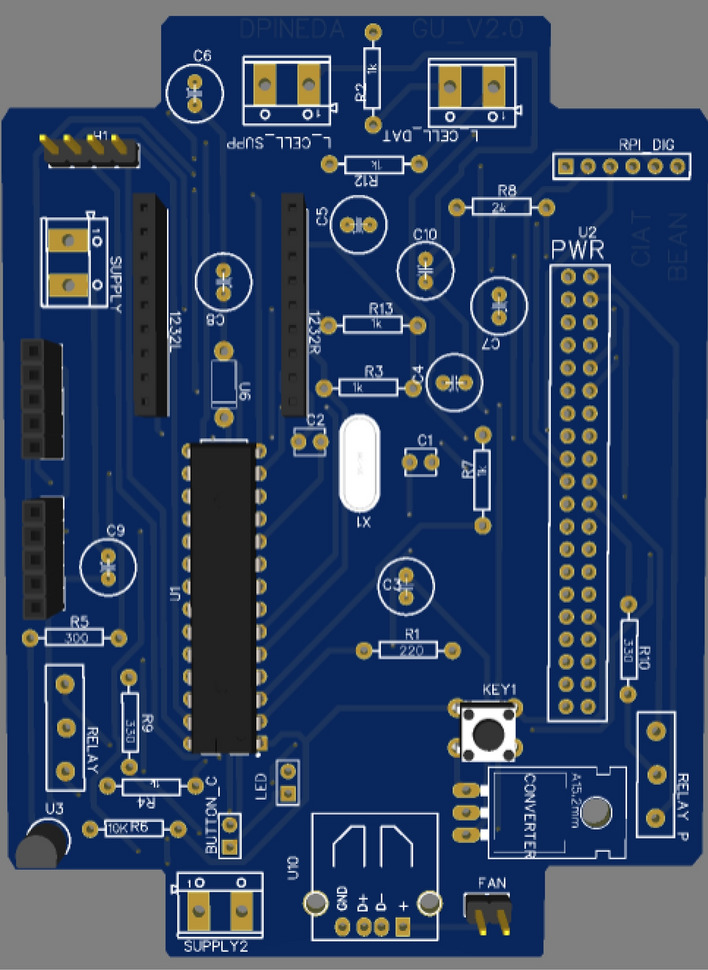
PCB design (top view), JLCPCB preview

The developed prototype has an integrated electronic board that contains ports for all devices necessary for the system’s working. For the correct integration, the development of the board carried out separate tests per device into an embedded system (Figs. 11 and 12). Thus, each sensor was tested individually using Arduino Uno to verify the communication working and data consistency.

The electronic board is equipped with all devices and connections required. It is divided into three different parts.

The first part is composed by:

- Supply connections that are connected to the 12 V main source.
- Internal 5 V and 3 V converter that will supply to the Microcontroller, Raspberry, and other devices.

The second part is accomplished by:

- Microcontroller (Atmega328p).
- Raspberry Pi Zero W with working respective connection.

The third part in the main electronic board was meant for sensors connection.

- Weighing system (load cell, electric filter, and ADS1232 ADC) using a wired connection to the Atmega328p microprocessor.
- Two K-type thermocouples (tiny Teflon end welded).
- TMP36 sensor.
- Relay for ON/OFF peristaltic pump control.
- 12 V peristaltic pump for precise controlling of water supply.

Finally, each unit has a main button, to start the entire system when it is switched off or to do a reboot, if necessary. A mini 5 V fan was connected to the IP65 box to cool it and make some airflow over electronic devices inside the box.

#### Server web hosting device

The GU project now comprises 10 prototypes. Even though each GU is equipped with a Raspberry Pi Zero W that works, among others, as application web hosting, this application is focused on single prototype information. Therefore, a Udoo Neo [39] model has been used as a server where the Node-Red general application works. This application contains data information of each GU connected to the same network and it is part of a bean phenotyping platform (in preparation) developed with MySQL and other saving and processing tools (below).

### Software

The prototypes’ Programming routine was built in two different languages and programming environments. The microcontroller was programmed in C++ through Arduino IDE uses, and Raspberry Pi’s application was developed on Node-Red, which is a programming tool based on visual connections of nodes and allows different uses of internal Raspberry input and output pins, besides Wi-Fi connection and MQTT communication.

#### Communication

**Fig. 13:**
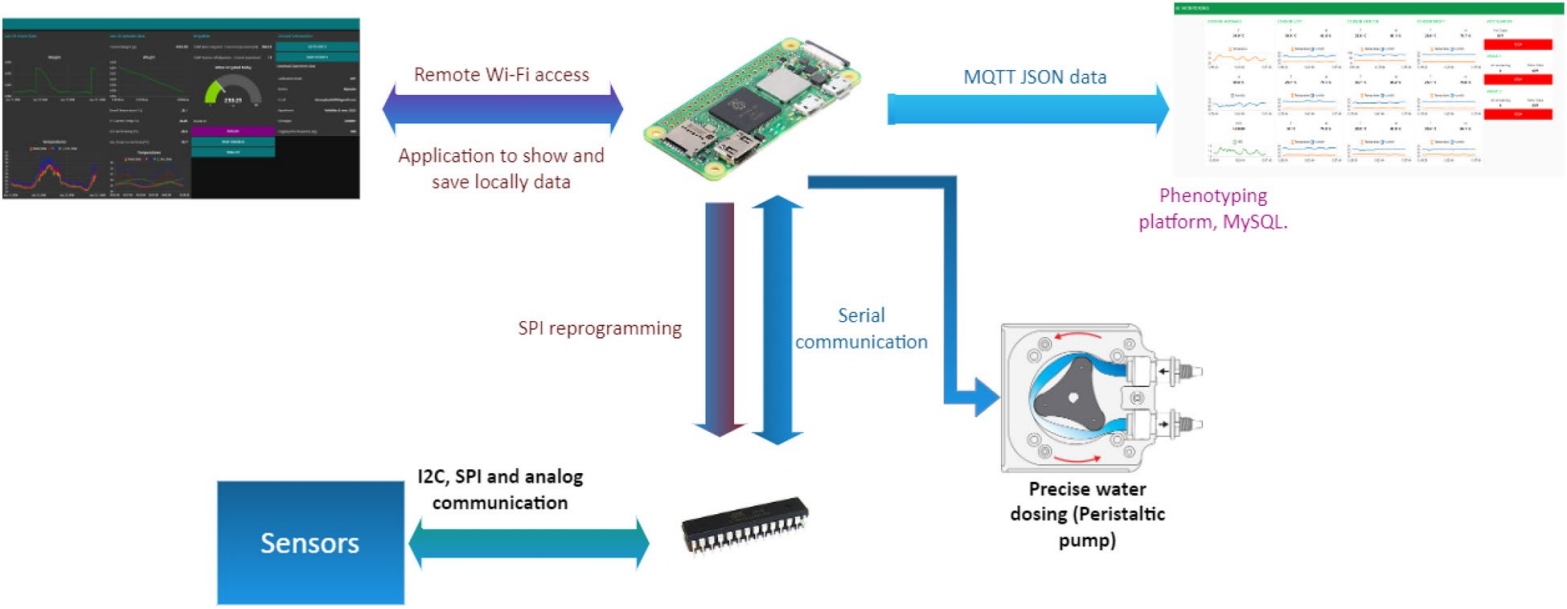
Prototype communication flow

Perfect communication synchrony between devices is critical for any automated IoT development. Establishing the protocols and making sure that the data will not be lost, must be considered during system routine construction (Fig. 13). In our case, the Raspberry Pi and Microcontroller are compatible with serial communication, which makes it easier to share data processes between them. These two devices are perfectly synchronized. The Raspberry Pi Zero W sends a JSON package to the Phenotyping Platform with the data of each prototype using the MQTT protocol at each interval time previously defined.

Another important aspect of how to increase the communication quality for each LysipheN unit was the direct SPI connection between Raspberry Pi Zero W and the microcontroller. This allows the user to change the programming routine in both devices at any time and gives the possibility to add or change sensors and actuators in the future.

#### Microcontroller programming routine

**Fig. 14:**
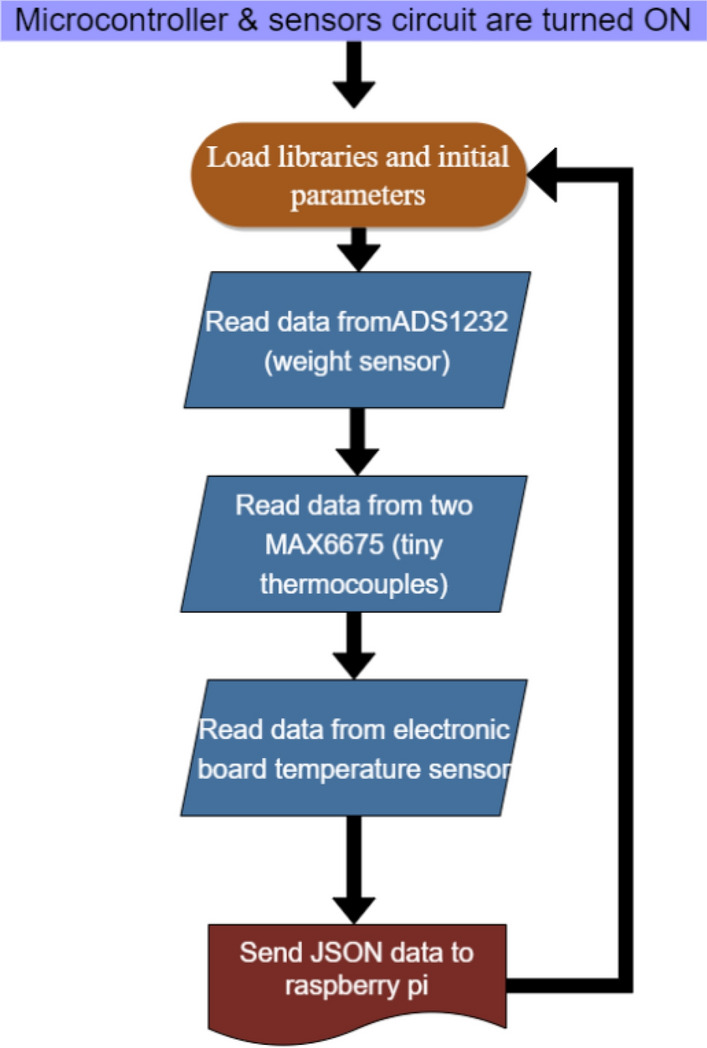
Microcontroller programming routine

A working program was written in C++ language to establish the main routine of the device that will collect data from all sensors (Fig. 14). This program code considers the working requirements of each sensor.

First, the program will load whole libraries and initial parameters to read the traits of the sensors’ signal data. Then, the user can choose in the Raspberry Pi Zero W application (see “Raspberry application’s user interface programming”) whether the weight temperature calibration will begin or not. If yes, this process will take 1 or 2 days depending on the required quality of the calibration. If not, the program will assign the parameters obtained during the last calibration to the weighing process which can then start immediately.

If a data request arrives from Raspberry Pi Zero W, all sensors’ data are read and packaged in a JSON message, and it is sent to Pi Zero. Then, Atmega328p and the sensors are electrically disconnected for a previously defined time.

#### Raspberry application’s user interface programming

**Fig. 15:**
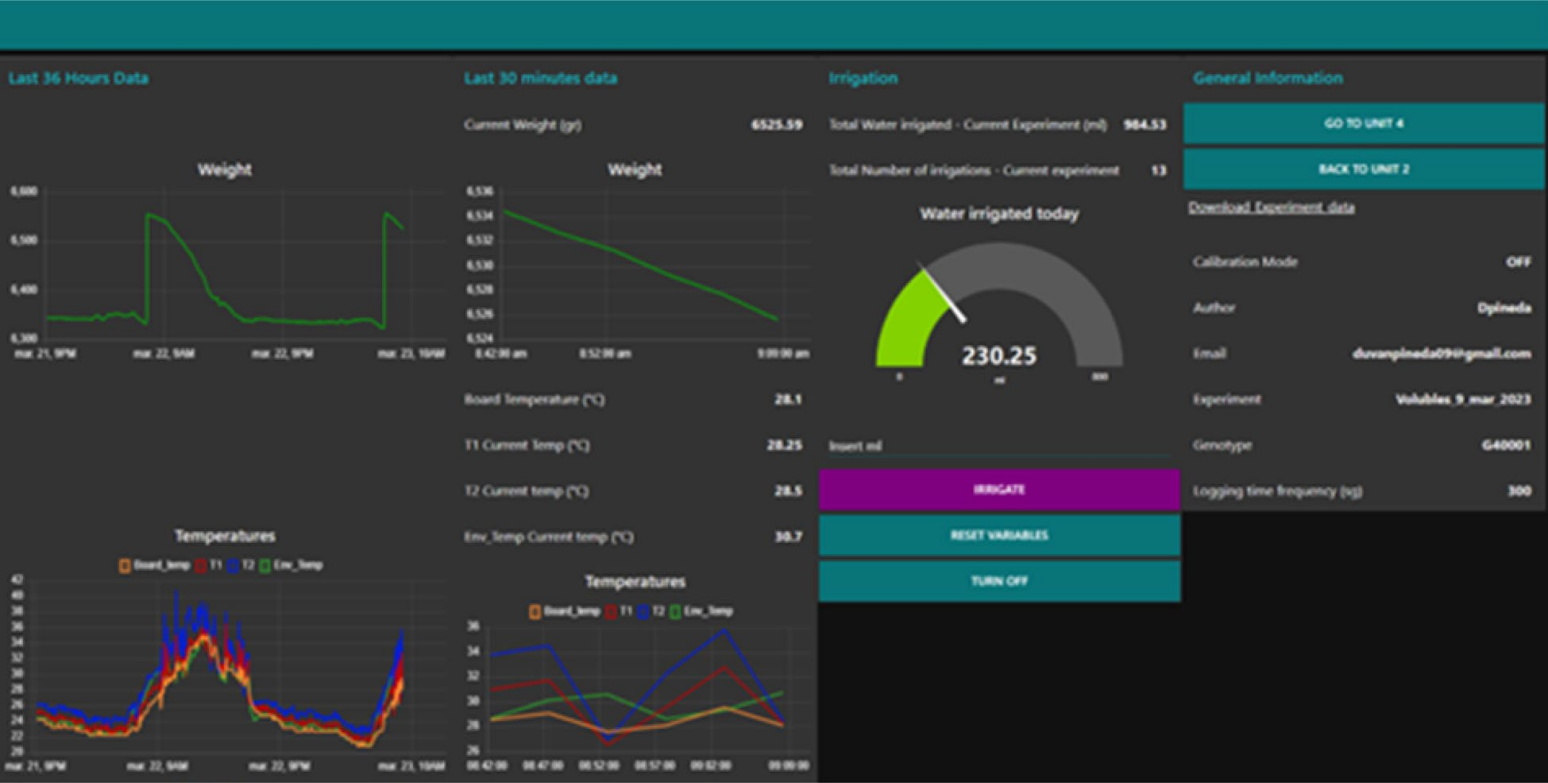
Application interface with real-time data during the calibration process. Left to right; the last 36 h the weight and temperatures plot; the last 30 min weight and temperature plot; total water; and the current amount irrigated in the experiment and downloading data panel

Every single unit was equipped with its user web application (Fig. 15). The role of the Raspberry Pi Zero W is to run the main application that uses a Node-Red programming environment, which is based on visual Programming through node connection and allows writing functions, Raspberry’s GPIO uses, and communication protocols such as MQTT. The flowchart is the name of the routine programmed, and it begins a short time before Raspberry starts.

**Fig. 16:**
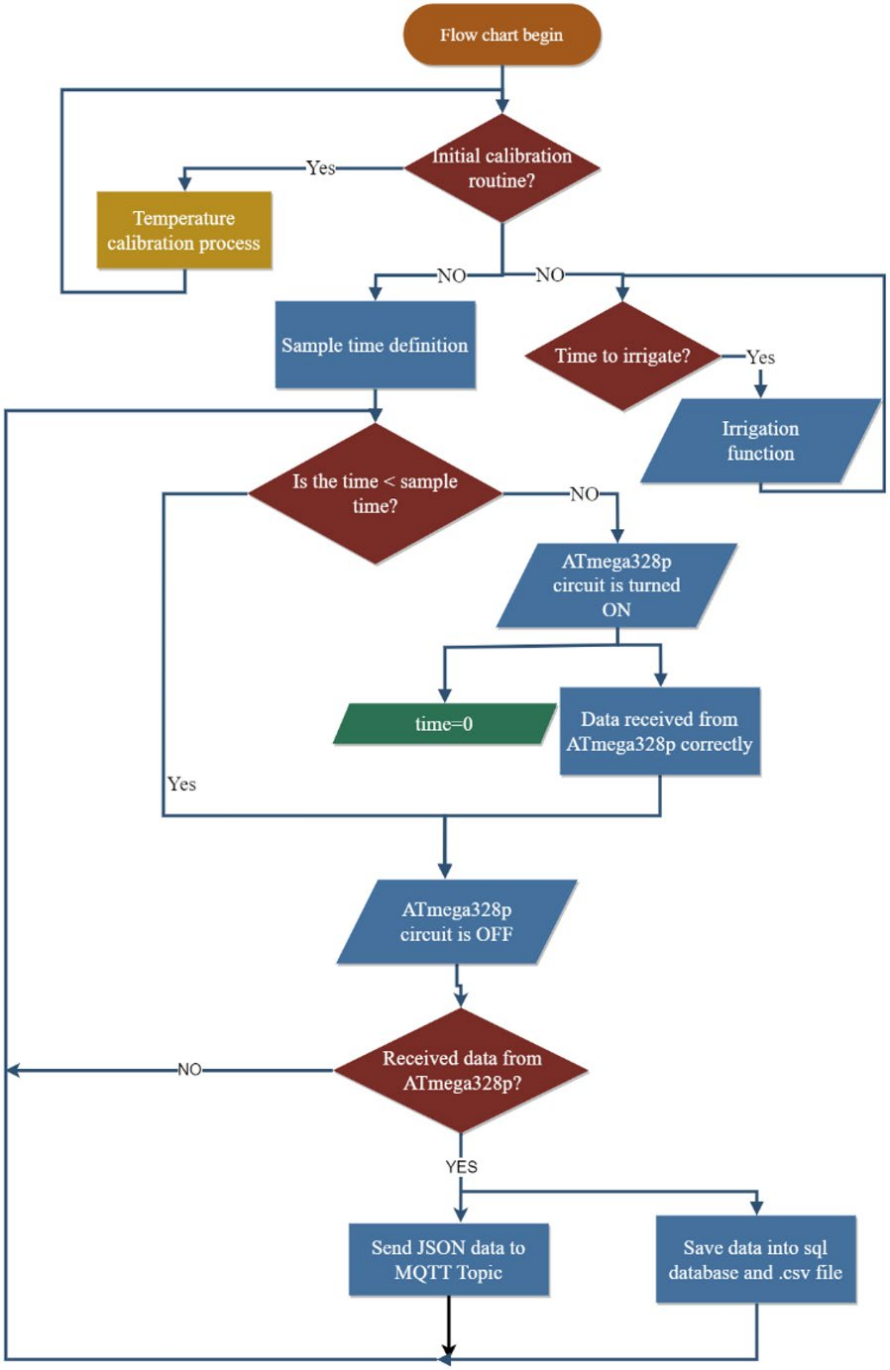
Individual prototype programming of the user application

The application manages:

- Correcting the microcontroller’s electrical connection/disconnection.
- Requesting, receiving, and storing data from the microcontroller.
- Watering for daily irrigation control.
- Plotting the real-time weight and temperature changes in a web interface (Fig. 15).
- Sending data to the Phenotyping platform (Fig. 16).

Another important feature related to routine programming works in “parallel.” This enables the applications to be pending the defined irrigation time and simultaneously request, save, and send the data. Thus, when the time of irrigation is reached, the application executes the function that supplies the water based on the parameters defined previously.

All received data are stored automatically in the MySQL database. Then, the database updates a CSV file with the new information and this file is ready to be downloaded at any time as users require. At the same time, a new JSON package is created and sent using the MQTT protocol to the Mosquitto broker (phenotyping platform server), where data from the 10 devices were saved into the MySQL database and interfaced in the phenotyping application in real-time. Once the data are received and saved, the microcontroller state is changed to OFF again and the cycle is restarted depending on the sample time defined by the user previously. This not only saves energy and protects the system, but also helps to keep the load cell in the correct calibration stage.

Once the sampling time is reached, the Atmega328p state is switched to ON and then, the application requests data using the serial path.

#### Phenotyping platform

**Fig. 17:**
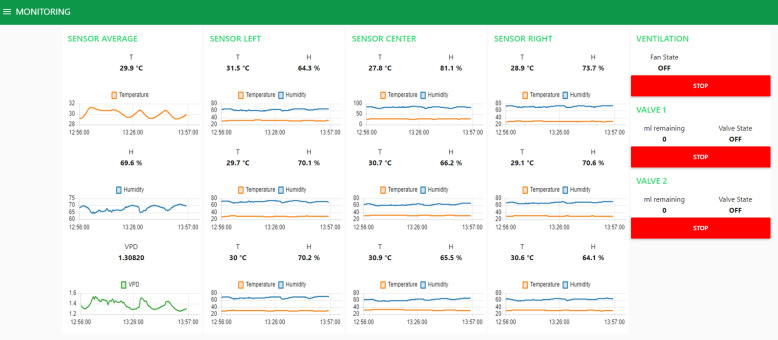
Phenotyping platform. Note, that it is possible to access to complete experiment’s location-measured data and controlled variables information together with prototypes’ received data in a global database

The aim of the platform was to construct an IoT-based automation platform to monitor and control environmental conditions inside greenhouses [40]. This platform includes vast information from many experiments on beans (*Phaseolus vulgaris* L.) at CIAT. It was developed as an open service able to receive, store, and show data in real-time. In addition, the IoT platform includes the application developed for requesting, receiving, and storing data from the Gravimetric Units (ten replicas), which is a necessary complement for the phenotyping development described in this study (Fig. 17).

The phenotyping platform allows a general view of the data from the LysipheN unit prototypes in a real-time. In addition, this tool is able to integrate physiological and statistical analysis useful for breeders or other research purposes.

It is important to mention that communication between the Phenotyping Platform and the LysipheN is made through the internet connection since both (the prototype and phenotyping platform) manage the wireless connection. However, developed LysipheN prototypes can be used without the internet, even though Wi-Fi is only for real-time monitoring and initial configurations, all the previously specified prototypes’ functionalities were programmed to work with only an electrical source.

**Fig. 18:**
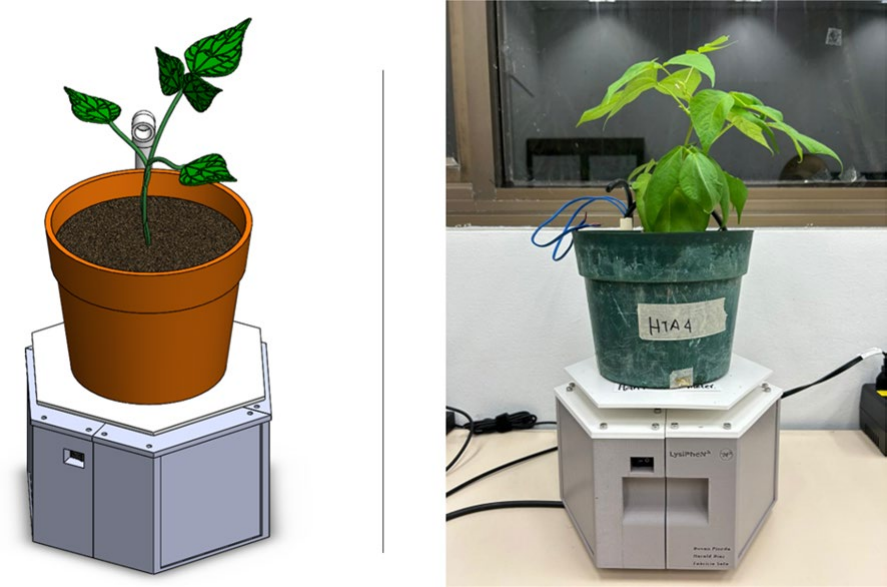
3D printed prototype (the newest version)

A newest version of the hexagonal base of LysipheN printed in 3D (Fig. 18) to support multi-crop usage in different environments.

### Supplementary Information

**Additional file 1.** Development process, tests, results and issues from the previous prototype versions of LysipheN.

## Data Availability

The datasets used and/or analyzed during the current study are available from the corresponding author on reasonable request.

## Abbreviations

ADC: Analog to digital converter
EUW: Effective use of water
ET: Evapotranspiration
GU: Gravimetric unit
PCS: Plants closer to sowing
TPEs: Target population of environments
TE: Transpiration efficiency
VPD: Vapor pressure deficit

## References

[1] Mayor-Duran VM, Raatz B, Blair MW (2016). Desarrollo de líneas de frijol (*Phaseolus vulgaris* L.) tolerante a sequía a partir de cruces inter acervo con genotipos procedentes de diferentes orígenes (Mesoamericano y Andino). Acta Agron.

[2] Vadez V (2014). Root hydraulics: the forgotten side of roots in drought adaptation. Field Crops Res.

[3] Ribeiro T, da Silva DA, Esteves JADF, Azevedo CVG, Gonçalves JGR, Carbonell SAM (2019). Evaluation of common bean genotypes for drought tolerance. Bragantia.

[4] Kholová J, Urban MO, Cock J, Arcos J, Arnaud E, Aytekin D (2021). In pursuit of a better world: crop improvement and the CGIAR. J Exp Bot.

[5] Jump AS, Peñuelas J (2005). Running to stand still: adaptation and the response of plants to rapid climate change. Ecol Lett.

[6] Darkwa K, Ambachew D, Mohammed H, Asfaw A, Blair MW (2016). Evaluation of common bean (*Phaseolus vulgaris* L.) genotypes for drought stress adaptation in Ethiopia. Crop J.

[7] Poorter H, Fiorani F, Stitt M, Schurr U, Finck A, Gibon Y (2012). The art of growing plants for experimental purposes: a practical guide for the plant biologist. Funct Plant Biol.

[8] Amede T, Kimani PM, Rono W, Lunze L, Mbikayi NT. Coping with drought: strategies to improve genetic adaptation of cornrnon bean to drought-prone regions of Africa. 2004.

[9] Vadez V, Kholova J, Medina S, Kakkera A, Anderberg H (2014). Transpiration efficiency: new insights into an old story. J Exp Bot.

[10] Richter GM, Lawlor DW, Acutis M. Field scale water-use efficiency inherent variability and options for crop selection and management Veneto regional environment energy, special projects office view project advancing earth observation applications in agriculture view project. 2007. https://www.researchgate.net/publication/275020677.

[11] Vadez V, Kholova J, Zaman-Allah M, Belko N (2013). Water: the most important “molecular” component of water stress tolerance research. Funct Plant Biol.

[12] Kar S, Tanaka R, Korbu LB, Kholová J, Iwata H, Durbha SS (2020). Automated discretization of ‘transpiration restriction to increasing VPD’ features from outdoors high-throughput phenotyping data. Plant Methods.

[13] Medina V, y Teran JCBM, Gepts P, Gilbert ME (2017). Low stomatal sensitivity to vapor pressure deficit in irrigated common, lima and tepary beans. Field Crops Res.

[14] Sinclair TR (2018). Effective water use required for improving crop growth rather than transpiration efficiency. Front Plant Sci.

[15] Araus JL, Kefauver SC (2018). Breeding to adapt agriculture to climate change: affordable phenotyping solutions. Curr Opin Plant Biol.

[16] Nicolás-Cuevas JA, Parras-Burgos D, Soler-Méndez M, Ruiz-Canales A, Molina-Martínez JM (2020). Removable weighing lysimeter for use in horticultural crops. Appl Sci.

[17] Jiménez-Carvajal C, Ruiz-Peñalver L, Vera-Repullo JA, Jiménez-Buendía M, Antolino-Merino A, Molina-Martínez JM (2017). Weighing lysimetric system for the determination of the water balance during irrigation in potted plants. Agric Water Manag.

[18] Halperin O, Gebremedhin A, Wallach R, Moshelion M (2017). High-throughput physiological phenotyping and screening system for the characterization of plant–environment interactions. Plant J.

[19] Ryan AC, Dodd IC, Rothwell SA, Jones R, Tardieu F, Draye X (2016). Gravimetric phenotyping of whole plant transpiration responses to atmospheric vapour pressure deficit identifies genotypic variation in water use efficiency. Plant Sci.

[20] Mazzolai B, Mondini A, Corradi P, Laschi C, Mattoli V, Sinibaldi E (2011). A miniaturized mechatronic system inspired by plant roots for soil exploration. IEEE/ASME Trans Mechatron.

[21] Vera-Repullo JA, Ruiz-Peñalver L, Jiménez-Buendía M, Rosillo JJ, Molina-Martínez JM (2015). Software for the automatic control of irrigation using weighing-drainage lysimeters. Agric Water Manag.

[22] Guimarães CM, Stone LF, Zito RK (2017). Suscetibilidade do feijão-comum e da soja à deficiência hídrica avaliada na plataforma de fenotipagem sitis. Biosci J.

[23] Deva CR, Urban MO, Challinor AJ, Falloon P, Svitákova L (2020). Enhanced leaf cooling is a pathway to heat tolerance in common bean. Front Plant Sci.

[24] Jaramillo Roman V, van de Zedde R, Peller J, Visser RGF, van der Linden CG, van Loo EN (2021). High-resolution analysis of growth and transpiration of quinoa under saline conditions. Front Plant Sci.

[25] Adachi S, Tsuru Y, Kondo M, Yamamoto T, Arai-Sanoh Y, Ando T (2010). Characterization of a rice variety with high hydraulic conductance and identification of the chromosome region responsible using chromosome segment substitution lines. Ann Bot.

[26] Maylani ED, Yuniati R, Wardhana W. The effect of leaf surface character on the ability of water hyacinth, *Eichhornia crassipes* (Mart.) Solms. to transpire water. In: IOP conference series: materials science and engineering. IOP Publishing Ltd; 2020.

[27] Load cell—10kg (TAL220)—SparkFun electronics. https://www.sparkfun.com/products/13329. Accessed 3 Mar 2023.

[28] Muller I, Machado De Brito R, Pereira CE, Brusamarello V (2010). Load cells in force sensing analysis-theory and a novel application a ring-type load cell. IEEE Instrum Meas Mag.

[29] Lakshminarayanan V, Sriraam N. The effect of temperature on the reliability of electronic components. In: 2014 IEEE international conference on electronics, computing and communication technologies (CONECCT). 2014.

[30] Temperature sensor—TMP36—SEN-10988—SparkFun electronics. https://www.sparkfun.com/products/10988. Accessed 8 Feb 2022.

[31] Guo S-W, Yi Z, Song N, Qi-Rong S (2006). Some physiological processes related to water use efficiency of higher plants. Agric Sci China.

[32] Behrangi A, Loikith PC, Fetzer EJ, Nguyen HM, Granger SL (2015). Utilizing humidity and temperature data to advance monitoring and prediction of meteorological drought. Climate.

[33] Valipour M (2017). Analysis of potential evapotranspiration using limited weather data. Appl Water Sci.

[34] SONOFF Si7021 temperature humidity sensor for TH10 and TH16. https://itead.cc/product/sonoff-si7021/. Accessed 12 Dec 2021.

[35] Peristaltic liquid pump with silicone tubing—12V DC power—the Pi Hut. https://thepihut.com/products/peristaltic-liquid-pump-with-silicone-tubing-12v-dc-power. Accessed 9 Feb 2022.

[36] Rosadi A, Fauzan A, Winarno. Prototype design of automatic plant watering equipment with soil moisture detection system based on arduino uno microcontroller: case study of chili plant. In: Journal of physics: conference series. Institute of Physics Publishing; 2020.

[37] ATmega328 8-Bit AVR MCUs—microchip technology | Mouser. https://co.mouser.com/new/microchip/atmelatmega328/. Accessed 9 Feb 2022.

[38] Buy a Raspberry Pi Zero W—Raspberry Pi. https://www.raspberrypi.com/products/raspberry-pi-zero-w/. Accessed 9 Feb 2022.

[39] Discover the UDOO NEO | your personal IoT maker board. https://www.udoo.org/udoo-neo/. Accessed 11 July 2021.

[40] Diaz Rodriguez H. Desarrollo de una plataforma basada en IOT para el monitoreo y control de riego en invernaderos. Universidad Autónoma de Occidente. 2021.

