# Supplementary material for: LysipheN: a gravimetric IoT device for near real-time high-frequency crop phenotyping: a case study on common beans
Source: Plant Methods. 2024 Mar 14;20:39. doi: 10.1186/s13007-024-01170-x (PMC10938686; doi:10.1186/s13007-024-01170-x)
Supplement: Supplementary file 1 — Additional file 1. Development process, tests, results and issues from the previous prototype versions of LysipheN. [file 13007_2024_1170_MOESM1_ESM.docx]

# **Additional file 1**

Contents

[**Additional file** 1](#_Toc143165824)

[List of Figures 2](#_Toc143165825)

[List of Tables 2](#_Toc143165826)

[1. Prototype Development Process 3](#_Toc143165827)

[1.1. First Gravimetric Unit prototype version development 3](#_Toc143165828)

[1.2. Second Gravimetric Unit prototype version - improvement and corrections to the first version …………………………………………………………………………………………………………………………………………..10](#_Toc143165829)

[1.3. Third Gravimetric Unit prototype version – issues corrections to 2^nd^ version 24](#_Toc143165830)

[1.4. Final Gravimetric Unit prototype version - Improvement and new features to the previous version… 33](#_Toc143165831)

[2. Phenotyping Platform Improved and SQL database. 33](#_Toc143165832)

[References 34](#_Toc143165833)

## List of Figures

[Figure S1.. 3](#_Toc99352014)

[Figure S2. 4](#_Toc99352015)

[Figure S3. 4](#_Toc99352016)

[Figure S4 5](#_Toc99352017)

[Figure S5. 5](#_Toc99352018)

[Figure S6 6](#_Toc99352019)

[Figure S7. 6](#_Toc99352020)

[Figure S8.. 8](file:///D:\OneDrive%20-%20CGIAR\Documents\DocumentsD\Programas%20Unidades%20Finales%20con%20Tarjetas\Version%20diseño%20final\Manuscript\Reviews\Supplementary%20document.docx#_Toc99352021)

[Figure S9.. 8](#_Toc99352022)

[Figure S10. 9](#_Toc99352023)

[Figure S11. 10](#_Toc99352024)

[Figure S12. 11](file:///D:\OneDrive%20-%20CGIAR\Documents\DocumentsD\Programas%20Unidades%20Finales%20con%20Tarjetas\Version%20diseño%20final\Manuscript\Reviews\Supplementary%20document.docx#_Toc99352025)

[Figure S13 12](#_Toc99352026)

[Figure S14. 13](#_Toc99352027)

[Figure S15. 13](#_Toc99352028)

[Figure S16. 14](#_Toc99352029)

[Figure S17. 15](#_Toc99352030)

[Figure S18 16](#_Toc99352031)

[Figure S19 17](#_Toc99352032)

[Figure S20 17](#_Toc99352033)

[Figure S21. 18](#_Toc99352034)

[Figure S22 19](#_Toc99352035)

[Figure S23. 20](#_Toc99352036)

[Figure S24. 21](#_Toc99352037)

[Figure S25 22](#_Toc99352038)

[Figure S26 23](#_Toc99352039)

[Figure S27 24](#_Toc99352040)

[Figure S28. 25](#_Toc99352041)

[Figure S29. 28](#_Toc99352042)

[Figure S30. 29](#_Toc99352043)

[Figure S31. 30](#_Toc99352044)

[Figure S32 31](#_Toc99352045)

[Figure S33 32](#_Toc99352046)

[Figure S34 33](#_Toc99352047)

[Figure S35 34](#_Toc99352048)

[Figure S36 36](#_Toc99352049)

[Figure S37. 37](#_Toc99352050)

[Figure S38. 38](#_Toc99352051)

[Figure S39 39](#_Toc99352052)

[Figure S40. 40](#_Toc99352053)

## List of Tables

[Table S1.. 25](#_Toc99352362)

[Table S2.. 30](#_Toc99352363)

[Table S3 33](#_Toc99352364)

## Prototype Development Process

The first versions of GU (Figure S4) were equipped with a capillary tube (point 1), which drives water inside the pot (bottom part). This became a not efficient system that obstructed the normal weighing system’s working because the watering pipe went through the whole system from the bottom. Additionally, the pot’s base was very susceptible to vibrations, which add some noise to the weight system. Besides, the prototype saved data through Wi-Fi only, and the Esp32 board showed low effectiveness under greenhouse conditions, which affected weight measurements and caused data losses (Figures S10 and S11).

Thus, the mechanical structure of the prototype was first designed on SolidWorks (Figure S1) software to know what it will look like and the materials needed for the building process.


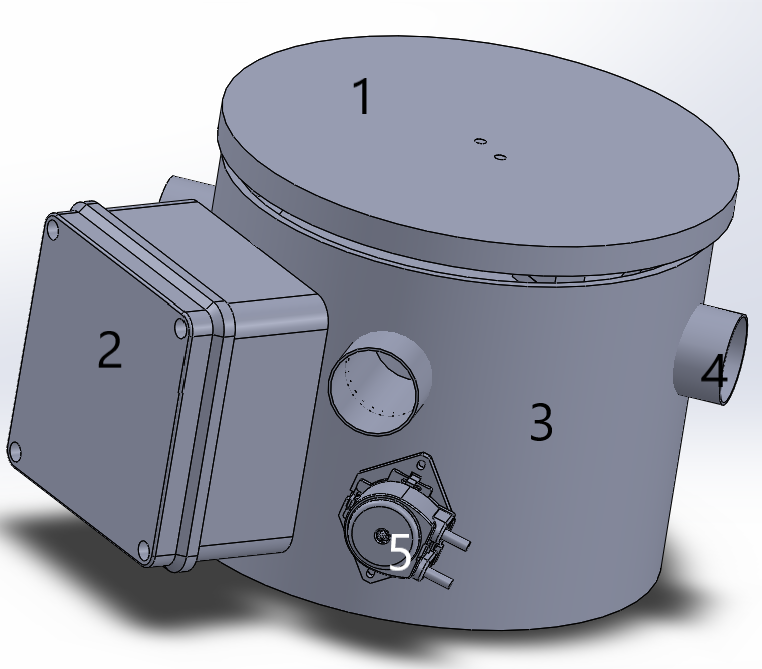


Figure S1. Mechanical structure in CAD design, SolidWorks. 1 Base for placing pot. 2. Electrical Ip65 Box. 3. Circular PVC base. 4. Output holes for connecting sensors and dripping irrigation tubes. 5. Peristaltic pump for irrigation.

However, adaptations were needed to satisfy new ideas and project necessities. Thus, developing gravimetric units’ prototypes, attempted different test stages.

### First Gravimetric Unit prototype version development

First efforts to develop a new low-cost IoT lysimeter called the “Gravimetric Unit “prototype, were focused to test the different components separately and subsequently together with the first mechanical structure mounting. The prototype’s mechanical structure was built in a handmade way using low-cost rustic elements, which are possible to easily acquire at any commercial store. These elements majority polyethylene, are enough capable to support the system’s weight (around 8 kg). (Figure S2 to Figure S4).

In addition, the first version was integrated with a soil moisture sensor, Air temperature, and humidity sensor, and load cell (weight sensor). These were connected to an ESP32 development board (Esspresif), which can be able to read digital and analog signals at 12 bits resolution. This board was programmed to store locally and send data through Wi-Fi, to the server where the phenotyping platform’s application was alloyed.

Automatic on-off irrigation was also added using a single 5V relay and a 12V peristaltic pump. For irrigation, the first version prototype was equipped with a tube for hose conduction, which was used for irrigating by capillarity, but it was removed in the next version (see below).


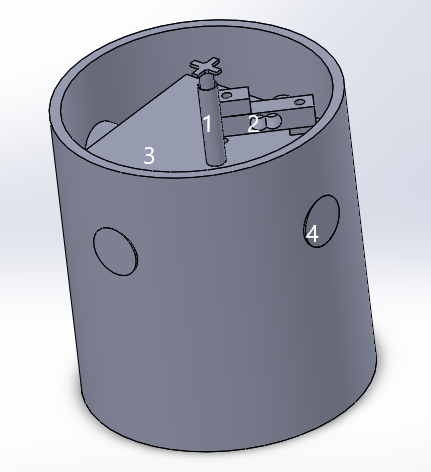


Figure S2. Mechanical structure CAD Internal view, Solidworks. 1 Capillarity irrigation system without friction with the weighing system (not used in the next version), 2. Weight sensor, 3. Square base box, 4. Output holes for tubes for protecting and driving cables sensors and dripping irrigation hose.


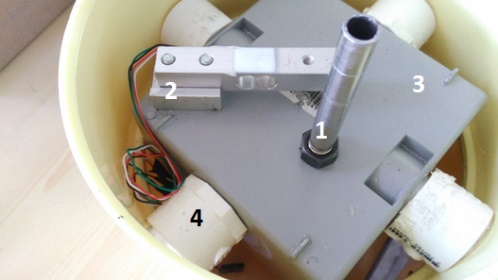


Figure S3. Mechanical structure built Internal view. 1 Capillarity irrigation system without friction with the weighing system (not used on next version, 2. Weight sensor, 3. Square base box, 4. Output holes for tubes for protecting and driving cables sensors and dripping irrigation hose.


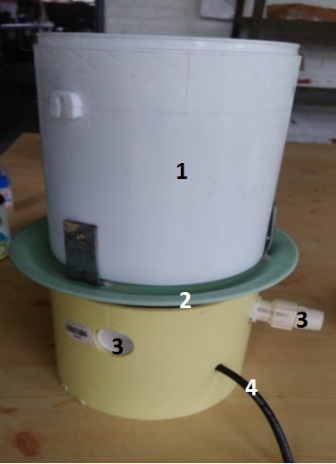


Figure S4. Mechanical structure built external view. 1 Pot for soil placing and plant sowing, 2. Pot’ base using a circular plate for stability connection with load sensor below, 3. Output holes for tubes for protecting and driving cables sensors and dripping irrigation hose.

For load cell measurements as an instrumentation amplifier, was used the HX711 (A/D converter) was in this first version. Which is a 24 bits analog-to-digital converter with a programmable gain. Data from the converter were read using [1].


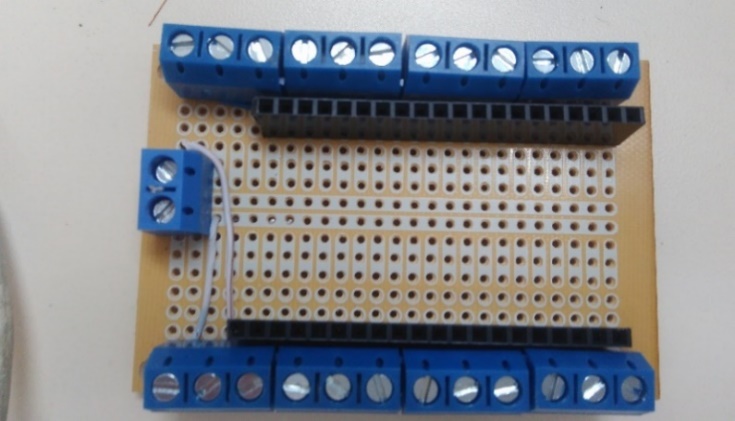


Figure S5. First electronical embed circuit prototype with bakelite built for first experiments (Top view).

Thus, previous electronic devices were joined in the electronical board made on bakelite (Figure S5 and Figure S6) for these first experiments where different sensors and actuators were connected with Esp32 and micro-SD card.


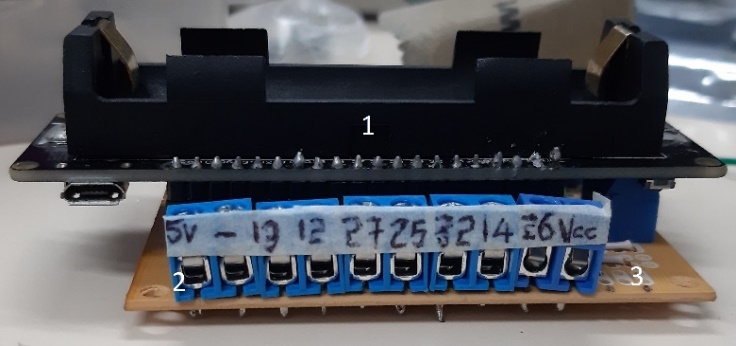


Figure S6. First electronical embed circuit prototype with bakelite built for first experiments (horizontal left view)., 1. Esp32 assembled on board, 2. I/O board pins, 3. Bakelite board.

Variables and devices before mentioned were connected also with the environment sensor and control irrigation for conforming joints to the mechanical structure of the prototype version and beginning experiments with plants (Figure S7).


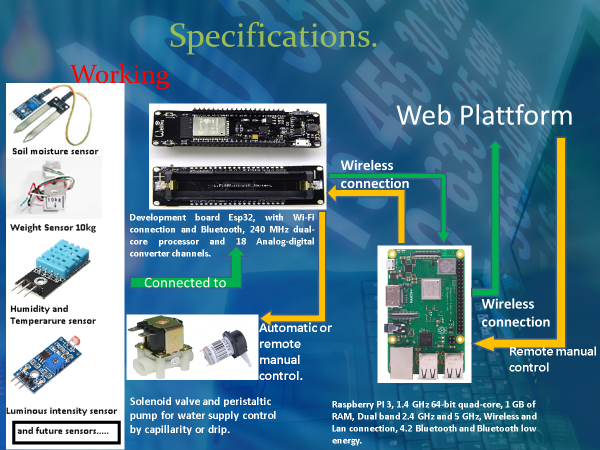


Figure S7. Communication protocol left to right, sensors, and actuators connected to ESP32 which is connected using Wi-Fi with Raspberry Pi which is the server where the phenotyping platform is running.

Thus, we had a system able to support and measure a pot-soil-plant system. Since, with plants, different assemblages, connections, communications, calibrations, and corrections tests were made. In almost all cases the main issue to solve was load cell (weight sensor) measurement stability and noise corrections. This is expected, as [2] wrote that a noise source that could be considered in a weighing system can come from soil constitution behavior, due to irrigated water replacing air contained in the macro and micro pores; then, there is a movement of the water through the soil, this movement can occur both, vertically and horizontally. But even with these considerations, weighing systems can be able to give kind of stable measurements.

It is important to mention that an expected behavior in the normal weighing system during every crop life cycle day; could be based on results from [3], where weight increases during irrigation time until the selected target, after reaching this reference; Weight decreasing process should be higher during daytime than nighttime due to evapotranspiration. Below, can give ideas about how the normal working behavior should be in a plant weighing system.

#### Water irrigation control

[4], proposed different options to evaluate water consumption to irrigate plants in each pot individually. The consumed water can be replaced by plant irrigation while maintaining a nearly constant soil water status throughout the whole day. In addition, by gravimetrical correction for the plant biomass increase, a water expenditure curve can be obtained. Besides, the irrigation process of the plant is crucial to perform almost automatic plant growth and monitoring and also minimizing the service time [5]. We present in our study that the gravimetric unit has a sophisticated automatic water irrigation system. Irrigation was rationed with high-precision actuators which were also used to support two different irrigation systems (drips from above, classic bottom irrigation). Having both systems will allow for the growth wider range of plants, including lowland rice under simulated flooding. Understanding that the accuracy of using weighing lysimeters to maintain the water balance in the soil will depend, among others, on the knowledge of the initial water content in the soil [5].

The gravimetric unit has an algorithm for consulting local network time every 10 seconds in cases (experiments) where irrigation control by a specific time is required. The data of actual transpiration or tissue (leaf, bud, flower, pod) temperature are immediately available after any water supply and will serve as an important source of genotype-based variability in water-related behavior (water spenders, water savers), heat, and other stress resistance mechanisms.

For the precise control of the irrigation system, a 12V peristaltic pump [6] was used (Figure S8) and connected to a digital relay which is an electronic device used to implement switching on/off logic (A Rosadi et al., 2020). The relay can turn on/off the pump according to the irrigation scheme. This pump is a precise low-power consumption device. Besides, the peristaltic pump can be calibrated, to estimate a minimum water volume always present in tubes (not affecting soil water status) after each run of irrigation and depending on opening and closing times (in seconds). During each experiment, irrigation process parameters may easily be changed depending on user or plant requirements accordingly to the type of water availability scenario. As a first configuration, we choose one irrigation each day at a specific programmed time (usually early mornings).


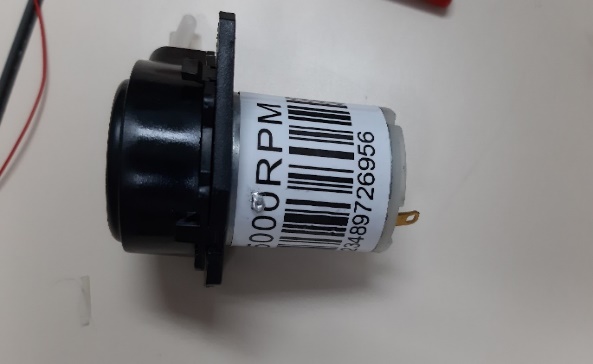


Figure S8. Peristaltic 12v pump.

#### First Phenotyping Platform version

In its first version [7], for the IoT-based greenhouse automation platform a web application was developed (Fig Figure S9) based on NodeJS, which is a JavaScript programming environment for the Backend based on the V8 JavaScript engine of the Google Chrome browser and is event-oriented, non-blocking, which makes it very fast when creating servers, web and use real-time. In this version, the main objective was wired communication between devices and the storage of the information in a CSV file.


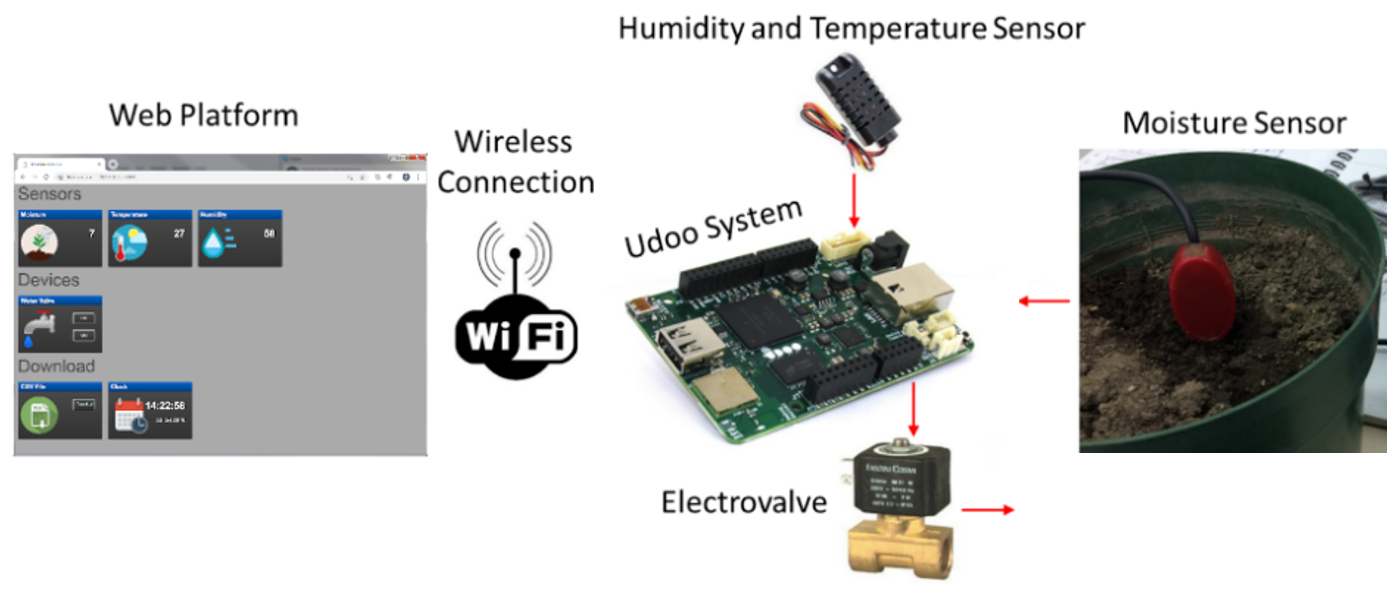


Figure S9. First Gravimetric Platform version.

#### First gravimetric unit prototype version - test under greenhouse conditions

It is important to mention that an expected behavior in the normal weighing system during every crop life cycle day; could be based on results from [3], where weight increases during irrigation time until the selected target, after reaching this reference; Weight decreasing process should be higher during daytime than nighttime due to evapotranspiration. Below, can give ideas about how the normal working behavior should be in a plant weighing system.

Thus, the first experiment consisted of using the first gravimetric unit prototype version with one replica built together with two SEF60 bean seeds sown (one per unit) inside the pot placed over each top prototype. Each unit provided water irrigation automatically each day based on a target value to reach depending on weight loss and the time to irrigation was defined by an internal programmed timer (every 24 hours). The test was carried on over almost 40 days to identify structural, electronical, and plant behaviors.

Structural mounting does not have any problem, but the base weight could be enough low to keep possible movements caused by externals like air, which is not convenient for weighing measurements. Big issues with this version came from electronical and communication board devices.


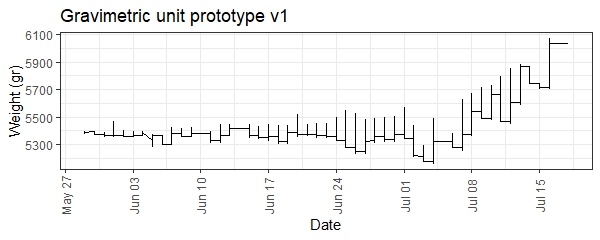


Figure S10. data from weight sensors during 45 days of experiment with two plants, stored every five minutes. Downloaded directly from the phenotyping platform. First prototype version.


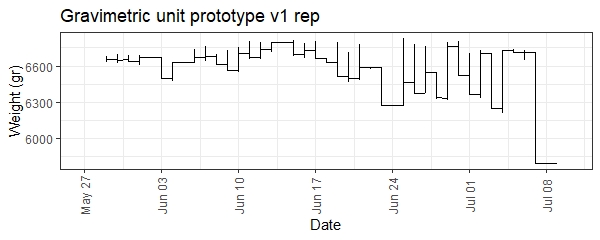


Figure S11. data from weight sensors during 45 days of experiment with two plants, stored every five minutes. Downloaded directly from the phenotyping platform. Replica of the prototype version.

It is possible to note that there is not a logical behavior as expected based on this data. For the two prototypes, Wi-Fi connection issues caused disconnection and conflict with Esp32 libraries. Therefore, data was not even stored locally on each micro-SD card.

As it was shown in (Figure S10 and Figure S11). During the test period, there were data losses (horizontal straight lines) due to the weak Wi-Fi connection of the ESP32 in greenhouse conditions. Each prototype gets disconnected from the Wi-Fi network for hours, these time intervals were filled automatically with the last received value from the phenotyping platform which required data every five minutes that is why there are horizontal complete straight lines during periods in which post-irrigation water loss and gain were expected. Besides, internal library conflicts did not allow local data storage. Despite this, another big issue was related to data from load cell sensors most specifically with analog to digital amplifiers used (HX711). This device was very susceptible to any external noise which triggered illogical behaviors in the measurements (as discussed above). This, in turn, caused failures in the automatic irrigation that resulted in the established reference points were not respected according to the programmed control logic, and in some cases, the amount of water exceeded or was below the specified amount (5400 and 6700 grams respectively).

In a plant’s growing case. Both plants appeared to develop normally as well as their normal function when planted in a pot. This shows to the design does not affect the normal cycle of the plants (Figure S12).


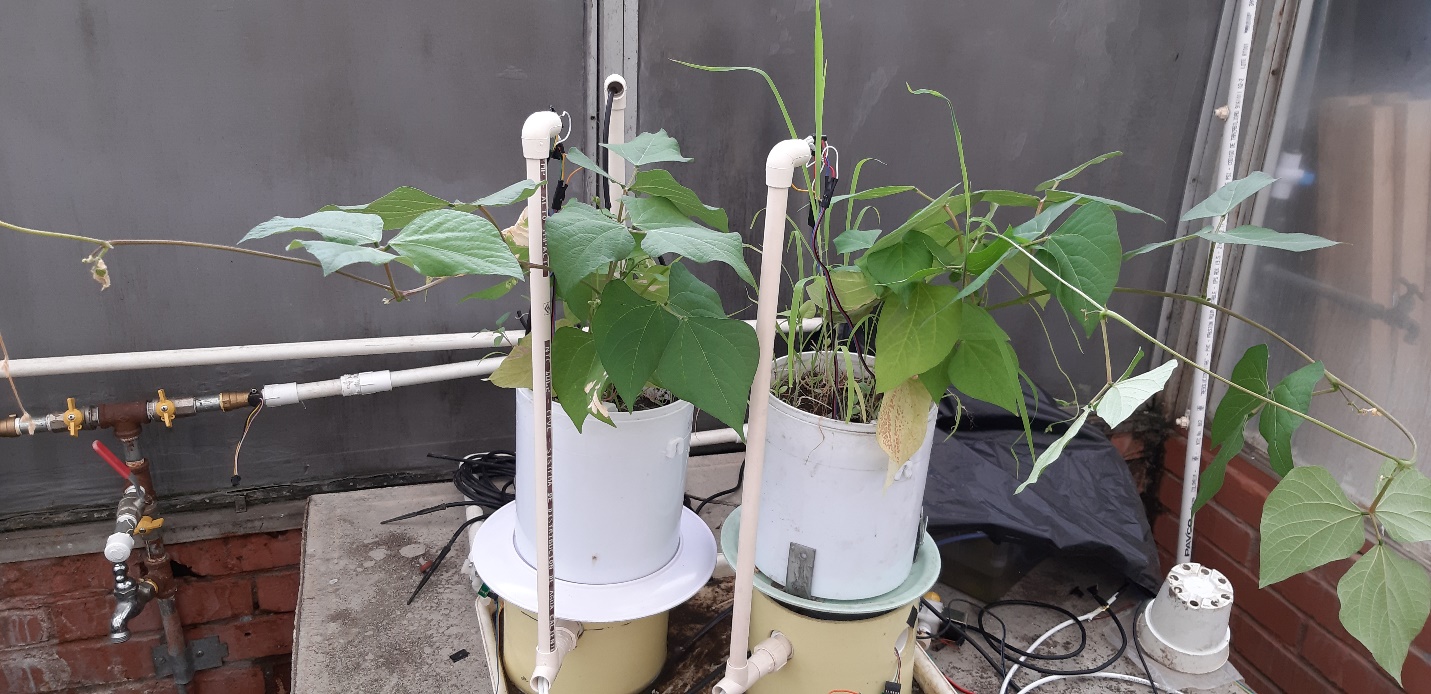


Figure S12. Two plants growing up after 45 days experiment. Gravimetric unit first prototype (right) and replica (left) inside greenhouse.

#### First gravimetric unit prototype version - Stability verification

Before continuing with the next development stage, some separate extra verification test was made with the first version built. This short experiment was focused to see weight measurement behavior inside greenhouse conditions with a fixed 2000 gr weight (iron piece) for 4 days. Placing an abiotic iron piece over the gravimetric unit. Expected behavior during the time was a kind of constant or straight value measured.


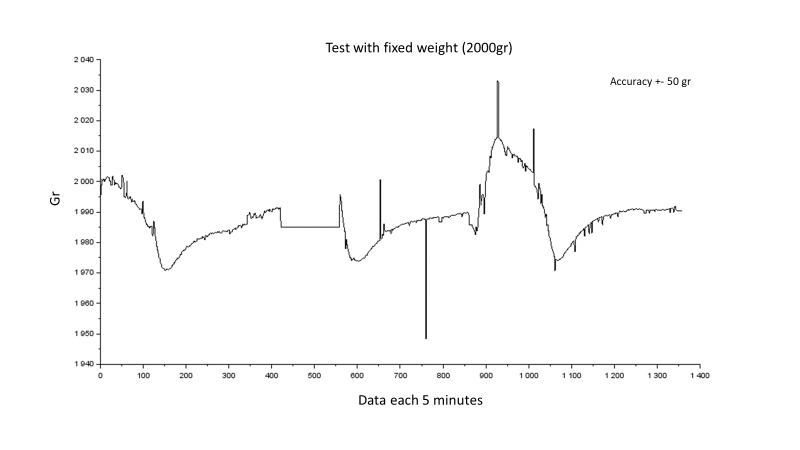


Figure S13. Weighing system test with a fixed weight in greenhouse conditions.

Whit a fixed non-changing weight showed that the system was very susceptible to external noise as air movement or environmental changes (Figure S13). Due to this, a new method to amplify and read load cell output. In this case, a very commonly used strain gauge based on the Wheatstone bridge is the AD620 amplifier, which gives an analog output based on a previously defined gain using resistors. Besides, this is an instrumentation operational amplifier with low power consumption and is widely used in conditioning amplifying systems for different purposes [8,9].

### Second Gravimetric Unit prototype version - improvement and corrections to the first version

From the first version, weight measurement showed to be a critical part of prototype development. This variable represents the possibility of quantifying per-plant transpiration, soil water content, and irrigation control during the entire growing season. However, external influences could cause measurement issues and derived control mistakes. And even, some of these issues can be caused by wind or vibrations (Jiménez-Carvajal et al., 2017). Therefore, weight stability was considered the main challenge of this project. Thus, the weighing system of the prototype version was also tested with stable weight (Figure S13). Data showed that the accuracy error reached 50g, which means unacceptable changes in a system that must be able to register smaller changes (+-10g). Therefore, as the weighing system was tested and adjusted, the electronical design was modified to obtain an equilibrium between data quality and stability.

After multiple calibrations, stability verifications, and re-designs were done to find the cleanest signal from the weight sensor. The second version of the Gravimetric Unit prototype was done (Figure S18).

A new improvement feature was added to the design. Using a Teflon wire thermocouple extension with a tiny end welded internal pod and stem temperature measurements were added to gravimetric units. Connected with the selected amplifier Max6675 and connected using [10] to ESP32 board to read data from each sensor. In the pod’s case, inside and closer to the Apice or Pedicel has inserted the junction. For the internal stem temperature measurements, reached specified time junction can be placed in order to not affect the normal plant life cycle.

#### Internal pod and stem temperature measurements

The temperature is a variable climatic factor with a recognized influence on plant physiological responses. A method with temperature sensor (thermocouple) insertion inside plant stems was developed (Cárdenas et al., 2019), to measure the sap flow. Our system can measure internal pod and stem temperature by using the K-type thermocouple (K-type Teflon thermocouple with Nickel chromium and Nickel aluminum alloy) (Uxcell K-Type Thermocouple Wire 2 Core AWG 24 High-Temperature PTFE Insulation 6.6ft- Buy Online in Bermuda at Desertcart - 175775803., n.d.). (Figure S14). The thermocouple has an adapted welded end (Figure S15). Temperature changes applied to this union generate a small voltage as an output (mV) (Seebeck effect).

Thus, each adapted thermocouple needs a signal amplifier system due to the original output it is in the mV. To solve it, thermocouples were connected to the 12 bits analog-digital converter MAXX6675 [12]. widely used to convert signals from similar sensors and used in different monitoring and controlling processes as it was done by [13]. This module was connected to microcontroller input pins using SPI communication and with the appropriate library, the raw data was converted to temperature in ℃.

Thus, we used these adapted sensors to measure internal stem and pod temperatures (two sensors per GU). These data can serve as a base of useful information for plant modelers as different tissue temperatures should serve as a more precise estimation of the temperature effect on growth and development in a changing climate. In this phase, internal stem and pod temperature were measured to discover possible correlations between the other variables and plant physiological behavior and verify the effect of direct irradiation.


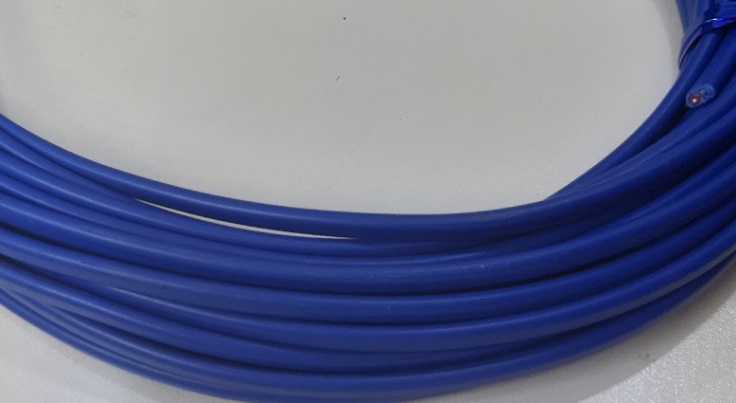


Figure S14. K-type Teflon with Nickel chromium and Nickel aluminum alloy (Uxcell K-Type Thermocouple Wire 2 Core AWG 24 High-Temperature PTFE Insulation 6.6ft- Buy Online in Bermuda at Desertcart - 175775803., n.d.) used for gravimetric units.


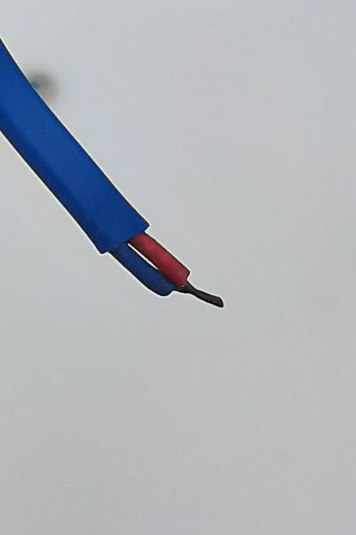


Figure S15. K-type adapted thermocouple Teflon with Nickel chromium and Nickel aluminum alloy with tiny ends welded, and temperature changes applied on this junction are measured.

#### Electronical board’s temperature measurement

There is a temperature-based influence on the circuits from the load cell (weight sensor). [3] in their development used four load cells connected as a full Wheatstone bridge with a temperature-compensated system. Besides, the analog to digital converter datasheet (ADS1232, used for gauge signal amplification) showed a ratio between offset noise and temperature; despite, changes in nV term, temperature noise is a variable to correct in order to obtain more precise data. Thus, in our system, the temperature corrections are done as the electronical board temperature is also measured. For this purpose, the temperature sensor TMP36 [14] was used to measure the air temperature to which the electronical board was exposed. The data from this sensor were used to run an auto-compensation temperature calibration algorithm for more precise weight measurements.


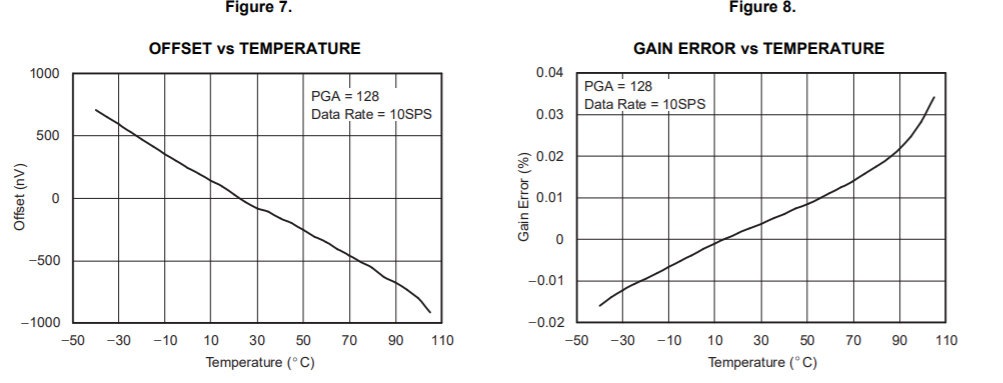


Figure S16. noise comparison with temperature, from ADS1232 Texas instruments datasheet. Both OFFSET and GAIN variables play a crucial role in signal amplification. OFFSET value is used for calibrating the process. Gain factor, reefers to times to be amplified data from load cell.

Besides, within new features added to the prototype was an Atmega328p microcontroller connection to data acquisition from all sensors and stored locally in a micro-SD card. Afterward, it is responsible for sending data using serial communication to the Esp32 board to can be sent to the phenotyping platform remotely.

Additionally, the handmade electronic embedded system (Figure S6) was changed by one designed using (EAGLE | PCB Design and Electrical Schematic Software | Autodesk, n.d.)was designed (Figure S17) and with [16] the first version of embedded electronical board was built to replace the bakelite previous board.


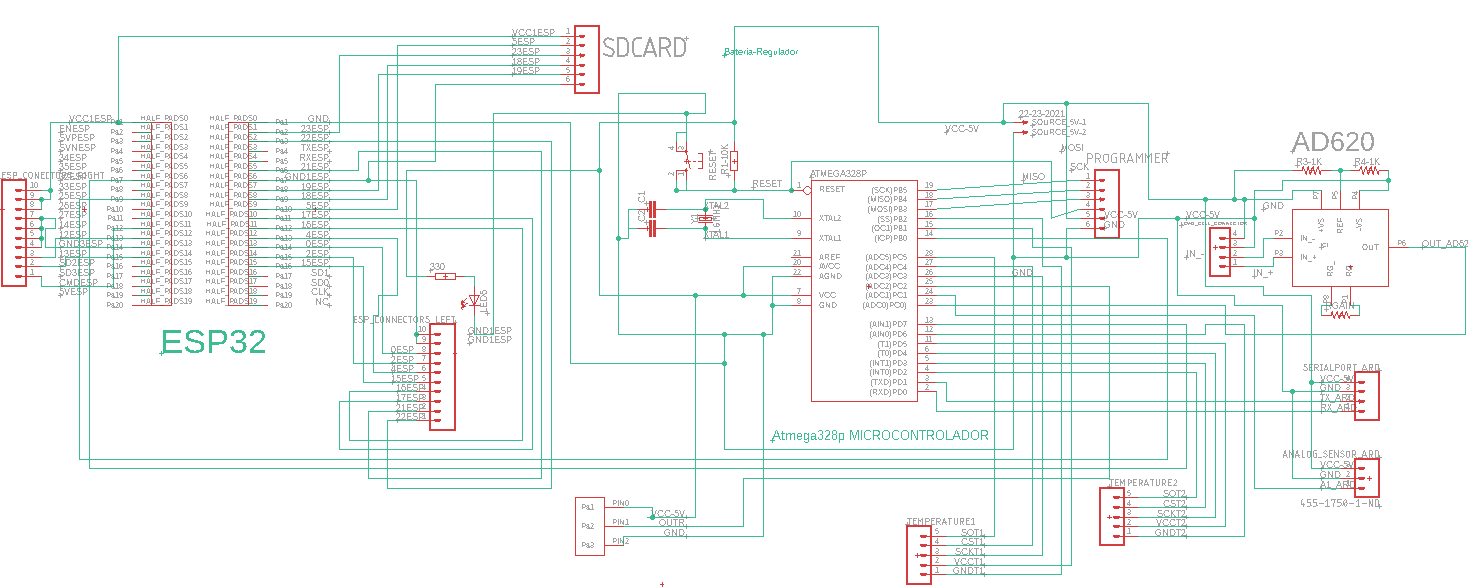


Figure S17. Design of the first version of embedded electronical board

The embedded electronical board was equipped with:

- The weighing system (load cell, electric filter, and AD620 amplifier) is wired to the Atmega328p microprocessor.
- Esp32 board.
- Two K-type (tiny wire end welded) thermocouples (temperature sensors),
- Relay to ON/OFF peristaltic pump control.
- Specific analog I/O,
- Serial port.
- A programmer port for Atmega328p programming (only external).
- A micro-SD card.
- 11 Gpio pins.


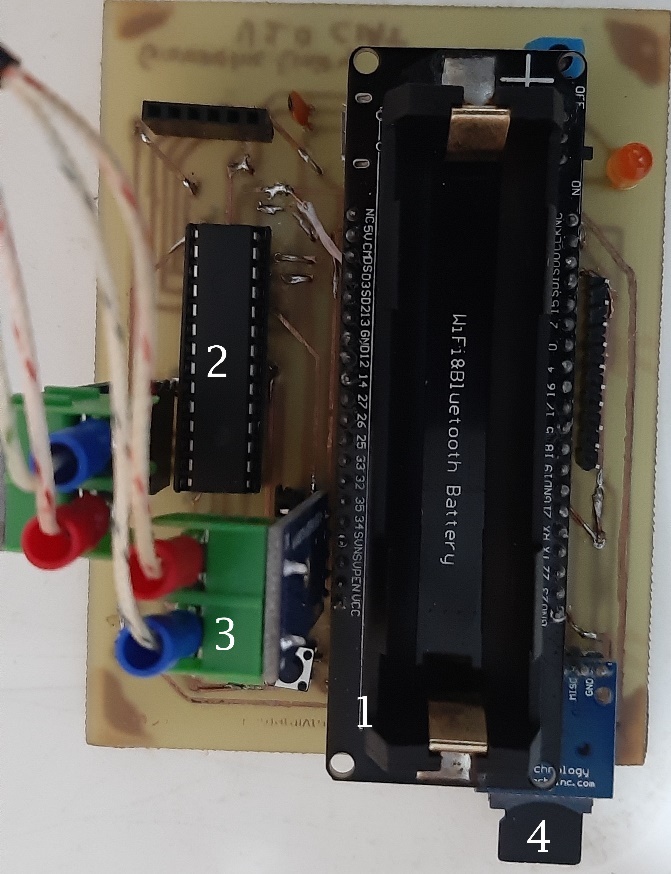


Figure S18.Design of the first version of the embedded electronical board. 1, Esp32 Wi-Fi board. 2. Atmega 328p microcontroller. 3, K-type thermocouple signal converter.

Esp32’s board and Atmega328p are internally wired with two serial-specific pins, that are used to communicate between them. The reprogramming process for both devices (Esp32 & Atmega328p) must be done separately. However, both are programmed using C++ language with Arduino IDE.

For correct board working, a continuous communication process was accomplished between twice internal microprocessors in all experiments using a serial protocol and JSON data format. The internal clock of the Atmega328p processor was programmed to every 5 minutes (user’s determination) read sensors data, create a JSON package with all data and send data to Esp32 and wait for a received confirmation; once the answer from Esp32 is received, Atmega328p will execute the irrigation function it is the correct time, otherwise will continue with the normal reading and sending process (Figure S19).

Figure S19. Atmega 328p Routine Programming - send data to ESP32.

Figure S20. ESP32 Routine programming - receiving, storing, and sending data.

Immediately Esp32 read the message sent from Atmega328p, unpacks the JSON message and saves all data in the micro SD card with the current local network time, creates a new JSON package, and is sent using MQTT protocol to the Mosquitto broker (Udoo Neo) (Figure S20), where data are saved in a MySQL database and interfaced in the phenotyping application in real-time.

#### Second gravimetric unit prototype version - Test with plants

With the new changes, the second version of the prototypes was evaluated in a growing bean cycle (Figure S21). Where tiny temperature sensors showed to be able to measure temperature behavior inside the stem and pod without affecting its normal development (Figure S26). The temperature inside the stem was lower in magnitude than the soil temperature, but similar compared with the temperature inside the pod (Table S1). However, significant differences can be expected using contrasting genotypes or exploring abiotic stress responses as limiting water access. On the other hand, data showed un-stability in weight measurements (Figure S27). The second version did not correct the noise.

Thus, the second experiment with plants began using 8 gravimetric unit prototype replicas. Each prototype contained a sown HTA4 bean seed (Figure S21). This experiment was carried through to evaluate the modified version of prototypes working under greenhouse conditions with bean plants with automatic irrigation every day and data sampling every five minutes. Additionally, this version had an important change in the time irrigation schedule. For this experiment, Esp32 consulted time from the internet network, and at determined time irrigation had to be done automatically until reached the target weight point. Besides, the complete test was divided into three stages for data visualization and temperature sensor use and had a duration of 70 days, which was considered enough time to confirm normal plant behavior during the majority of its life cycle.


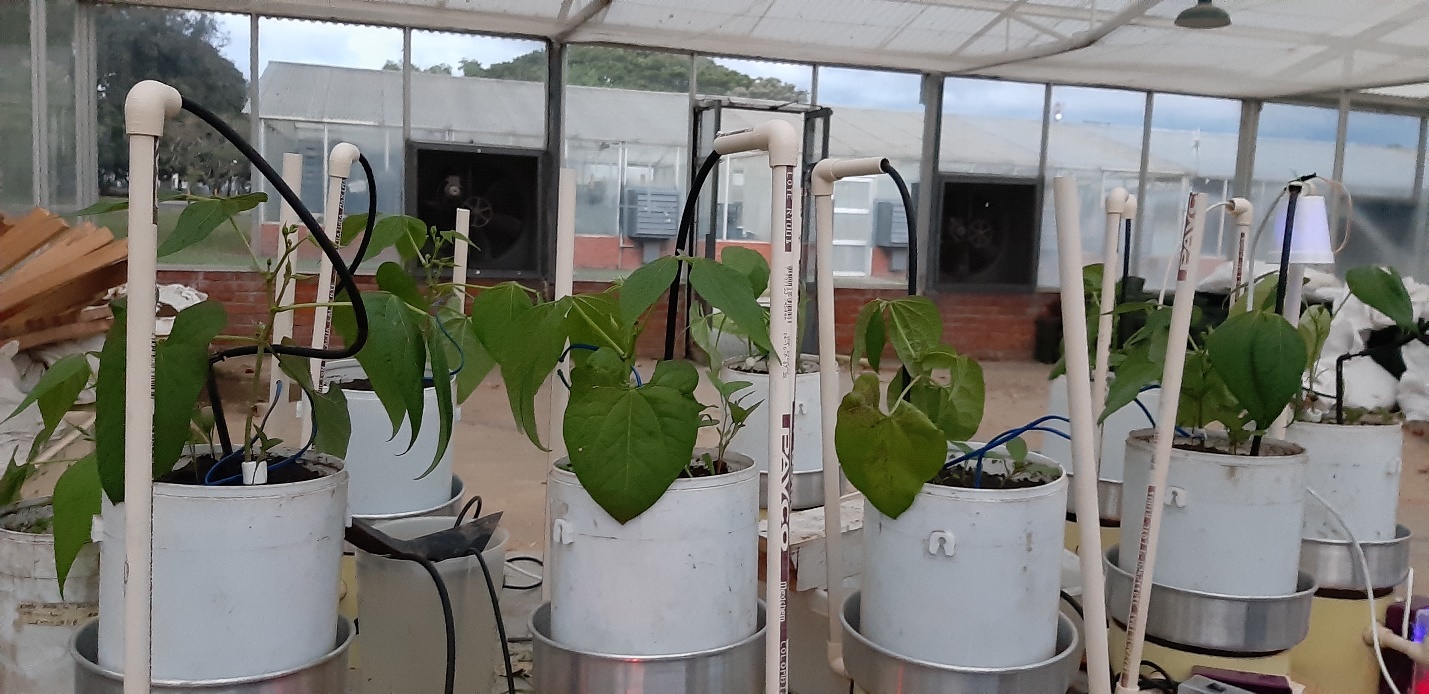


Figure S21. Second prototype – Evaluation with Bean Plants.

##### First Experiment’ Stage

The first stage comprised from day 1 to 18 when plants were in enough growing phase to insert an internal stem temperature sensor. It was focused on monitoring weight changes during the first growing-up plants’ days. However, during this period, internal stem and pod sensors were placed closer to the plant and between them; to measure environment temperature and see behaviors and differences between them.


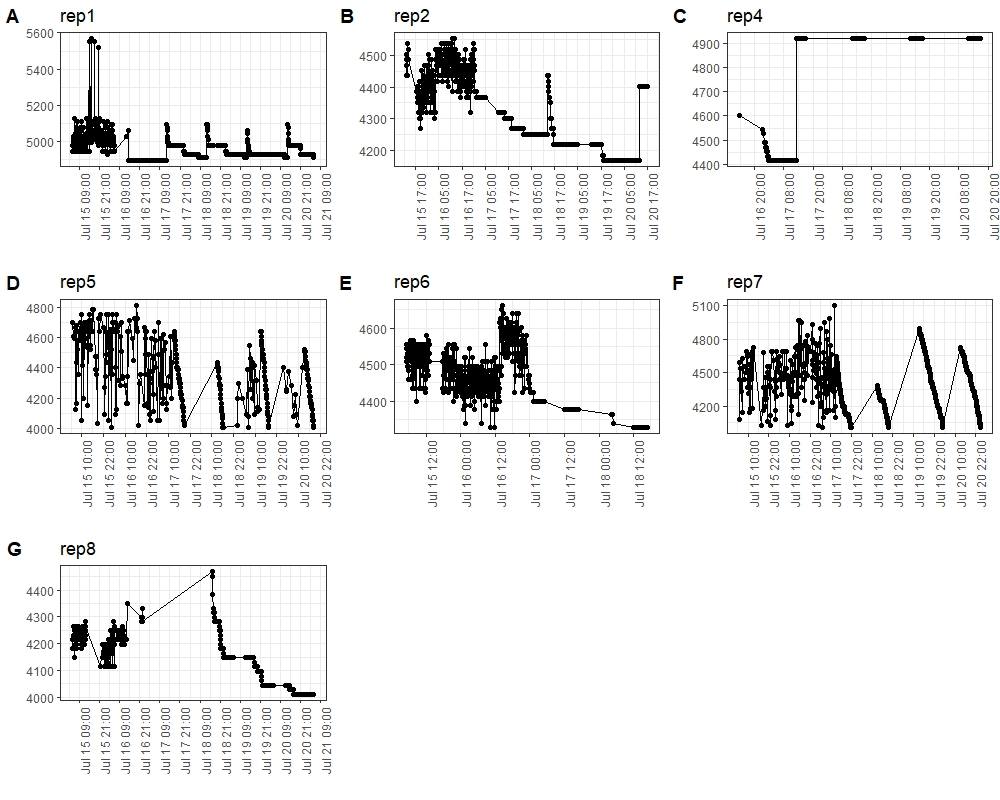


Figure S22. Weight measurements from the first growth stage for eight (A to G) prototype replicas (rep# means the number of prototypes’ replicas). Straight horizontal and diagonal lines correspond to lost data due to remote internet connection issues. Data from C shows almost totally data lost from that prototype. Also, note higher and lower data peaks meaning external noise affecting load cell measurements.

Figure S22 shows data from 7 gravimetric units’ replicas downloaded from the phenotyping platform during the last five days of the first stage. It is possible to note to still there were still lost data periods (straight horizontal and diagonal lines). These issues could come from an incorrect Wi-Fi connection working between Esp32 and the Internet network in the greenhouse. Besides, it is possible to problems with the Wi-Fi connection from ESP32 boards not only caused lost data but also irrigation schedule failures. Additionally, data from rep3 are not included in the figure, this prototype had Wi-Fi failures in this part of the experiment.

Behavior on weight measurements showed big differences between replicas on daily weight losses. Some cases (A, B, E, and G) showed kind of similar behavior regarded to daily weight loss by evapotranspiration of close to 200 and 300 gr of water per day despite clear noise presence. However, these losses in normal conditions mean 200 or 300 ml lost per day. In other replica cases, noise interference is higher which causes more non-logical data.

Two temperature end welded sensors (T1 and T2 respectively) were measuring air temperature behavior during this period (Figure S23). However, note to the differences (in replica 2) between these is very low in magnitude (Table S1).


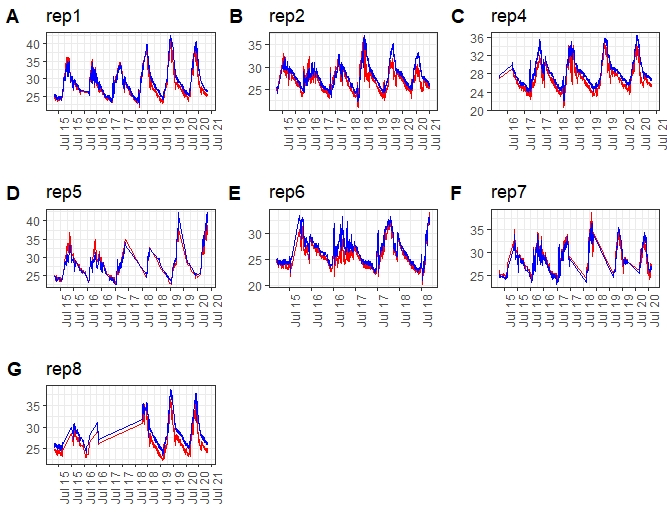


Figure S23. Air temperature measurements from the first growth stage for eight (A to G) prototype replicas (rep# means the number of prototypes’ replicas). Two temperature-adapted sensors T1 (red) and T2 (blue). Straight horizontal and diagonal lines correspond to lost data due to remote internet connection issues. Note to the difference between sensors is very small, even visually some data are transposed over long periods.

##### Second Experiment’ Stage

The second experiment stage was carried through between 19 days to 49 of the bean genotype life cycle. Measurements were done like the first experiment part; however, from day 19, inserted inside the stem temperature sensor, and another one was placed inside the soil surface at 5 cm depth, to compare all data and possible correlations between them. Thus, weight (Figure S24) and temperature (Figure S25) (5 cm depth inside soil and internal stem) changes measurements were taken for data analysis from this period


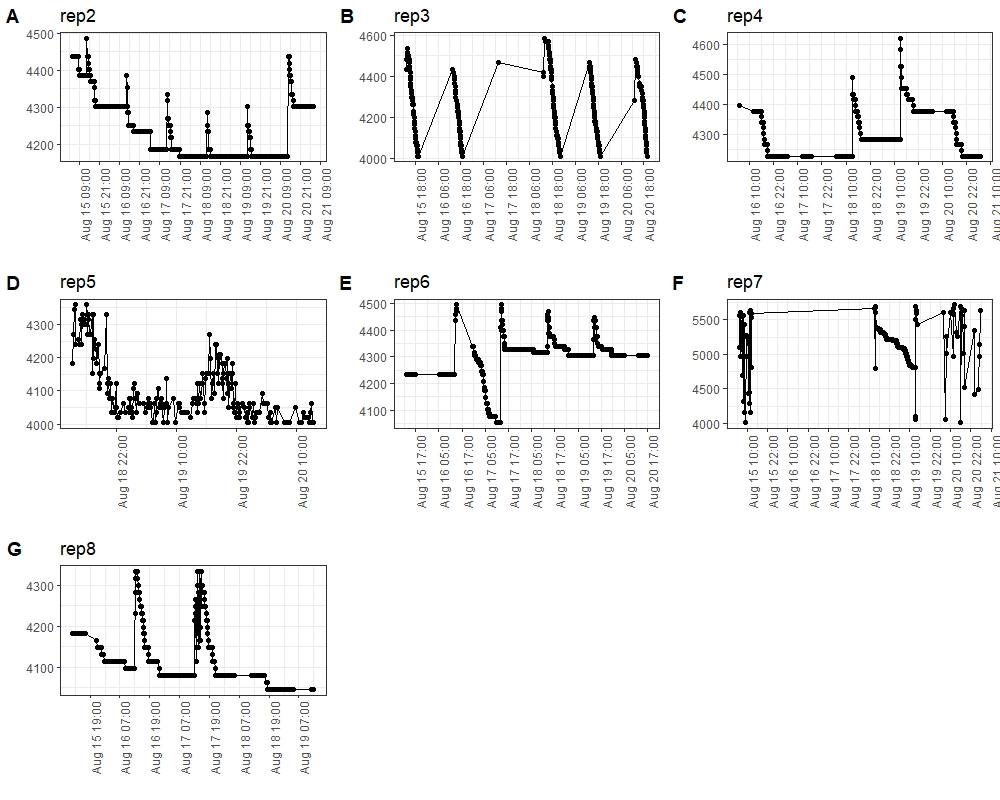


Figure S24. Weight measurements from the second-growth stage for seven (A to G) prototype replicas. Straight horizontal and diagonal lines correspond to lost data due to remote internet connection issues. It is important to mention that due to permanent remote connection failures, one prototype was removed from the experiment. Also, note higher and lower data peaks meaning external noise affecting load cell measurements.


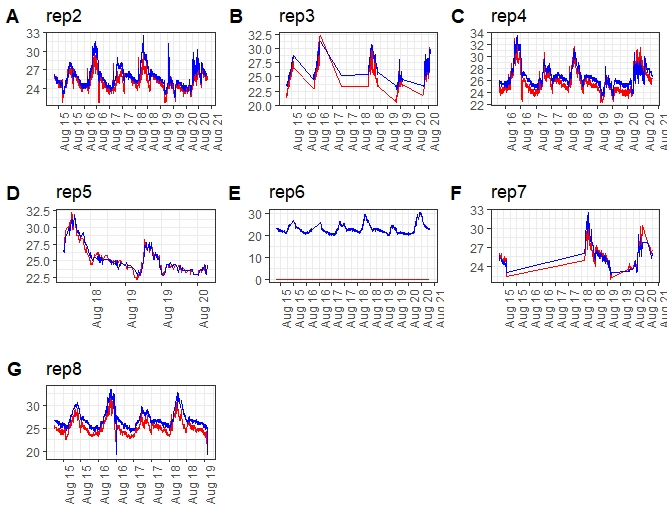


Figure S25. Temperature measurements from the second-growth stage for seven (A to G) prototype replicas. Internal Stem T1 (red) and inside soil T2 (blue; at 5cm from the soil surface). Straight horizontal and diagonal lines correspond to lost data due to remote internet connection issues. Note to the difference between sensors is higher than in the previous stage; prototypes A, B, C, F, and G's internal stem temperatures were lower than soil temperature. Additionally, note to prototype E stem sensor gets disconnected during the entire rest of the experiment.

An important aspect to stand out from these data; is the fact to the number one replica gets permanently disconnected automatically from the network’s internet due to internal issues. The experiment now will continue with seven prototypes only. Additionally, note to straight horizontal and diagonal lines still appear as proof of continuing remote connection issues in all replicas of the prototype.

Weight behavior also shows continuous noise interference in all replicas although to a lesser level in some of these. There is an increment in daily weight loss magnitude (cases A, B, and F) over 300 and 400 ml of water. This could be given by as plants grow, the rate of water absorption, and use increases [17]. This represents an advance for fixing behavior measurement, as expected.

On the other hand, in the temperature case. In the replica 2 case, stem measurements were almost all periods lower than soil temperature in magnitude. But even though these two temperatures were lower than air temperature (from the DHT22 sensor) Table S1 shows minimal and maximal differences between internal stem and soil temperature.

Where it is possible to note that as previously mentioned, differences are bigger due to internal stem temperature being lower than soil temperature. This is since transpiration forces the plant to transport water from the soil to the leaves in the form of sap flow [18] so that at times of higher evaporative demand these flows could be higher and therefore affect the internal temperature.

##### Third Experiment’ Stage

The third’ experiment part between days 50 to 65, was used to see weight behavior during plants’ reaching close to the physiological maturity stage [19]. Specifically, on day 50, some pods had enough size to insert closer to the apice the tiny top welded temperature sensor (before being placed inside the soil’s surface) without affecting (expected) the normal pod’s life cycle (Figure S26).


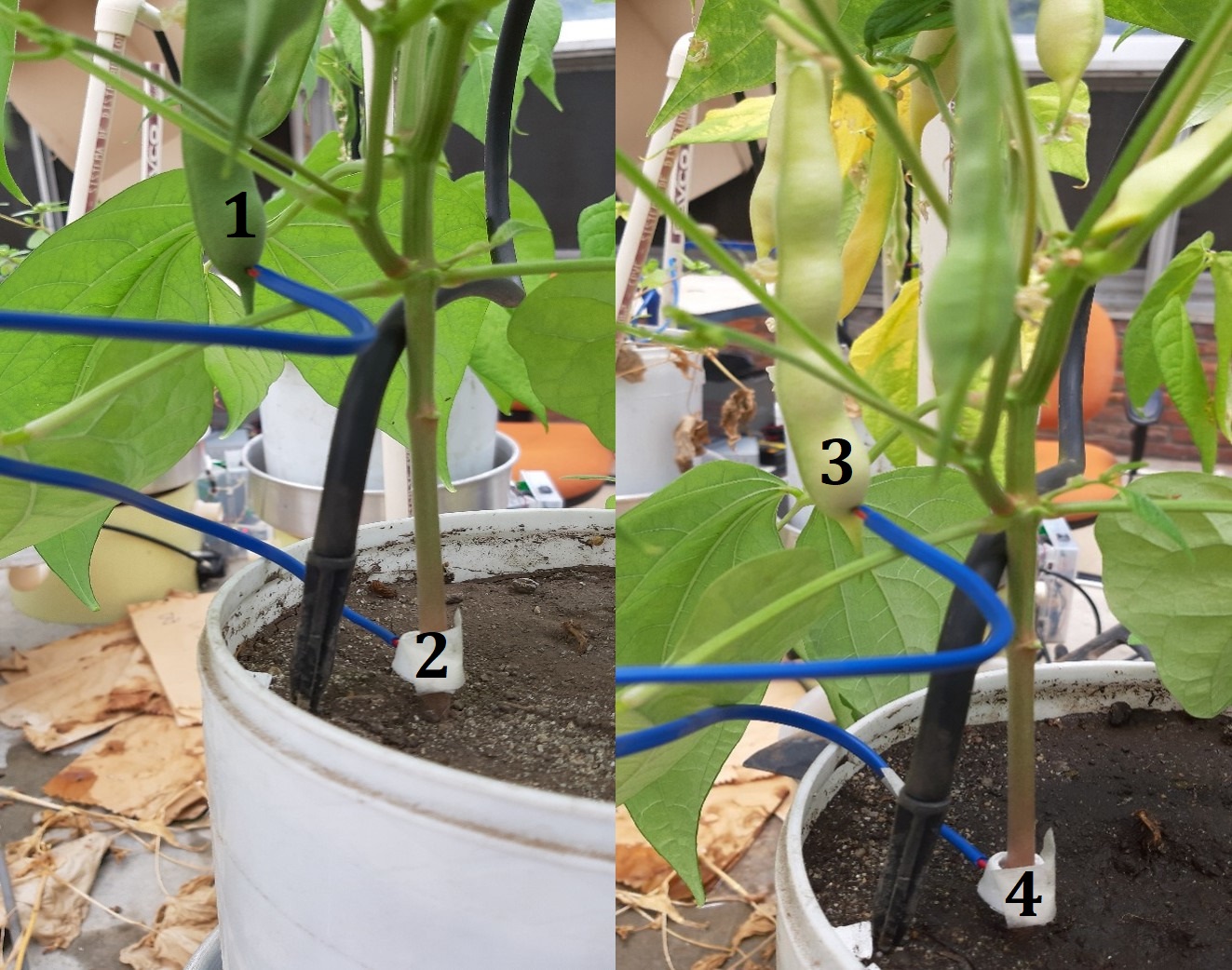


Figure S26. 1, Tiny temperature sensor was inserted into the pod at day 50 after sowing. 2, a Tiny temperature sensor was inserted into the stem on day 19 after sowing. 3, a Tiny temperature sensor is inserted into the pod at the end of the experiment. 4, a Tiny temperature sensor is inserted into the stem at the end of the experiment.

Thus, weight (Figure S27) and temperature (Figure S28) (internal pod and stem) change measurements were taken for data analysis from this period.


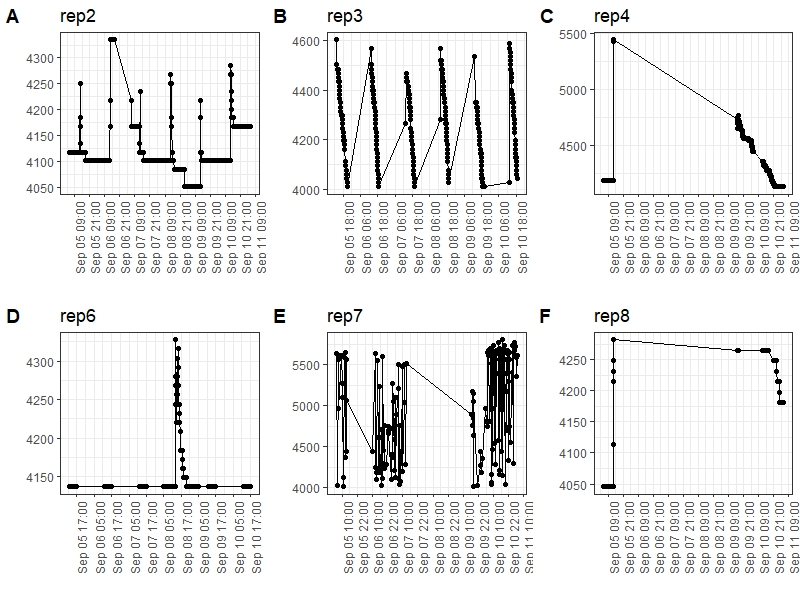


Figure S27. Weight measurements from the third growth stage for six (A to F) prototype replicas. Straight horizontal and diagonal lines correspond to lost data due to remote internet connection issues. It is important to mention that due to permanent remote connection failures, another prototype was removed from the experiment. Also, note to reach several days under greenhouse conditions, three prototype electronical boards (C, D, and F) failed to send data to the phenotyping platform; which caused the majority of data loss.


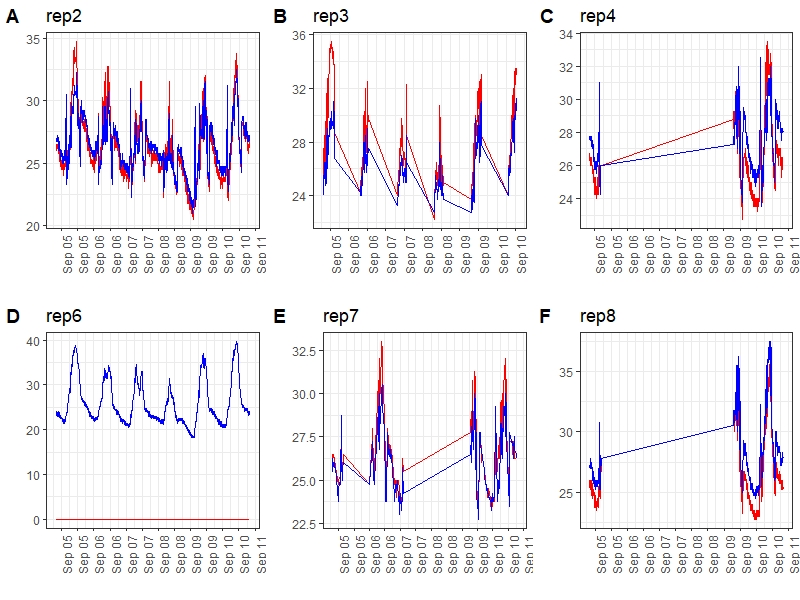


Figure S28. Temperature measurements from the third growth stage for seven (A to F) prototype replicas. Internal Stem T1 (red) and inside closer to pedicel T2 (blue). Straight horizontal and diagonal lines correspond to lost data due to remote internet connection issues. Note to the difference between sensors is high compared with the first stage; compared with the second stage, now for prototypes B, C, and E internal stem temperatures were higher than pod soil temperatures.

During this last experiment stage, issues with remote connection were even more visible. Two replicas (A and B) showed a few data transmissions during this period. The rest of the prototypes get automatically disconnected a major part of the time. However, despite constant noise, the amount of data stored from this period showed a continuation in behavior from the previous phase. This means water losses increased reaching between 400 and 500 ml per day approximately.

Data temperature was affected also by remote connection issues during the entire experiment. Therefore, there was not possible to count all possible data from this experiment. However, two cases that were more constantly sending data than others; showed another behavior. In magnitude now pod temperature was lower than pod temperature.

Additionally, Table S1 shows differences between temperatures measured (internal stem and pod) and the minimal and maximal point of these for replica 2 and environmental sensor DHT22.

Table S1. Data comparison for replica 2 (it was the most constant and with representative measurements during the entire experiment).

| Experiment | Stage 1 (15-20 Jul).  Air temperature measurements. | Stage 2 (15-20 Aug).  Internal stem (T1) and superficial inside soil (T2) temperature measurements. | Stage 3 (5-10 Sep).  Internal stem (T1) and inside closer to apice pod (T2) temperature measurements. |
| --- | --- | --- | --- |
| Mean of the difference’s values between two adapted temperature sensors (T1-T2) °C. | -0.62 | -0.77 | -0.74 |
| Maximum value (T1-T2) °C. | 1.75 | 1.75 | 3.5 |
| Minimum value (T1-T2) °C. | -1.5 | -4.25 | -5.75 |
| Total of times in which T1 measurements were higher than T2 (%). | 14.4 | 8 | 19.8 |
| Total of times in which T2 measurements were higher than T1 (%). | 85.6 | 92 | 80.2 |
| Mean of the difference’s values between the DHT22 sensor and adapted temperature sensor T1 (DHT22-T1) °C. | NA | 1.24 | 1.51 |
| Mean of the difference’s values between DHT22  sensor and adapted temperature sensor T2 (DHT22-T2) °C. | NA | 0.47 | 0.77 |
| Total of times in which DHT22 measurements were higher than T1 (%). | NA | 95 | 75 |
| Total of times in which DHT22 measurements were higher than T2 (%). | NA | 63 | 70 |
| Correlation between DHT22 temperature and T1 (%) | NA | 77 | 62.8 |
| Correlation between DHT22 temperature and T2 (%) | NA | 83 | 46.2 |

The last experiment showed the weak connection through Wi-Fi between each prototype replica and the internet’s network. Due to this, new corrections will be added to the gravimetric unit prototype to solve these issues. Besides, even the weighing system is still very susceptible to noise, and its measurements lack exactness and accuracy. However, data from temperature sensors showed good behavior most of the time. Thus, sensors integrated into replica two allowed seeing differences in the temperature inside the stem, pod, soil, and air (Table S1). Important behaviors from these data are, among others.

- No influence of air temperature (DHT22 sensor) on internal pod temperature.
- Internal stem temperature has a big direct correlation with air temperature (DHT22 sensor).
- Internal stem temperature is lowest than pod and air temperature (DHT22 sensor).

Thus, the last experiment also showed the importance of measuring these variables to evidence known or expected behaviors within abiotic processes.

### Third Gravimetric Unit prototype version – issues corrections to 2^nd^ version

Two main features must be corrected from the last experiment in order to reach stability and standardize the process. Lost data due to Wi-Fi issues and conflicts with ESP32’s internal libraries. On the other hand, weighing measurements needs the use of an improvement on load cell signal amplification less susceptible to external noise.

#### Use of New Computing Board

ESP32’s board was replaced with Raspberry PiZero W. This new board will manage.

- Data collection schedule and communication with Atmega328p.
- Data local storing.
- Data sending process to phenotyping platform through Wi-Fi.
- Atmega328p’s remote reprogramming through serial communication.
- Water irrigation control.
- Individual prototype hosting user application and interface.

Raspberry Pi is used with a lite version operative system developed for Raspberry [20] which is good enough for the prototype’s purposes. Implementation of this OS will guarantee the optimal working of the system. Besides, it is compatible with Node-red services, which allow easy user application programming and replication. Therefore, this device is capable to keep stability minimal locally in cases of intermittent Wi-Fi coverage. Thus, Raspberry Pi Zero W will request and store locally in a .csv file all data from different sensors and it is open to new digital sensors.

#### Load Cell data amplification improvement for Weighing Measurements.

Since Wheatstone bridge strain gauges are sensitive to thermal instability and mainly noise among others [21]. And previous tests have demonstrated this argument. Therefore, a new device was implemented focused on weight data amplification with noise reduction and easy use. Thus, 24 bits ADS1232 analog to digital converter developed by Texas Instruments was added to the prototype design.

ADS1232 is an ADC developed for multiple uses with a programmable gain. In this case, it adapts perfectly to the prototype’s design, is low cost, and with the library developed [22] is possible to use it with the Atmega328p microcontroller. A 128 gain was selected for output signal amplification with an 80 SPS data rate. Additionally, using SPI communication [23] was connected to the microcontroller I/O pins.

However, before implementing within an electronical embed board system. ADS1232 was tested to check its performance.

#### Corrected weighing system vs precision scale comparison

Thus, we decided to compare GU with standard commercial scale values (Trumax Precision Scale -TPS). Interestingly, both systems (Prototype and TPS) demonstrated some variations (using constant metal weight) in time without any electrical rebooting (Figures S29 and S30 respectively). This means that the signals of electronic converters are affected by continuous working. Therefore, the automatic-rebooting system was included in the prototype, to guarantee that electronically sensitive systems will not be affected by continuous working periods, and the measured value will respond to real weights (Figure S31).

For testing the new load cell signal amplifier electronical circuit, was implemented additionally two low pass RC filters in the ADC inputs to try to reduce external high frequencies noise. Subsequently, the new improved features were integrated into one prototype’ system and, placed inside a greenhouse. Additionally, in order to compare weighing system behaviors, the Trumax Precision Mix-A (3000 gr max) scale (TPS) [20] without a windbreaker was used close to the prototype under the same conditions. RS232 communication was used with a laptop to store data from Trumax Precision Scale at 9600 baud during the experiment. Thus, two experiments were carried on for comparing these weighing systems. In addition, environment temperature was also measured.

##### Validation of prototype working with Weight Fixed Non-Changing and Manual electrical reboot

The experiment consisted of putting over the top of both, the prototype and TPS, two different fixed known weights (iron pieces non-changing weight) 5596 gr for 15 days and 2371 gr for 8 days respectively. As weight over each one does not change, measurements neither should do it. Prototype measurements were however slightly modified since day 2 (Figure S29). Where the electronical board was electrically turned OFF for a few seconds and then turned ON again. This idea is based on the purpose to recalibrate periodically after each electrical source switching. On the other hand, TPS was left working for 8 days period without any external interruption (Figure S30).


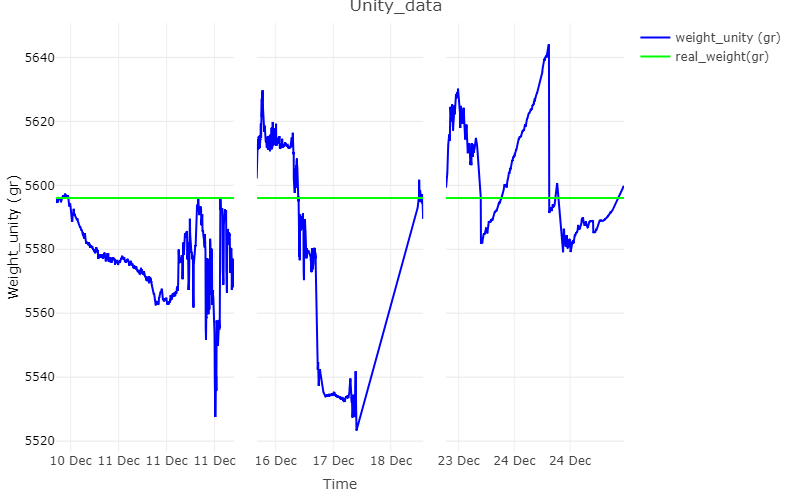


Figure S29. Fixed (non-changing) weight six days experiment with improved weighing system, manually ON/OFF switching and re-calibration every day. Note to Blue is data measured by the prototype and green it’s the real weight.


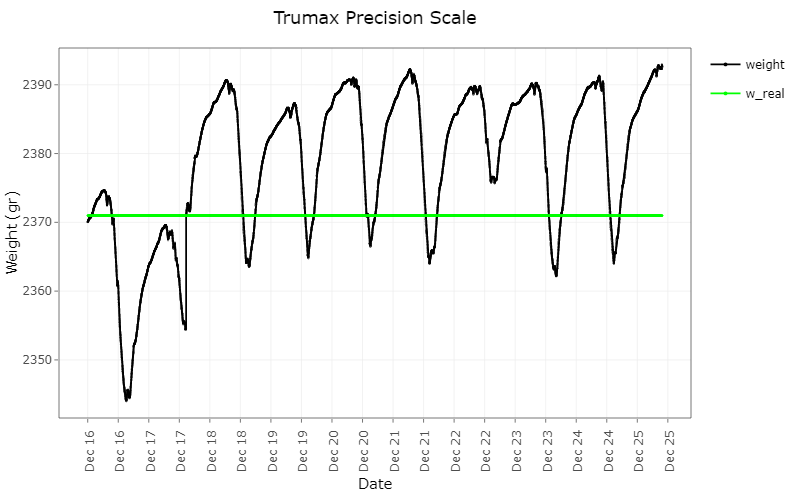


Figure S30. 9 days experiment. Fixed (non-changing) weight, measurements from Trumax Precision Scale. Note that Black is data measured by the prototype and green it’s the real weight.

Prototype weight measurements showed good accuracy as data were close between them. However, note to in the first part measurements lost exactness during the time (first day), even the weight measured reached 36 gr of difference above of real weight. Additionally, note to since this day, sudden changes in weight reaching real or reference weight appeared. This is because these were the times when the system was electrically reset and manually calibrated.

In the Trumax Precision Scale case, behavior also showed exactness losses reaching 27gr difference above real weight, but accuracy remains. However, its behavior was periodically constant during the experiment curse. Thus, it is possible to note to weighing systems are directly influenced by external factors in greenhouse conditions.

In addition, using temperature data some correlations were made to establish influences from this variable on weight prototype measurements (Table S2). Where it is possible to note, among others, that differences between real and measured weight from the prototype reached 23 gr average during the experiment.

##### Validation of prototype with Fixed Non-Changing Weight and automatic electrical reboot

Based on the previous experiment new improvement was implemented to the electronical board. Using a 3V relay source electrically turning OFF and ON were switched in a controlled way. Thus, the recently integrated Raspberry Pi Zero W managed the electrical power source connection of the Atmega328p and weight sensor circuit. The schedule to make this consisted of keeping electrically disconnected the before-mentioned system for 5 minutes, then turning ON the connection, loading the initial obtained calibration parameters, and measuring the current weight.

To test the new improvement, an experiment was carried out using previously used non-changing weight elements with one prototype (Figure S31) with Trumax Precision Scale (Figure S32) for 9 and 4 days respectively.

Before starting the test, the prototype’s calibration parameters were obtained and saved locally in EEPROM memory. These parameters were found measuring with prototype two different known weight points. The first parameter was obtained by making a measure without weight to know the raw corresponding data (OFFSET value). In addition, a known weight value was measured to know its raw corresponding data (SCALE value). Thus, these parameters were pre-loaded after each turned on before weight measurements. In addition, after the second test day, for the working prototype verification, an extra known fixed weight was added to the current 5596 gr, reaching 6106 gr to see behaviors with another weight introduced.


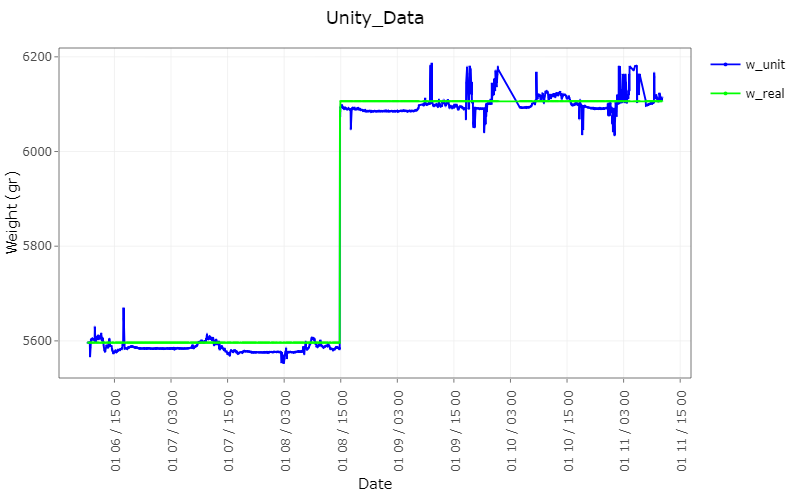


Figure S31. Five days of data from the experiment with the prototype. Two different fixed weights. Auto turning ON/OFF electrical connection switching every five minutes. Note to Blue is data measured by the prototype and green it’s the real weight.


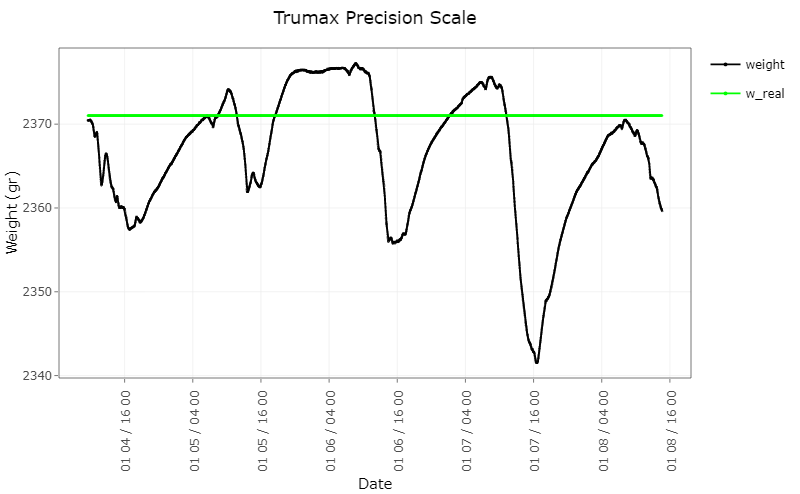


Figure S32. Four days of data from an experiment with the Trumax Precision Scale. Continuous working with one fixed weight. Note that Black is data measured by the prototype and green it’s the real weight.

From this experiment, it is important to note to for prototype’s weight measurements (Figure S31) have a kind of constant behavior with high accuracy, but exactness fails with approximately 20 gr of difference from the real weight. Besides, noise presence still appears (high peaks). However, measurements remain logical behaviors and low power consumption. Additionally, weight measurements correlated with temperature decreased compared with the previous experiment.

On the other hand, TPS showed lost exactness and even accuracy probably affected by external variables (Figure S32). Besides, trending seems further away from real weight during the time, which shows the importance of switching electrical source disconnection and reconnection process accompanied by a re-calibration each time. In addition, comparisons between the last and previous experiments are shown in Table S2, where it should be noted the reduction in temperature influences the weighing prototype system.

Table S2. Improved weighing system comparison with Trumax Precision Scale in greenhouse conditions.

| Experiment | The mean value from the absolute difference between weight real and measured for prototype (gr) | The mean value from the absolute difference between weight real and measured for Trumax Precision Scale (gr) | The maximum value of temperature’s influence on weighing prototype measurements (%) | The maximum value of temperature’s influence on weighing TPS measurements (%) |
| --- | --- | --- | --- | --- |
| Weight Fixed Non-Changing and Manual electrical reboot | 23.50 | 11.8 | 35 | 30.45 |
| Fixed Non-Changing Weight and automatic electrical reboot | 14.7 | 6.8 | 11.5 | 21.14 |

However, the non-changing weight test is an overview of weighing improved system behaviors under greenhouse conditions. Another important test to evaluate this is using a pot-soil-plant system together with the prototype. Thus, an experiment was carried on over 20 days with a bean plant genotype previously sown to evaluate system performance in the usual life cycle of gravimetric unit prototypes.

However, weighing measurements still showed some non-logical behavior under experimental conditions. This means that, unfortunately, other external variables can influence sensor precision until the constant weight test is done. Thus, looking for a responsive system under the most critical environments, temperature influence on load cell output values was tested too. Where these tests made to previous prototype versions, showed that the correlation between air temperature and weight measurement behaviors (with non-changing weight) can even reach 60% (Table S3). Therefore, the tiny and low-cost (TMP36) temperature sensor to measure the temperature of exposed electronical devices was added into the final electronic design (Figure S36) and finally integrated into the final prototype version (Figure S37) in order to reduce the noise of the external factors

Secondary variables integrated into prototypes were air temperature and air RH together with tiny sensors able to measure the temperature of different specific points (such as inside plant stem and pod, and in soil). These variables should facilitate comparing and quantifying physiological behavior in different environments.

#### Weighing system joint with the soil-plant system - comparison with temperature behavior

Last experimental verification of the new weighing system amplification and computing board. Was carried on testing the behavior of the system with improvements included (turning ON/OFF switching process) with a plant. Thus, a SEF60 bean seed was previously sown in one pot and placed under greenhouse conditions. For this experiment, Kern compact 12000 gr scale [24] was used as in situ weight comparator. Testing consisted of automatically irrigating the plant every day during the entire life experiment cycle, subsequently, every five minutes prototype measured the weight of the system pot-soil and plant (Figure S33). Additionally, randomly a few days at different times, manually, remove the pot with the plant from the prototype and weigh it using the Kern scale to compare real weighed data with measured data at that moment, at the end return the pot over the prototype again (Figure S34).


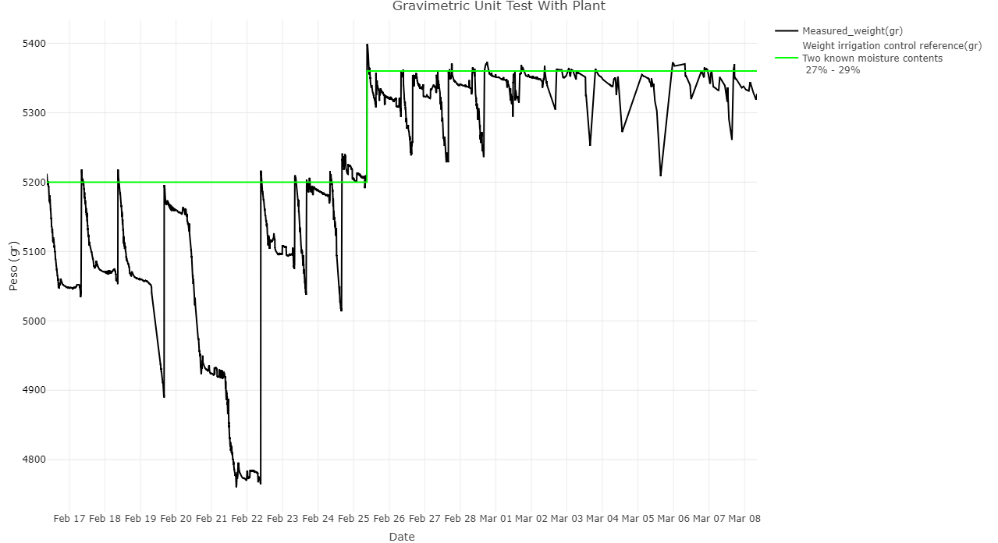


Figure S33. Data from one prototype with soil and plant (black lines) - 15 days of the experiment with two target weight points for automatic irrigation (green lines).


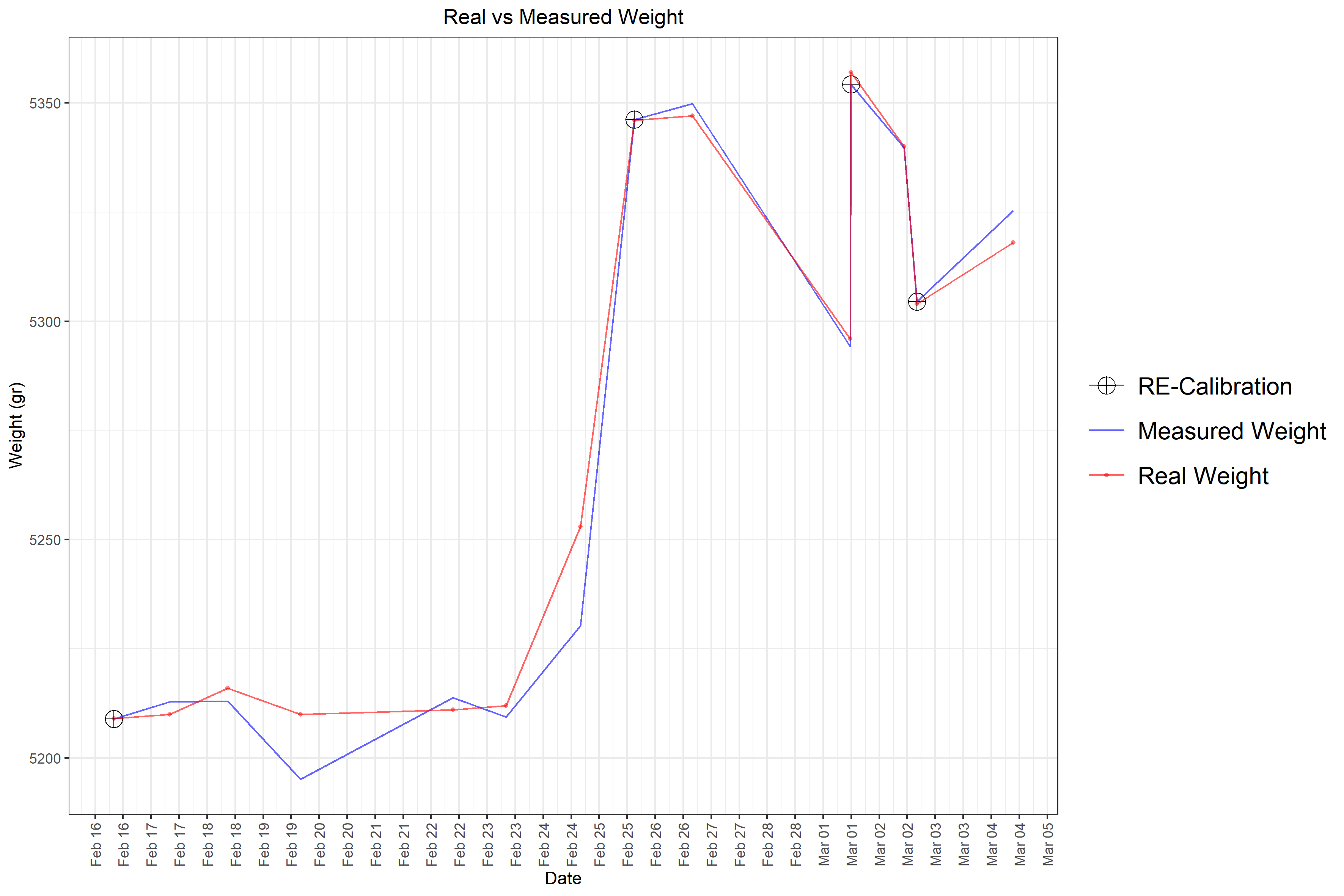


Figure S34. Data comparison for random measurements between real weight measured (red) with Kern precision scale and data from the prototype (blue) at the same time. Additionally, random manual re-calibration points were added (black markers).

In addition, at four random different times during the experiment, the weighing system was manually re-calibrated to correct the system’s exactness (Figure S34). This involved a new parameter-obtaining process for each re-calibration time. Each parameter was substantially different compared to its previous version in each calibration. This shows the possible influence on the weighing system from environmental behavior, most exactly temperature. Thus, the temperature was compared with absolute differences between real and measured weight (Figure S35) together after one re-calibration time and before another.

For the prototype, parameters acquisition was made at a specifically determined time. This means OFFSET and SCALE values correspond to the measurements for the different weights at that specific time and temperature at that moment. Therefore, calibration parameters and subsequently weight measurements' exactness could change as the temperature does. Thus, the temperature sensor was included within the embedded electronical board to measure this variable behavior near the electronical system which could be influenced by this factor.


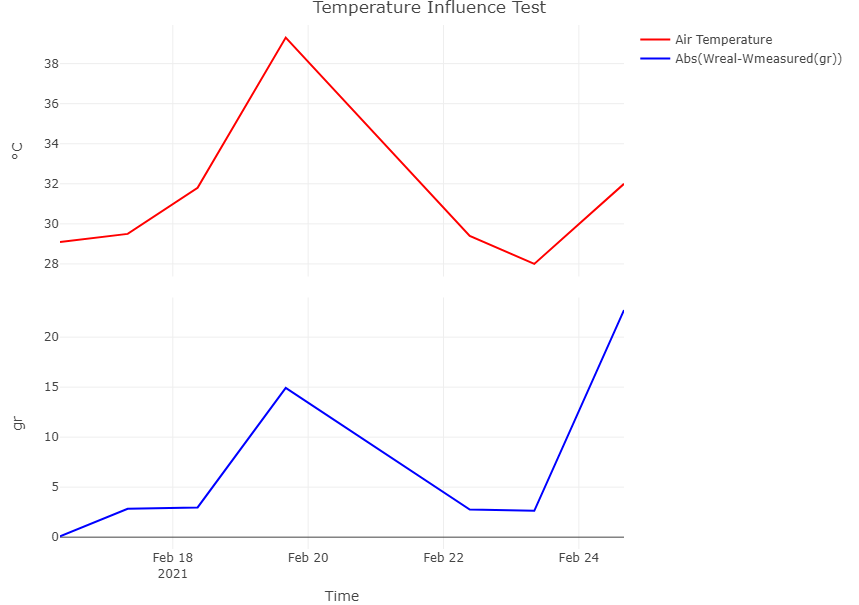


Figure S35. Data comparison between air temperature (red) and absolute differences (blue) value (real–measured weight) for one randomly manual re-calibration and measurements cycle with Kern precision scale and prototype at the same time.

Data from (Figure S33) showed good behavior from the prototype in logical terms. Irrigation control (green) every day was close in magnitude to the reference target point. However, it was unknown if every data had exactness, for this, it is possible to note (Figure S34) differences between real and measured by prototype weight. Additionally, Table S3 shows an average magnitude of differences values and mean value of air temperature influence on weight loss after irrigation for a few days.

Despite, the correct working of switching ON/OFF process. Manual re-calibration (new calibration parameters obtained) helped the prototype to keep exactness or very low differences when the air temperature was near the same as in the last re-calibration time (Figure S35). Where it is possible to note the parameters were obtained at 29 °C and it is at that temperature value where weight differences are lower. Table S3 allow seeing the influence of temperature on weight measurements within this period.

Table S3. Some statistical correlations from the last comparison experiment of the improved weighing system for one gravimetric unit prototype.

| Correlations | Value |
| --- | --- |
| The mean value from the absolute difference between weight real and measured for prototype (gr) | 6.98 |
| The maximum value of temperature’s influence on weighing prototype measurements (%) | 60.7 |
| Real and measured weight correlation with manual recalibration during the entire experiment (%) | 99 |
| The mean value from everyday weight losses by evapotranspiration with temperature (%) | 51.2 |

### Final Gravimetric Unit prototype version - Improvement and new features to the previous version

New features were joined as improvements with the oldest elements that remained in the prototype to get the final electronical embedded system (Figure S36).

- Raspberry Pi Zero W replaces Esp32.
- ADS1232 for load cell data amplification (replaces AD620AN).
- Two Low pass RC filters.
- Control of the power electrical source connection (ON/OFF switching).
- Electronical board temperature measurements.
- Calibration temperature-based parameters for the weighing system.
- 6 digital and 4 analog input pins able to add new sensors and devices.

Thus, weighing measurements were corrected and external noise was minimized to make sure the best data quality.

#### Weight System Measurements

The weighing system (sensor, amplifier, and converter) returns the primary and most important variable in the measurements. This developed system plays a crucial role in precise irrigation control, as it gives feedback to the microchip. Besides, during the prototype preparation, we found out that the stability of the weight sensor can be compromised because of the noise present in these systems. We used algorithms and filters for correcting electronic signals to reduce noise as much as possible.

The load cell measurements are possible using the Wheatstone bridge concept. For this development, a 10 kg to 1 mV/V weight sensor was used. This concept is thoroughly used with strain gauges, that measure resistance changes and convert them to an electric signal. Some lysimeters are made using four load cells (half Wheatstone bridge each) but wired electrically as one complete Wheatstone bridge [25]. In our LysipheN prototypes, the load cell used in the weighing system has integrated the full Wheatstone bridge. The full bridge has four internal resistances. Thus, the weight changes are measured by voltage changes V, where this value is given by Equation 1.

Equation 1

$V= \left( \frac{R3}{R3+R4}-\frac{R2}{R1+R2} \right)Vex$

Note: V is the Wheatstone's bridge output (mV); Vex is the voltage supplied to the load cell (from the first Kirchhoff’s law)

Changes in the weight are obtained using the adjusted voltage signal amplification and calibration. It is important to avoid any contact with any components, to allow the correct freedom for load movement within the main structure (in the range of mm).

As the load cell works at a very low voltage level with 3-5 V input (1 mV in this case as an output signal) it is necessary to use the signal amplifier system (instrumentation amplifier) [21]. The ADS1232 analog-to-digital converter (ADC) was used for this purpose (Texas Instruments, USA). This ADC is thoroughly used in industrial instrumentation applications. Other solutions proposed the use of HX711 ADC to convert the signal from the sensor selected [26]. However, under greenhouse conditions (high temperatures or humidity), this element showed a higher noise susceptibility (Figure S16). Therefore, was selected for this development the ADS1232; which is a 24-bit ADC for weighing scales and is equipped with a programmable gain amplifier (PGA) [27]. Then, using the Serial Peripheral Interface (SPI) communication protocol, the ADC was connected to the SPI acquisition data’s microcontroller pins respectively using a GitHub library [22] developed by Ciorceri for the Arduino version.

The load cell output was connected to ADS1232 differential input using a simple RC filter as a low pass filter to try to reduce the external noise. Moreover, the ADC pins’ output was connected to the microcontroller input pins as was previously mentioned. Once data is read by the microcontroller, based on the calibration process it is converted to the weight in grams.

#### Main electronic board design

After the testing stage, a main electronic board was designed to integrate all electronic components into an embedded system. This design was made using the EASYEDA [28] software, which allows the design and building of an electronic schematic and PCB routing board, respectively. The printing circuit process was made by JLCPCB [16], using the previous design (Figure 5).

Atmega328p was connected to electronic elements such as a button in the RESET pin and a clock in the XTAL pins at 16MHZ with ceramic 22pF capacitors. In the same way, Raspberry Pi Zero W was connected using the micro-USB source input. The atmega328p microcontroller is connected to the Raspberry Pi Zero W using serial pins for communication and SPI pins for reprogramming it, using the remote connection that the Raspberry Pi Zero W allows (a crucial feature for any change in the working routine or adding sensors).

Inside the whole connections between these two main devices, to look for an optimized working of the system, a GPIO from Raspberry Pi Zero W, is connected to the Relay switch, which is responsible for turning on/off the Atmega328p. This step supports energy-saving and shelf-life cycle prolongation of some components.


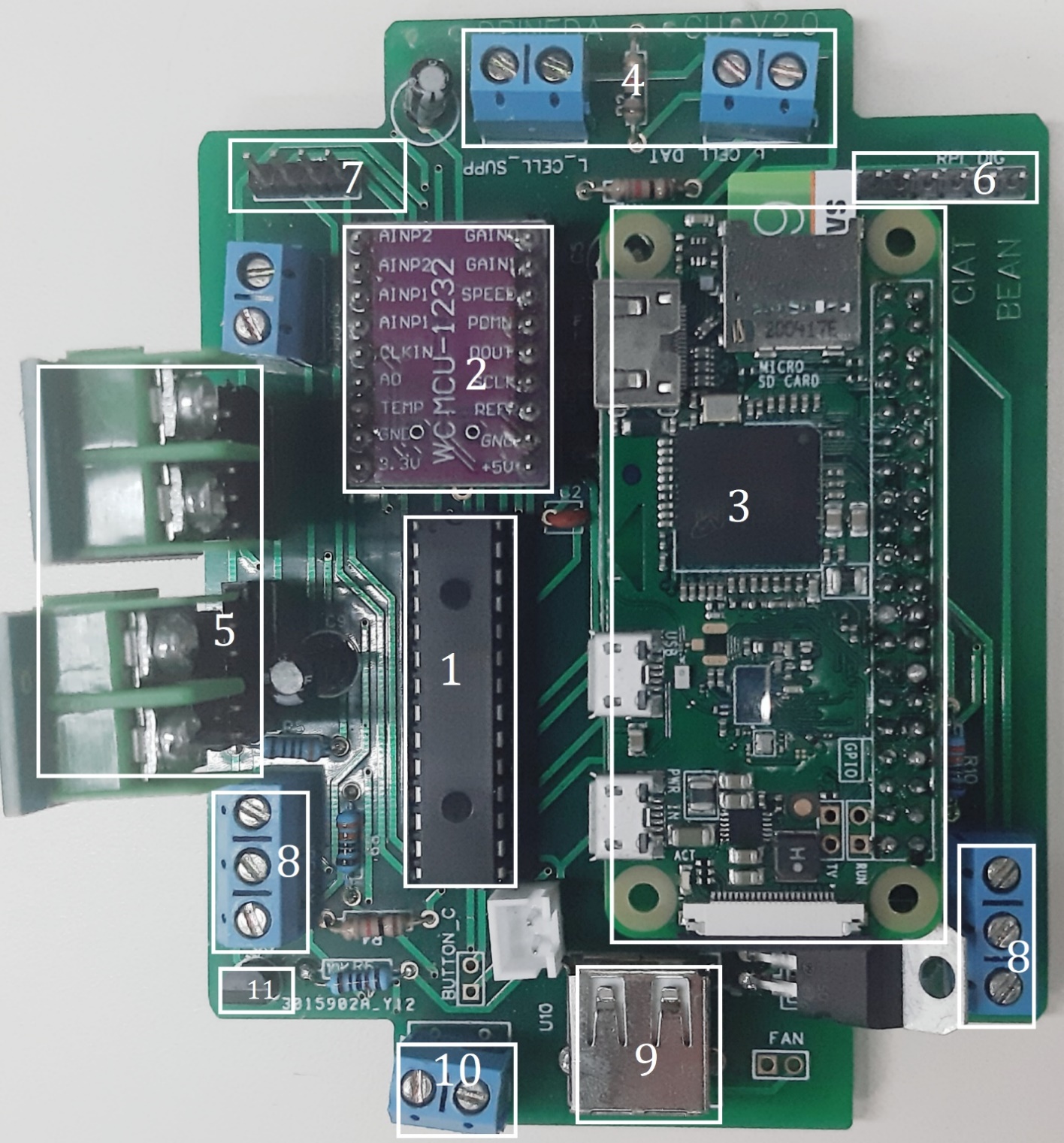


Figure S36. Main electronical board with all devices integrated (Top view). 1. Microcontroller. 2. ADS1232 (weight measurements converter). 3. Raspberry Pi Zero. 4. Four inputs for load cell (weight sensor) connection. 5. Two temperature sensors converters. 6. Six free digital input pins with electrical source pins. 7. Four free analog input pins. 8. Two connectors for relay controlling (irrigation and reboot control). 9. USB connector (only for internal Raspberry Pi use). 10. Electrical 5v input connector. 11. Tiny temperature sensor. Note that there are additional electronical components necessary for the correct working of the system (Resistors, voltage converters, capacitors, etc.).


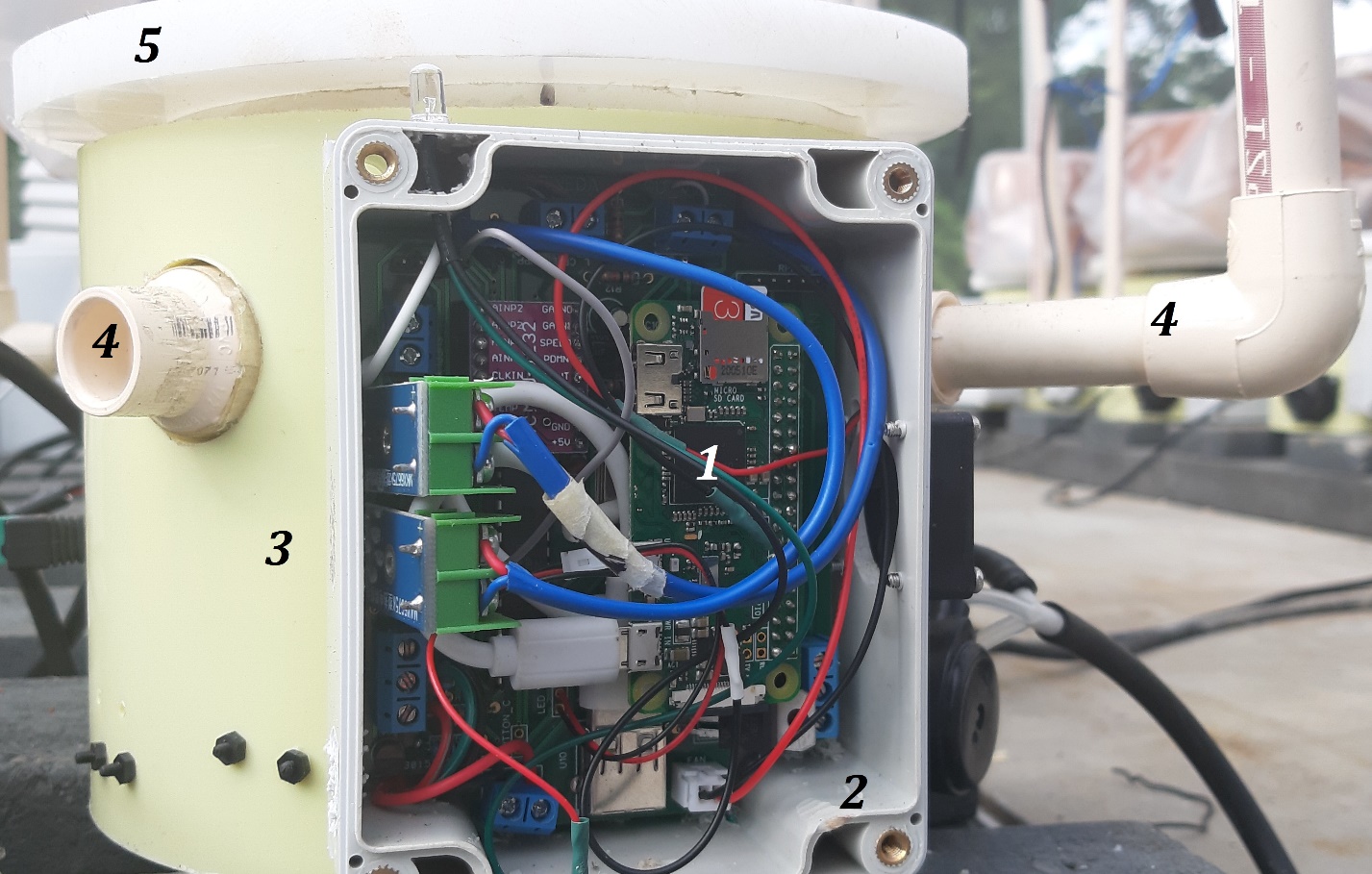


Figure S37. Main electronical board assembled into prototype protection box (front view). 1. Electronical mainboard. 2. Protection ip65 box. 3. Circular PVC base. 4. Output holes for connecting sensors and dripping irrigation tubes. 5. Teflon Base for placing pot.


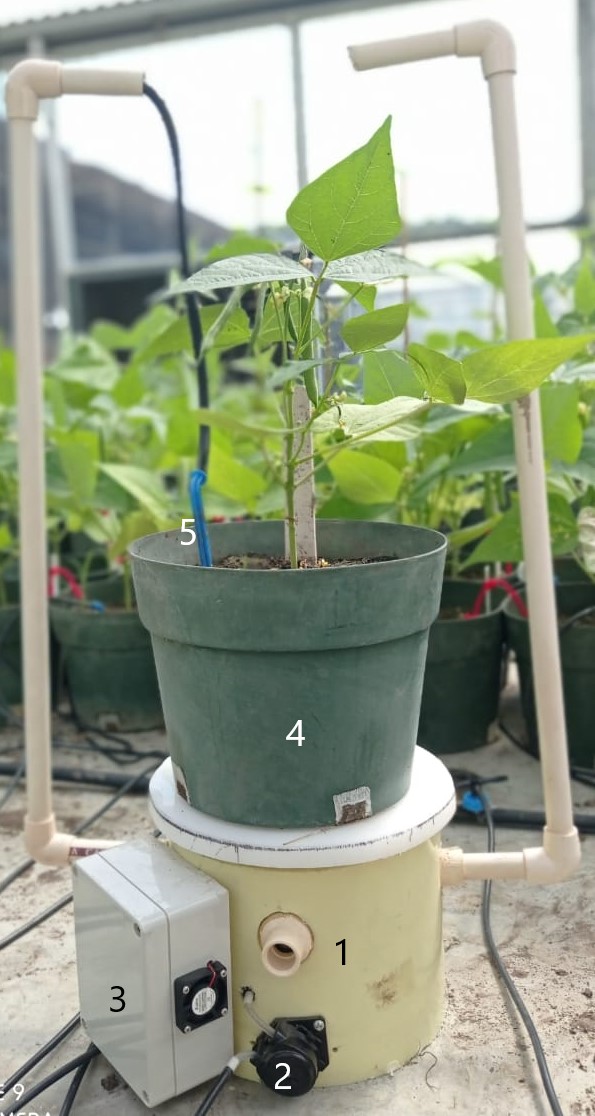


Figure S38. Final gravimetric unit prototype version. 1. PVC base. 2. Peristaltic for an irrigation pump. 3. Electronical protection box. 4. Pot with the plant to evaluate. 5. Irrigation dripping system.

#### Internal pod and stem temperature measurements

Each adapted thermocouple needs a signal amplifier system since the original output is in mV. To solve it, thermocouples were connected to the 12 bits analog-digital converter MAXX6675 [12], widely used to convert signals from similar sensors and used in different monitoring and controlling processes [13]. This module was connected to the microcontroller input pins using SPI communication and with the appropriate library. Finally, the raw data were converted to the temperature in ℃.

#### Self-Temperature compensation calibration

[3] used four load cells connected as a full Wheatstone bridge with a temperature-compensated system. The analog-to-digital converter datasheet (ADS1232, used for gauge signal amplification) showed a ratio between offset noise and temperature. Thus, despite changes in the nV term, temperature noise is a variable to correct in order to obtain more precise data. In our system, the temperature corrections are done as the electronic board temperature is also measured. For this purpose, the temperature sensor TMP36 [14] was used to measure the air temperature related to the microclimate to which the electronical board is exposed. The data from this sensor is used to run an auto-compensation temperature calibration algorithm for more precise weight measurements.

The calibration process was carried out for 4 days. This stage will guarantee that every single prototype will be able to correct any weight measurement based on the temperature measured by the tiny temperature sensor (TMP36).

Thus, first two days all 10 prototypes began without any weight to measure. So, OFFSET (zero without weight) value can be corrected by the temperature measured during this stage (Figure S39). In the second part, for two days, every single prototype had a known constant (non-changing weight, usually above 5000 gr). This allows obtaining a SCALE value necessary for weight measurements based on temperature (Equation 2).


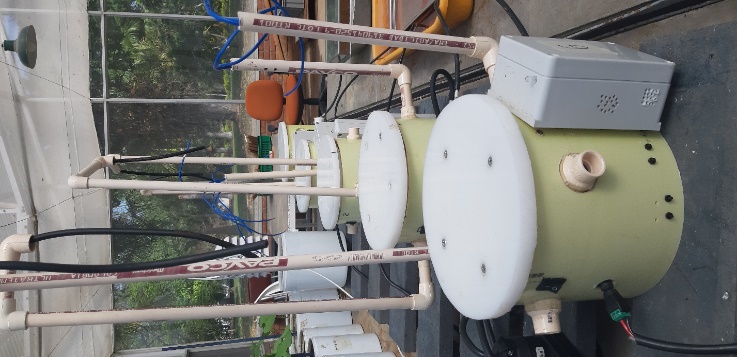


Figure S39. Temperature calibration stage - without weight (zero point).

Next, with data from the calibration stage, the following expressions were obtained based on lineal correlations between data from the weight sensor and temperature. Once got these expressions, the system will measure raw weight sensor data and temperature before giving a value in grams units.

Equation 2

$W\left( gr \right)=(RAW-OFFSET)/SCALE$

Where W represents weight in grams after every single measurement. RAW is the current measurement from the load cell (weight sensor). OFFSET is the value that is based on temperature expression (Equation 3) at zero weight. SCALE is the value obtained during expression from calibration at known constant weight with temperature (Equation 5).

Equation 3

$OFFSET=M*T+b$

Equation 2 represents the OFFSET value based on measurements from the ratio between the weight sensor and temperature readings for the first two days experiment. Where M is the slope or data tendency in the ratio (constant). T is the current temperature measurement and b is a constant value that depends on the interception point on the ratio between the raw measurements from the weight sensor at zero weight and temperature.

As mentioned before, the parameter OFFSET is the raw measurement by weight sensor without any weight to measure (zero) and depending on the temperature for the prototype. Thus, it was also possible to find raw depending on temperature value for a known weight P as a linear correlation between temperature and weight raw measurement (Equation 4) from the second two days experiment.

Equation 4

$$CWR= M\_cw*T+b\_cw$$

Then, Equation 3 is the ratio between temperature and raw measurements from the weight sensor at known constant (non-changing) weight, which gives as a result the CWR values (raw weight data at known W) depending on temperature T. Note, M_cw is the ratio’ slope which involves data tending, and b_cw is a constant value that depends on interception point on the ratio between the raw measurements from weight sensor at known constant weight and temperature.

Equation 5

$$SCALE=(CWR-OFFSET)/cW$$

Thus, to complete the unknown parameters to feed Equation 1, the SCALE value was obtained (Equation 4) as a factor that depends on values got on tests with zero (OFFSET) and known constant weight (CWR). Where cW is the value of the known constant weight in grams used to find CWR.

#### Water irrigation control

The consumed water can be replaced by plant irrigation while maintaining a nearly constant soil water status during the day. In addition, by gravimetric correction for the plant biomass increase, a water expenditure curve can be obtained. Besides, the irrigation process of the plant is crucial to perform normal plant growth, but also monitoring, and minimizing the service time [5]. In our study, we present that the LysipheN units have a simple but sophisticated automatic water irrigation system. Irrigation was supplied with high precision actuators (5-10 ml maximum of error) which were also used to support two different irrigation systems (dripping point from above the soil surface and classic bottom irrigation reaching first the roots). Understanding that the accuracy of using weighing lysimeters to keep the water balance in the soil will depend, among others, on the knowledge of the initial water content in the soil [5].

These data will serve as an important source of genotype-based variability as an effect of their water-limited behavior (water spenders, water savers), heat, and other stress resistance mechanisms of individual genotypes. As already mentioned, the full independence of individual units, the same variables can be measured in different environments, and data will get G x E x M dimensions. (Genotype X Environment X M?)

The implemented relay controller can turn the pump on/off, according to the irrigation scheme and the actual water content in the pot. This pump is a precise low-power consumption device. Besides, the peristaltic pump can be calibrated, to estimate a minimum water volume always present in tubes (not affecting soil water status) after each run of irrigation and depending on opening and closing times (in seconds). However, mostly this highest precision is not necessary, as the actual irrigated water volume can easily be estimated from the weight difference.

#### Electrical source connection and backup

Any experiment with LysipheN units needs an autonomous electrical and commonly used universal 12 V source. This allows its use at any location or country. However, continuous working of the device can be performed in order to secure the maximum quantity of data collection. In that way, if the voltage is supposed to fall in the main electrical network, it is necessary to have an electrical backup (Figure 2) to ensure an autonomous process. Therefore, all experiments involved a battery backup system, which in parallel, secured 100% of the time working for the ten replicas built. In this case, the electrical 120V network replaced the solar panel by using a 120 V AC to 12V DC adapter to charge the backup system. Thus, we used a 75 A/h electrical charge system [29]. With this parallel electrical source, at least 7 hours of continuous working without the main electrical network is guaranteed. Data are locally saved in a micro-SD card (type) integrated into each prototype [30]. When the main network is re-established, supplies the gravimetric units, and charges the backup system.

#### Data collection and control microcontroller

For an electronic system that requires sensor use, it is essential to have a microcontroller capable of reading and converting analog signals or digital messages into useful data. To solve this problem, the Atmega328p was selected [31]. This microcontroller is frequently used in different projects. In addition, because plenty of packages and libraries have been developed to be used with this device; it is possible the integration and communication this device with various sensors, and actuators, and even to develop boards such as Raspberry Pi.

Besides, the Atmega328p also includes a 10 bits resolution ADC, which allows the use directly of analog sensors if necessary.

#### Software

Based on mentioned by [5], the accuracy of the control system to maintain the water balance depends on the initial water content of the soil. Therefore, before each experiment, a calibration protocol should be carried out in terms of the preparation of a correct and precise watering scheme to approximate the water soil content in each system (temperature and moisture sensors are previously calibrated).

The total initial soil weight with known water soil can be defined by Equation 6:

Equation 6

$$Tws=Wsd+Wc$$

where Tws is the total weight of the system, Wsd is the weight of dry soil and Wc is water content in weight units. The user must define what will be the weight of the dry soil (it very likely this will vary for each pot) to be used during the experiment. Knowing this value, the soil water content can be calculated by (Equation 7):

Equation 7

$$Wc=Wsd*(Hw)$$

where Wc is water content in grams, Wsd is dry soil weight and Hw is the wanted humidity in percentage (%). Then, with these values, it is possible to know how many grams of initial soil and water the user must supply before starting the experiment. Once the test begins, the LysipheN prototypes will record this initial value and will try to control the irrigation (dripping mode) every day to keep the system stable according to the selected drought scenario (in the weight percentage of water losses by evapotranspiration or chronic/acute decrease in demand).

## Phenotyping Platform Improved and SQL database.

The first phenotyping platform version showed inconsistency in the data-saving process for the gravimetric units’ application. Since prototypes had Wi-Fi connection issues, the platform application programming routine stored the same data for hours which caused no real data saved. Thus, MySQL database and Node-red programming were used as an improvement to the phenotyping platform.

After the implementation and adjustment phase with continuous we could get an IoT-based greenhouse automation platform solution for monitoring and controlling the environmental conditions allowing the implementation of a database for data storage and query, real-time graphics, and remote access to the application by VPN to provide greater security in this second version (Figure S40) was done.


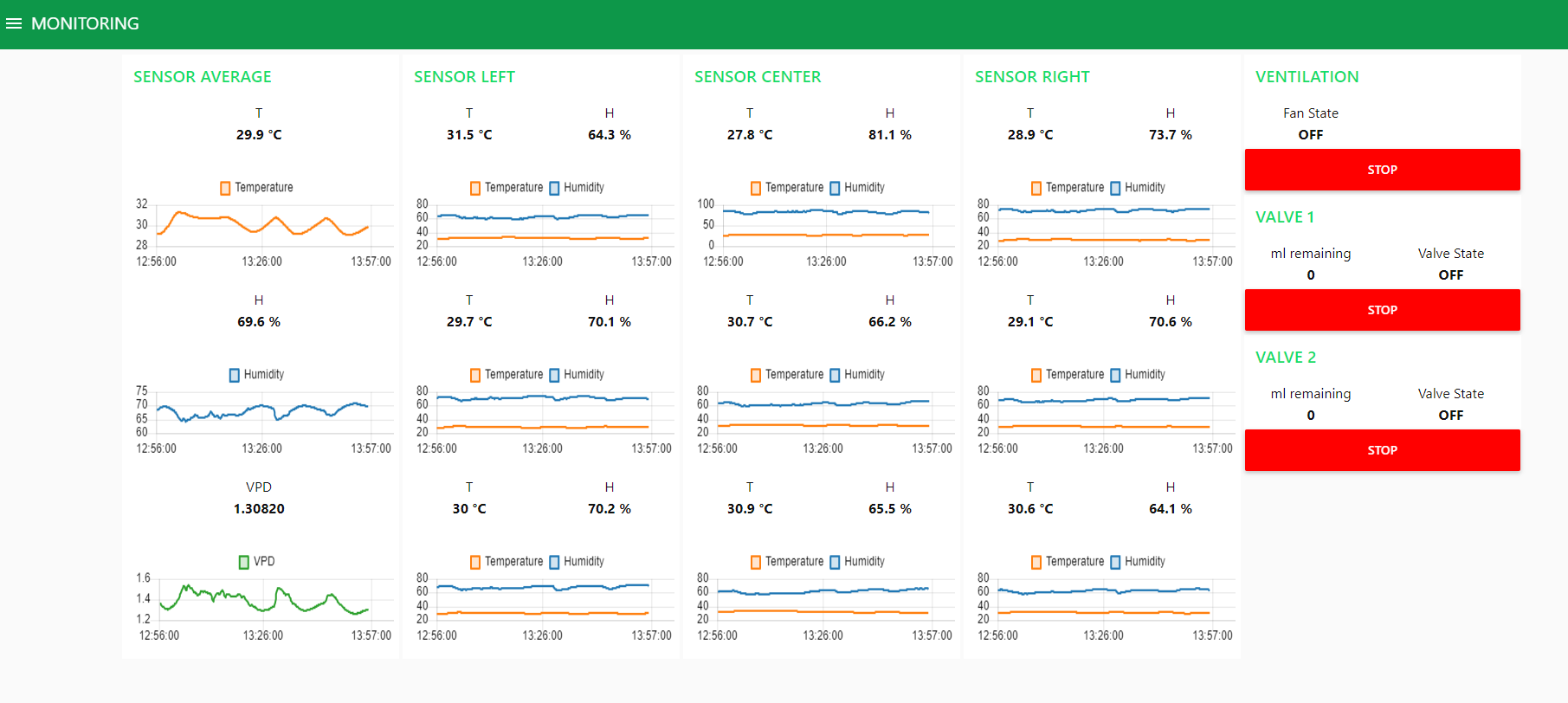


Figure S40. Bean Phenotyping Platform – last version.

## References

1. HX711 Arduino Library - Arduino Reference [Internet]. [cited 2021 Jun 8]. Available from: https://www.arduino.cc/reference/en/libraries/hx711-arduino-library/

2. Rosadi A, Fauzan A, Winarno. Prototype design of automatic plant watering equipment with soil moisture detection system based on arduino uno microcontroller: Case study of chili plant. J Phys Conf Ser. Institute of Physics Publishing; 2020.

3. Jiménez-Carvajal C, Ruiz-Peñalver L, Vera-Repullo JA, Jiménez-Buendía M, Antolino-Merino A, Molina-Martínez JM. Weighing lysimetric system for the determination of the water balance during irrigation in potted plants. Agric Water Manag. 2017;183:78–85.

4. Poorter H, Fiorani F, Stitt M, Schurr U, Finck A, Gibon Y, et al. The art of growing plants for experimental purposes: A practical guide for the plant biologist. Functional Plant Biology. 2012. p. 821–38.

5. Vera-Repullo JA, Ruiz-Peñalver L, Jiménez-Buendía M, Rosillo JJ, Molina-Martínez JM. Software for the automatic control of irrigation using weighing-drainage lysimeters. Agric Water Manag. 2015;151:4–12.

6. Peristaltic Liquid Pump with Silicone Tubing - 12V DC Power– The Pi Hut [Internet]. [cited 2022 Feb 9]. Available from: https://thepihut.com/products/peristaltic-liquid-pump-with-silicone-tubing-12v-dc-power

7. Diaz H, Dorado C, Angulo O, Escobar R, Tohme J. Build and implementation of a low-cost irrigation system for cassava hardening phase and greenhouse management [Internet]. 2018. Available from: https://ciat.cgiar.org/event/istrc2018/

8. Zhao X, Lu X, Lu W, Jin C, Zhang X, Zhang G. A signal conditioning preposing circuit for portable wheel load measure plate. Applied Mechanics and Materials [Internet]. Trans Tech Publications Ltd; 2013 [cited 2021 Jun 8]. p. 95–8. Available from: https://www.scientific.net/AMM.274.95

9. Lee CY, Yang CW, Fu LM. Design and fabrication of MEMS-based tire pressure sensor. Proceedings of the World Congress on Mechanical, Chemical, and Material Engineering. Avestia Publishing; 2016.

10. GitHub - adafruit/MAX6675-library [Internet]. [cited 2021 Jun 9]. Available from: https://github.com/adafruit/MAX6675-library

11. uxcell K-Type Thermocouple Wire 2 Core AWG 24 High Temperature PTFE Insulation 6.6ft- Buy Online in Bermuda at Desertcart - 175775803. [Internet]. [cited 2022 Feb 8]. Available from: https://bermuda.desertcart.com/products/175775803-uxcell-k-type-thermocouple-wire-2-x-0-5-mm-stranded-wire-blue-ptfe-extension-wire-2-meters

12. MAX6675 K-Thermocouple to Digital Converter Module [Internet]. [cited 2022 Feb 8]. Available from: https://www.cytron.io/p-max6675-k-thermocouple-to-digital-converter-module

13. Nurhuda A, Harpad B. Prototype of Fan Radiator Control System Diesel Machine Using Max6675 Type Transmitter and Type-K Thermocouple Sensor Based On Arduino Uno.

14. Temperature Sensor - TMP36 - SEN-10988 - SparkFun Electronics [Internet]. [cited 2022 Feb 8]. Available from: https://www.sparkfun.com/products/10988

15. EAGLE | PCB Design And Electrical Schematic Software | Autodesk [Internet]. [cited 2021 Jun 9]. Available from: https://www.autodesk.com/products/eagle/overview?term=1-YEAR

16. PCB Prototype & PCB Fabrication Manufacturer - JLCPCB [Internet]. [cited 2021 Jun 1]. Available from: https://jlcpcb.com/

17. Reicosky DC, Millington RJ, Klute A, Peters DB. Patterns of Water Uptake and Root Distribution of Soybeans ( Glycine max .) in the Presence of a Water Table 1 . Agron J [Internet]. 1972 [cited 2021 Jun 10];64:292–7. Available from: https://acsess.onlinelibrary.wiley.com/doi/full/10.2134/agronj1972.00021962006400030011x

18. Poyatos R, Granda V, Flo V, Adams MA, Adorján B, Aguadé D, et al. Global transpiration data from sap flow measurements: The SAPFLUXNET database. Earth Syst Sci Data. 2021;13:2607–49.

19. Jose JC, Joao CCS, Marconi BT, Jose AJ, Melissa SDC, Ana COH, et al. Growth and production of common bean in direct seeding under irrigated deficit condition. Afr J Agric Res. 2016;11:2841–8.

20. Balanza Trumax Mix A [Internet]. [cited 2022 Feb 9]. Available from: https://www.bci.co/productos/balanza-de-mesa-trumax-mix-a

21. Muller I, Machado De Brito R, Pereira CE, Brusamarello V. Title Byline Load Cells in Force Sensing Analysis-Theory and a Novel Application A Ring-type Load Cell. 2010.

22. GitHub - ciorceri/ADS1232: ADS1232/ADS1234 Arduino Library [Internet]. [cited 2021 Jun 1]. Available from: https://github.com/ciorceri/ADS1232

23. Serial Peripheral Interface - an overview | ScienceDirect Topics [Internet]. [cited 2021 Jun 15]. Available from: https://www.sciencedirect.com/topics/computer-science/serial-peripheral-interface

24. Kern. Instrucciones de servicio Balanza compacta KERN FCB [Internet]. Available from: www.kern-sohn.com

25. Silva AL, Varanis M, Mereles AG, Oliveira C, Balthazar JM. A study of strain and deformation measurement using the Arduino microcontroller and strain gauges devices. Revista Brasileira de Ensino de Fisica. 2019;41.

26. Liyanage DK, Chathuranga I, Mori BA, Thilakarathna MS. A Simple, Semi-Automated, Gravimetric Method to Simulate Drought Stress on Plants. Agronomy. 2022;12.

27. ADS123x 2-and 4-Channel, 24-Bit, Delta-Sigma ADCs for Bridge Sensors [Internet]. 2021. Available from: www.ti.com

28. EasyEDA – Online printed Circuits Design and Simulator [Internet]. [cited 2021 Jun 1]. Available from: https://easyeda.com/

29. AGM battery for photovoltaic motorhome Green Cell 12V 75Ah gel [Internet]. [cited 2022 Feb 9]. Available from: https://greencell.global/en/ups-and-agm-accumulators/2357-green-cell-agm-12v-75ah-vrla-battery-gel-deep-cycle-powerchair-photovoltaic-leisure-battery-campervan.html

30. SanDisk Ultra 32GB UHS-I/Class 10 Micro SDHC [Internet]. [cited 2022 Feb 9]. Available from: https://www.walmart.com/ip/SanDisk-Ultra-32GB-UHS-I-Class-10-Micro-SDHC-Memory-Card-with-Adapter-SDSDQUAN-032G-G4A/180605232?athbdg=L1700

31. ATmega328 8-Bit AVR MCUs - Microchip Technology | Mouser [Internet]. [cited 2022 Feb 9]. Available from: https://co.mouser.com/new/microchip/atmelatmega328/
